# Comparison of the modified unbounded penalty and the LASSO to select predictive genes of response to chemotherapy in breast cancer

**DOI:** 10.1371/journal.pone.0204897

**Published:** 2018-10-01

**Authors:** Olivier Collignon, Jeongseop Han, Hyungmi An, Seungyoung Oh, Youngjo Lee

**Affiliations:** 1 Luxembourg Institute of Health, Competence Center for Methodology and Statistics, Luxembourg; 2 Department of Mathematics, Korea Military Academy, Seoul, South Korea; 3 Department of Statistics, College of Natural Sciences, Seoul National University, Seoul, South Korea; Istituto di Ricovero e Cura a Carattere Scientifico Centro di Riferimento Oncologico della Basilicata, ITALY

## Abstract

Covariate selection is a fundamental step when building sparse prediction models in order to avoid overfitting and to gain a better interpretation of the classifier without losing its predictive accuracy. In practice the LASSO regression of Tibshirani, which penalizes the likelihood of the model by the L1 norm of the regression coefficients, has become the gold-standard to reach these objectives. Recently Lee and Oh developed a novel random-effect covariate selection method called the modified unbounded penalty (MUB) regression, whose penalization function can equal minus infinity at 0 in order to produce very sparse models. We sought to compare the predictive accuracy and the number of covariates selected by these two methods in several high-dimensional datasets, consisting in genes expressions measured to predict response to chemotherapy in breast cancer patients. These comparisons were performed by building the Receiver Operating Characteristics (ROC) curves of the classifiers obtained with the selected genes and by comparing their area under the ROC curve (AUC) corrected for optimism using several variants of bootstrap internal validation and cross-validation. We found consistently in all datasets that the MUB penalization selected a remarkably smaller number of covariates than the LASSO while offering a similar—and encouraging—predictive accuracy. The models selected by the MUB were actually nested in the ones obtained with the LASSO. Similar findings were observed when comparing these results to those obtained in their first publication by other authors or when using the area under the Precision-Recall curve (AUCPR) as another measure of predictive performance. In conclusion, the MUB penalization seems therefore to be one of the best options when sparsity is required in high-dimension. Further investigation in other datasets is however required to validate these findings.

## 1. Introduction

When building prediction models, covariate selection is a fundamental step in order to maximize the interpretability of the classifier and to avoid overfitting [1–5] while maintaining the predictive accuracy. In particular, sparse models, i.e. including a very limited number of covariates, are very attractive because their fitting depends only on the estimation of a few parameters, offering an easier interpretation of the model. In practice, the financial cost of these models is also potentially lower since only a few covariates are to be measured to accurately classify a new individual. Indeed, in medicine and biology for example, predictive biomarkers can be very costly to measure and therefore the larger the number of covariates needed in a predictive model the higher its effective cost. In this respect, the LASSO regression has become the gold standard for covariate selection [5, 6]. This method is based on a penalization of the likelihood of the model by the L1 norm of the vector of the regression coefficients of the covariates. Indeed, non-differentiability of the penalization function at 0 enables to produce sparse selection. Variable selection methods based on likelihood penalization also encompass the Elastic Net penalty [7] and the Smoothly Clipped Absolute Deviation (SCAD) penalty [8]. Bayesian alternatives such as spike and slab, and the Bayesian LASSO are also available [9–11]. More recently Lee and Oh [12] developed a novel random-effect covariate selection method called the MUB regression, whose penalization function can equal minus infinity at 0. This method offered promising results in simulations and in toy datasets [13, 14] and therefore deserves further practical investigation.

The objective of the present work is to compare the performances of the LASSO and the MUB in practice when applied to high-dimensional data. To this end, the datasets previously described and analysed by de Ronde et al [15] are an interesting case study because of the very large number of covariates measured and their conclusive sample size (i.e. sufficiently large to make prediction feasible). More precisely, this database is a collection of four different datasets, which consist in gene expression profiles measured to predict response to chemotherapy in breast cancer patients. Using the data shared by the authors, our goal was to build the best predictor of response to chemotherapy using the shortest list of discriminant genes in each of the four datasets as well as in the pooled database. We especially sought to compare both the number of covariates selected using the LASSO and the MUB as well as the predictive accuracy of the corresponding models, as measured by the AUC of the related classifiers. In order to compare both variable selection methods using another performance criterion as a sensitivity analysis, their respective AUCPR were also computed [16]. Finally we also compared our results to those published previously by de Ronde et al [15].

The paper is organized as follow. In the first section, we remind the reader about how random-effect covariate selection can be used to derive penalized regressions such as the LASSO and the MUB and we describe how these methods relate to other variable selection methods available in the literature. This is then followed by a description of the datasets being considered. The statistical analysis planned to compare both penalization methods is then described in details. We also explain how our findings were compared to the results of de Ronde et al [15]. In the following sections the results of the different experiments are reported and discussed before we finish with concluding remarks.

## 2. Material and methods

### 2.1 Random-effect covariate selection and penalized regressions

Lee and Oh [12] introduced a random-effect model that leads to a family of penalized likelihood estimators, including the ridge, LASSO and MUB estimators.

For a set of observations indexed by 1 ≤ *i* ≤ *n*, consider the following random-effect model:

$$Y_{i}=x_{i,1}\beta_{1}+\cdots x_{i,p}\beta_{p}+e_{i},\;e_{i}∼N(0,\phi ),$$

where for 1 ≤ *i* ≤ *n*, *Y*_*i*_ is the response variable, *x*_*i*_ = (*x*_*i*,1_,…,*x*_*i*,*p*_) is the vector of the covariates, *β*_1_,…,*β*_*p*_ are the regression coefficients and *ϕ* > 0 the variance of the error term *e*_*i*_. Suppose that conditionally on *u*_*j*_, we have for 1 ≤ *j* ≤ *p*,

$$\beta_{j}|u_{j}∼N(0,u_{j}\sigma ),$$

where σ > 0 is a fixed dispersion parameter, and *u*_*j*_’s are an iid sample of a gamma distribution with rate and shape parameters both equal to 1/*τ* > 0, such that the density function *f*_*τ*_ can be written

$$f_{\tau }(u_{j})={\left(1/\tau \right)}^{1/\tau }\frac{1}{\Gamma (1/\tau )}u_{j}^{1/\tau -1}e^{-u_{j}/\tau },$$

with E(*u*_*j*_) = 1 and Var(*u*_*j*_) = *τ*.

In this random effect model, sparsity of the selection can be achieved in an apparent way, since if the random effect estimate *u*_*i*_ ≈ 0 then *β*_*j*_ ≈ 0. Note that σ*u*_*j*_ = (*a*σ)(*u*_*j*_/*a*) for any *a* > 0, which means *σ* and *u*_*j*_ are not separately identifiable and justifies the constrain *E*(*u*_*j*_) = 1 for all τ. By considering the distribution of the random effects as a negative penalty, the h-likelihood estimator [17, 18] can be viewed as a penalized-likelihood estimator.

Lee and Oh [12, 19] proposed an algorithm to obtain an estimator of the regression coefficients of the model using the h-likelihood [17, 18]. They showed that for fixed τ, the *j-*th term of the penalty function *p*_*λ*_(*β*) can be written

$$\frac{\phi }{2\sigma }\frac{\beta_{j}^{2}}{\hat{u_{J}}}+\frac{\phi (\tau -2)}{2\tau }log\hat{u_{J}}+\frac{\phi }{\tau }\hat{u_{J}},$$ (1)

where $\hat{u_{J}}\equiv \hat{u_{J}}(\beta )=\frac{1}{4}\left[{\left\{\frac{8\tau \beta_{j}^{2}}{\sigma }+{\left(2-\tau \right)}^{2}\right\}}^{1/2}+(2-\tau )\right]$. Then, *β* can be estimated by controlling τ and *λ* = *ϕ*/σ. The reparametrization *λ* = *ϕ*/*σ* is valid because the estimation of *β* only depends on the ratio of both parameters *ϕ* and σ. The random effect *u*_*j*_ for the *j-*th parameter *β*_*j*_ imposes a penalty for *β*_*j*_ and is substituted by $\hat{u_{J}}$. This emphasizes the fact that *β* is an unknown fixed parameter and is constrained by the random effect *u*.

**Fig 1:**
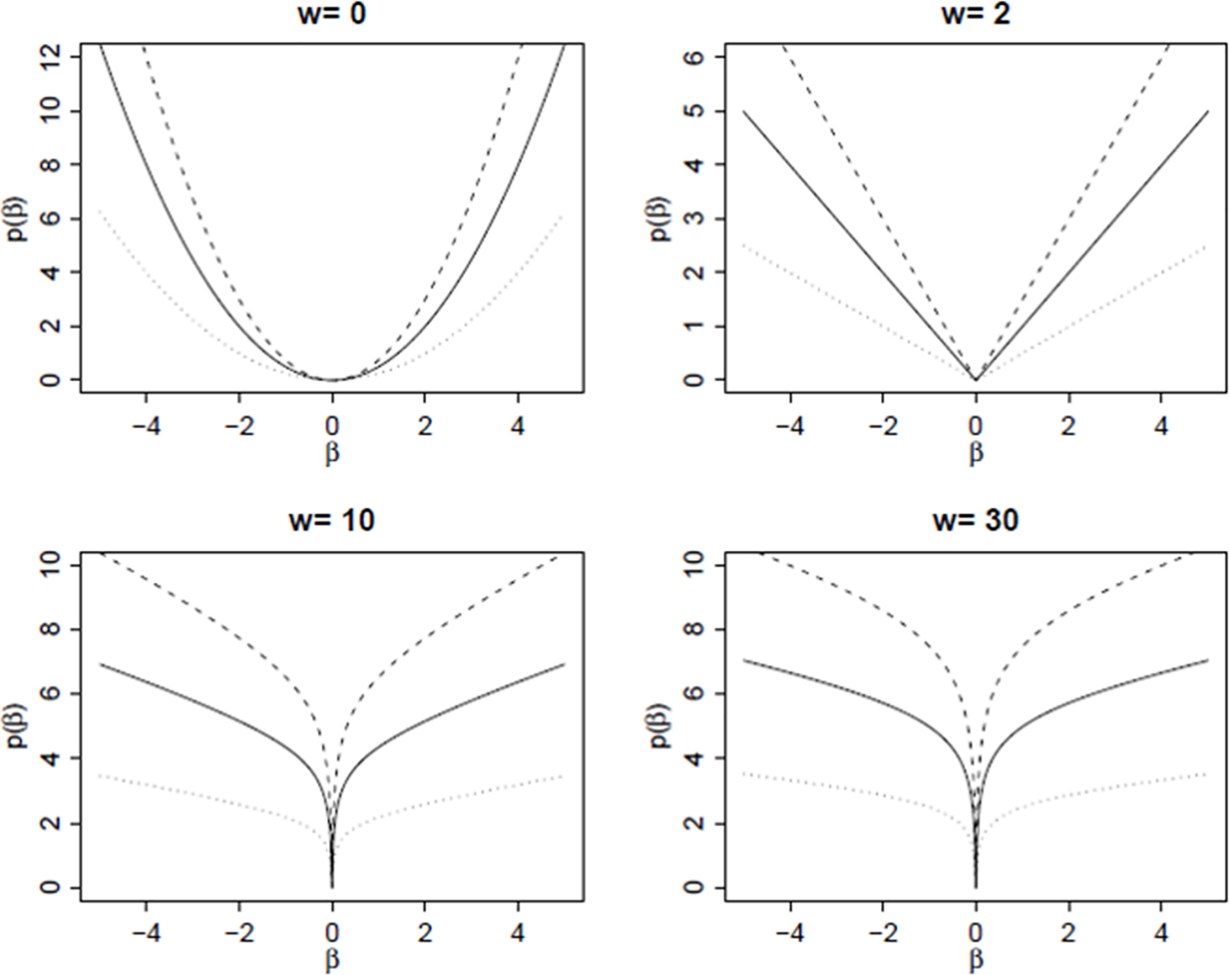
Penalty function *p*_*λ*_(*β*) at different values of w, for λ = 0.5 (dotted line), λ = 1 (solid line) and λ = 1.5 (dashed line). In general, larger values of **λ** are associated with larger penalties, hence more shrinkage and sparsity [18].

This random effect model leads to a family of various penalty functions *p*_*λ*_(*β*) indexed by τ and *λ*. For example, when τ = 0, the penalty function *p*_*λ*_(*β*) becomes $\frac{\phi }{2\sigma }\sum_{j=1}^{p}\beta_{j}^{2}$ which is the one of the ridge regression. Similarly, when τ = 2, the penalty function $p_{\lambda }(\beta )=\frac{\phi }{\sqrt{\sigma }}\sum_{j=1}^{p}\left|\beta_{j}\right|$ is the same as the one of the LASSO regression. By definition the modified unbounded penalty (MUB) corresponds to the case where τ > 2. A graphical representation of the penalty function *p*_λ_(∙) is given on Fig 1 (extracted from [18]) for τ = 0,2,10 and 30 with *λ* = 0.5 (dotted line), *λ* = 1 (solid line), and 1.5 (dashed line). It appears that as the curve becomes more concave near the origin, the sparsity of local solutions increases, and as the slope becomes flat, the amount of shrinkage lessens. The resulting penalty allows thus to control the amount of sparsity and shrinkage by choosing the values of τ and λ. The ridge and LASSO regressions include a bounded penalty and produce shrinkage estimation. Moreover the LASSO has a discontinuity of its derivative at zero which leads to a sparse selection, while the ridge penalty is differentiable at zero and therefore does not offer the possibility to select covariates. The Elastic Net is based on both the ridge and the LASSO penalties. All these methods, including Elastic Net, can however suffer from a limited sparsity in the sense that they can still select too many covariates. With the MUB, the penalty function is not differentiable at zero when τ ≥ 2 and becomes unbounded when τ > 2. The MUB therefore controls both the amount of sparsity and shrinkage and offers a sparse estimation of the regression parameters without losing prediction accuracy [12, 13].

**Fig 2:**
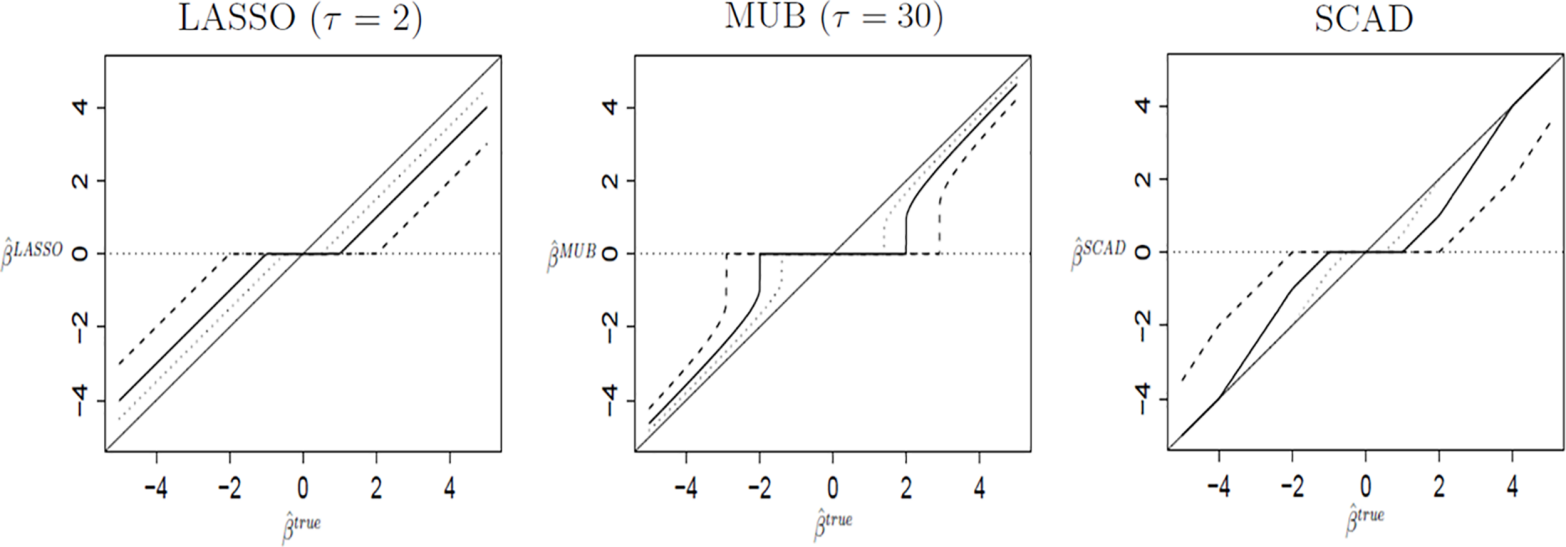
Relationship between the coefficients from the true model (x-axis) and those of a sparse regression model (y-axis) obtained respectively with the LASSO, the MUB and the SCAD penalties for λ = 0.5 (dotted line), λ = 1 (solid line) and λ = 2 (dashed line) [18].

On Fig 2 is plotted the relationship between the ordinary least squares estimator (x-axis) and corresponding penalized likelihood estimator (y-axis) obtained respectively with the LASSO, the MUB and the SCAD penalties (solid line indicates λ = 1, dashed line λ = 2 and dotted line λ = 0.5; for more details, see Ch.11 in [18]). It can be observed that (1): all three methods produce shrinkage estimators compared to ordinary least squares estimator; (2): the higher the value of the estimators of the MUB and the SCAD, the closer they get to the ordinary least squares estimator. This oracle property cannot be satisfied for the LASSO; (3): the SCAD becomes the ordinary least square estimator for higher value, whereas the MUB shrinks the coefficients.

Sparse covariate selection is beneficial for the interpretation of the model, while shrinkage estimation is beneficial for prediction accuracy. We therefore chose to apply both the LASSO and the MUB penalizations to our datasets in order to select predictive genes of response to chemotherapy in breast cancer and to compare their performances.

Note that Bayesian methods like spike and slab, and the Bayesian LASSO have also been developed for variable selection. They achieve the sparsity through the use of the specific prior distribution of *β*, whereas the MUB controls the sparsity via the use of specific distribution of *u* [9–11].

### 2.2 Breast cancer chemotherapy datasets

Our analysis was based on four curated gene expression datasets which have been described previously in details by de Ronde et al [15]. The names of the patients and of the genes had been anonymised and the patients had signed informed consent. In each dataset the expression of 12010 genes was measured by microarray in female patients with breast cancer treated by chemotherapy. Moreover, the pathological complete response (pCR) of these patients to treatment was evaluated by biopsy. Each of the datasets corresponds to a specific clinical subtype of breast cancer, as explained below.

Breast cancer is indeed a very heterogeneous pathology and response to treatment depends on the clinical subtype of the disease. As explained in the original study of de Ronde et al [15], the binary hormonal receptor status of each patient (oestrogen receptor (ER+ or ER-), progesterone receptor (PR+ or PR-) and human epidermal growth factor receptor 2 (HER2+ or HER2-)) allows defining five different breast cancer subtypes: the HER2+ subtype, the luminal subtype (HER2-, ER+), the triple negative subtype (HER2-, ER-, PR-), the (HER2+, ER-) subtype and the (HER2+, ER+) subtype.

In our pooled database, 394 patients were included in the study among which 87 (22%) were classified as having no residual cancer after treatment whereas 307 (78%) were classified as not responding to the treatment. The number of patients of each clinical subtype is summarized in Table 1 and categorized by response to chemotherapy (adapted from de Ronde et al [15]). Note that the sample size of the (HER2+, ER+) subtype was not sufficiently large to be clinically conclusive and was therefore not investigated neither in our study nor in the seminal one [15].

**Table 1:** Patient disposition by breast cancer clinical subtype and response to chemotherapy. pCR: pathological complete response.

Dataset	pCR—n(%)	No pCR—n(%)
HER2+	31 (38)	51 (62)
Luminal	14 (7)	185 (93)
Triple Negative	40 (38)	64 (62)
HER2 +, ER -	25 (56)	20 (44)
pooled database	87 (22)	307 (78)

### 2.3 Statistical analysis

Overall, our database consisted in 12010 continuous covariates measured in 394 individuals in order to predict their response to chemotherapy, which is a binary response variable. For a binary response variable, the following logit model is considered:

$$Y_{i}∼Ber(p_{i}),\;{logit(p}_{i})=x_{i,1}\beta_{1}+\cdots x_{i,p}\beta_{p},$$

where for 1 ≤ *i* ≤ *n*, *Y*_*i*_ is the response variable, *x*_*i*_ = (*x*_*i*,1_,…,*x*_*i*,*p*_) is the vector of the covariates, *β*_1_,…,*β*_*p*_ are the regression parameters. The penalized likelihood function, namely the h-likelihood, is given by

$$\sum_{i=1}^{n}\left\{Y_{i}(\sum_{j=1}^{p}x_{i,j}\beta_{j})-log\;(1+e^{\sum_{j=1}^{p}x_{i,j}\beta_{j}})\right\}-p_{\lambda }(\beta )$$ (2)

And the beta coefficients are then obtained by maximizing (2).

In total five analyses were performed: one for each of the four clinical subtypes and one for the pooled database made of the four pooled subtypes.

Our objective was to compare the number of genes selected and the predictive accuracy of LASSO and MUB regressions in predicting response to chemotherapy in each of the four subtypes and in the pooled database. To do so, the same analysis was applied to each dataset:

First, optimal values of the tuning parameters of each method were selected among a pre-set grid of candidate values by 3-fold cross-validation.

In detail, we searched 15 grid points equally spaced between 2 and 30 for *τ*, and 30 grid points between 0 and 1 for *σ*, respectively [13] and found (*τ*,*σ*) which maximize the penalized likelihood function (2).

More precisely each dataset was randomly divided in 3 subsets of the same size. Two out of the 3 subsets were selected and pooled in order to estimate all the regression coefficients of the LASSO and MUB. A classifier of response to chemotherapy was then built up with the linear predictor including the covariates having received an estimated nonzero coefficient. The third subset was used as a test sample on which the likelihood value of the classifier was evaluated. Each of the three subsets was sequentially used as a test sample whereas the remaining two other subsets were pooled together as training sample. The values of the tuning parameters that led to the selection of the set of covariates maximizing the likelihood value of the model were finally retained as the optimal ones.

Second, once the optimal values were selected, these were attributed by default to the tuning parameters of LASSO and MUB regressions which were then re-fitted using the whole dataset. For each method, the linear predictor was built including the covariates having received a nonzero estimated regression coefficient and its predictive accuracy was evaluated by plotting its ROC curve and computing the corresponding AUC by reclassification (i.e. using the same original dataset as both training and test sample). The difference between the AUC obtained with LASSO and MUB was tested for nullity using DeLong’s paired test [20]. Finally, in order to correct for the optimism caused by the use of the same dataset for both the estimation of the model parameters and the evaluation of the predictive accuracy of the classifiers by the AUC, different internal validation techniques were applied and compared. First, the same double-loop cross validation procedure as the one described by de Ronde et al [15], where the data were originally analysed, was implemented. Second, three variants of bootstrap internal validation techniques were used: the regular bootstrap, the bootstrap .632 and the bootstrap .632+. These methods are described in details in Steyerberg et al [21] and Collignon [22]. All three methods use bootstrap samples of the original dataset (i.e. random samples with replacement of the actual dataset with the same size) as training samples to fit the LASSO and MUB regressions and select the most discriminant covariates. The original dataset is then used as a test sample. For bootstrap .632 and .632+ variants the individuals randomly selected in the current bootstrap training sample are first removed from the test sample before computing the AUC. For the regular bootstrap, the difference between the AUC obtained by reclassification of the bootstrap training sample and the AUC obtained by classifying the test sample was averaged over 100 bootstrap replications in order to estimate the optimism of the method. The corrected AUC was then obtained by subtracting the average optimism from the AUC originally computed by reclassification. For the .632 and .632+ bootstrap variants, the corrected AUC was a weighted average of the AUC obtained in reclassification and of the average of the 100 different AUC obtained on the test sample (the weighting of the .632+ variant favours the performance obtained on the test sample in case of overfitting). In order to evaluate the robustness of the covariate selection, the mean and standard deviation of the number of covariates selected was computed over the 100 bootstrap replications of the LASSO and MUB. Moreover, the percentage of selection of each covariate over the 100 bootstrap replications was also computed.

Although the AUC is the most widely accepted measure of models predictive performances [1, 3], other criteria can be of interest. For example when the sample sizes of the two groups to discriminate are markedly unbalanced, the AUCPR can also be informative [16]. In our case the imbalance between the “pCR” and “no pCR” groups was not extreme but we also computed as a sensitivity analysis the AUCPR of the LASSO and the MUB. Note that the variables used in these classifiers are the same as for the ROC curves since the beta coefficients are estimated by maximizing the the h-likelihood.

### 2.4 Comparison with the original study

In their original study, de Ronde et al [15] combined different covariate selection techniques to several classifiers in order to build the best predictive model of response to chemotherapy. Performances were corrected for optimism by double-loop cross validation. Among these methods, the model maximizing the corrected AUC was retained as the best one. In order to compare its performances to ours, we retrieved the mean number of genes selected during double-loop cross validation as well as the corresponding corrected AUC for the best model developed by de Ronde et al [15] (see also Supplementary Information of their paper (http://journals.plos.org/plosone/article?id=10.1371/journal.pone.0088551#s6).) Note no results about bootstrap internal validation was available in the original study.

## 3. Results

In order to illustrate our analysis with the largest sample size possible, the results obtained with the pooled database are described in details before summarizing those achieved in each of the four clinical subtypes.

### 3.1 Pooled database

**Fig 3:**
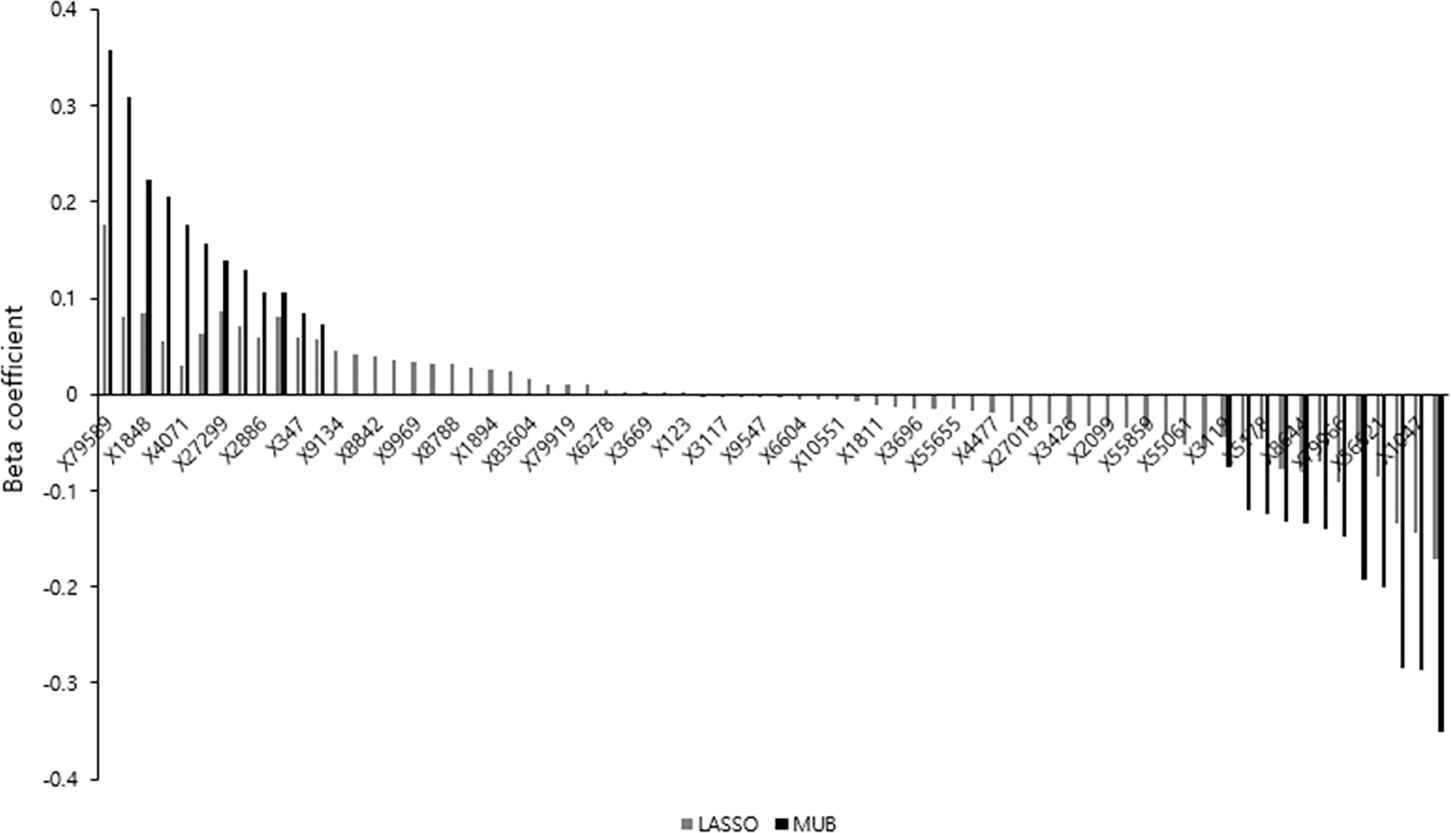
Pooled database—Bargraph representing on the y-axis the regression coefficients of the covariates selected by at least one of the covariate selection method in the pooled database. The genes on the x-axis are sorted by decreasing order of their LASSO regression coefficients and are labelled as anonymised in the database.

In the pooled database, 24 covariates were selected using the MUB penalization whereas 70 were selected with the LASSO. In Fig 3 are depicted on the y-axis the regression coefficients of the covariates selected by at least one of the methods. It appears that the covariates selected by the MUB are actually a subset of those obtained by the LASSO. The corresponding models are therefore nested. For the covariates retained by both methods, the estimated MUB regression coefficients were greater in absolute value than the LASSO ones. This finding confirms that the MUB controls the amount of shrinkage, as discussed in Section 2.1. It is interesting to emphasize here the inadequacy of the likelihood ratio test (LRT) to compare the model fits. Indeed, based on the idea that adding supplementary variables to a model should increase its fit, the LRT is a tool to assess if the corresponding increase in likelihood is significant, by adjusting for the number of covariates used in both models, which are nested. However, the MUB model is based on a smaller number of parameters and yet gives a larger likelihood than the LASSO. It is because the LASSO could shrink the coefficients excessively. Comparing the model fits with the LRT would lead to a non-significant p-value whereas the MUB clearly outperformed the LASSO while requiring less parameters.

**Fig 4:**
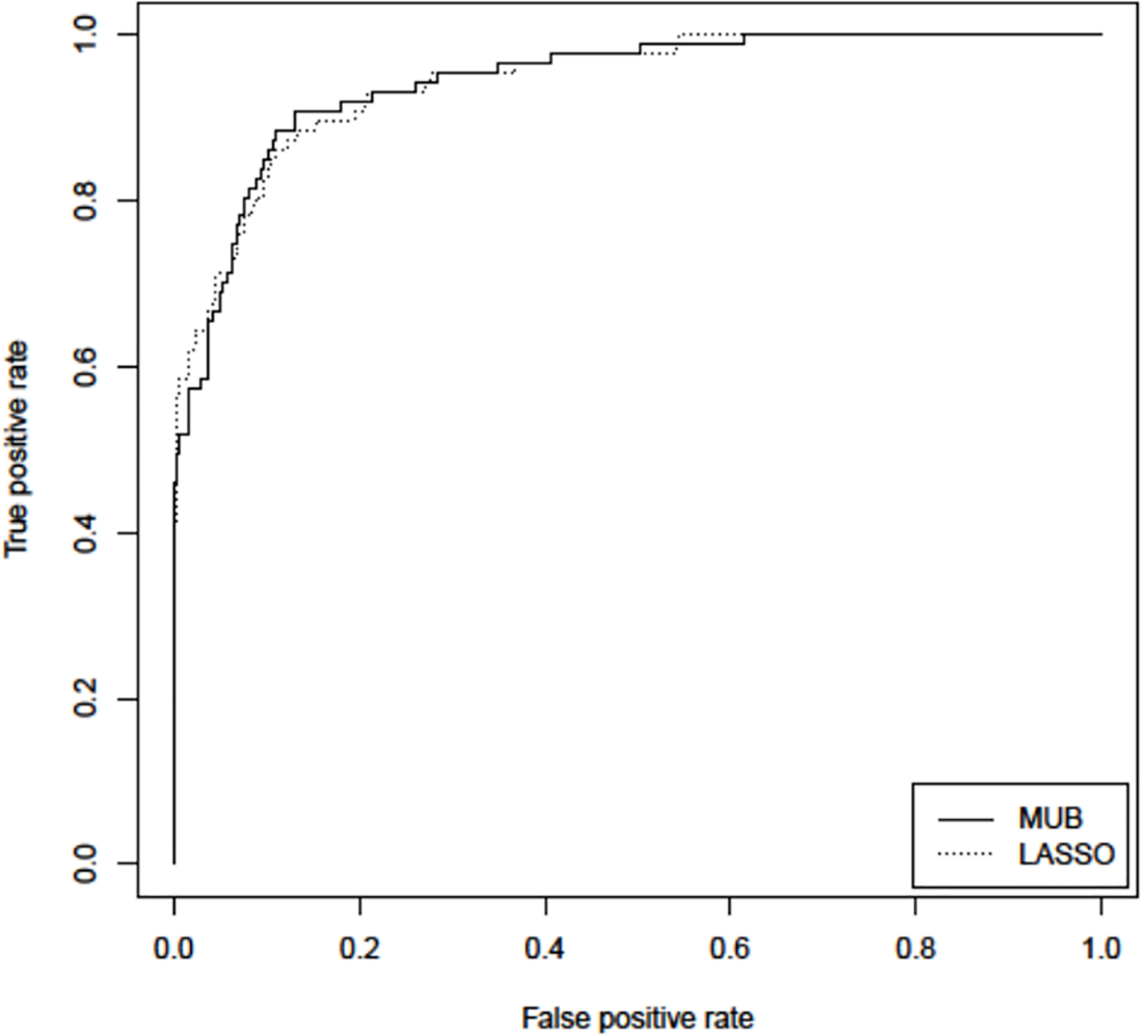
Pooled database—ROC curves of the linear predictors built with the selected covariates of each method. MUB: solid line; LASSO dotted line.

On Fig 4 are depicted the two ROC curves of the linear predictors built with the selected covariates of each method. It appears that both curves are almost superimposed, leading to a similar predictive performance with a rounded AUC of 94% (p = 0.94, De Long test). The MUB reached thus the same performance as the LASSO with about one third of the number of covariates selected.

**Fig 5:**
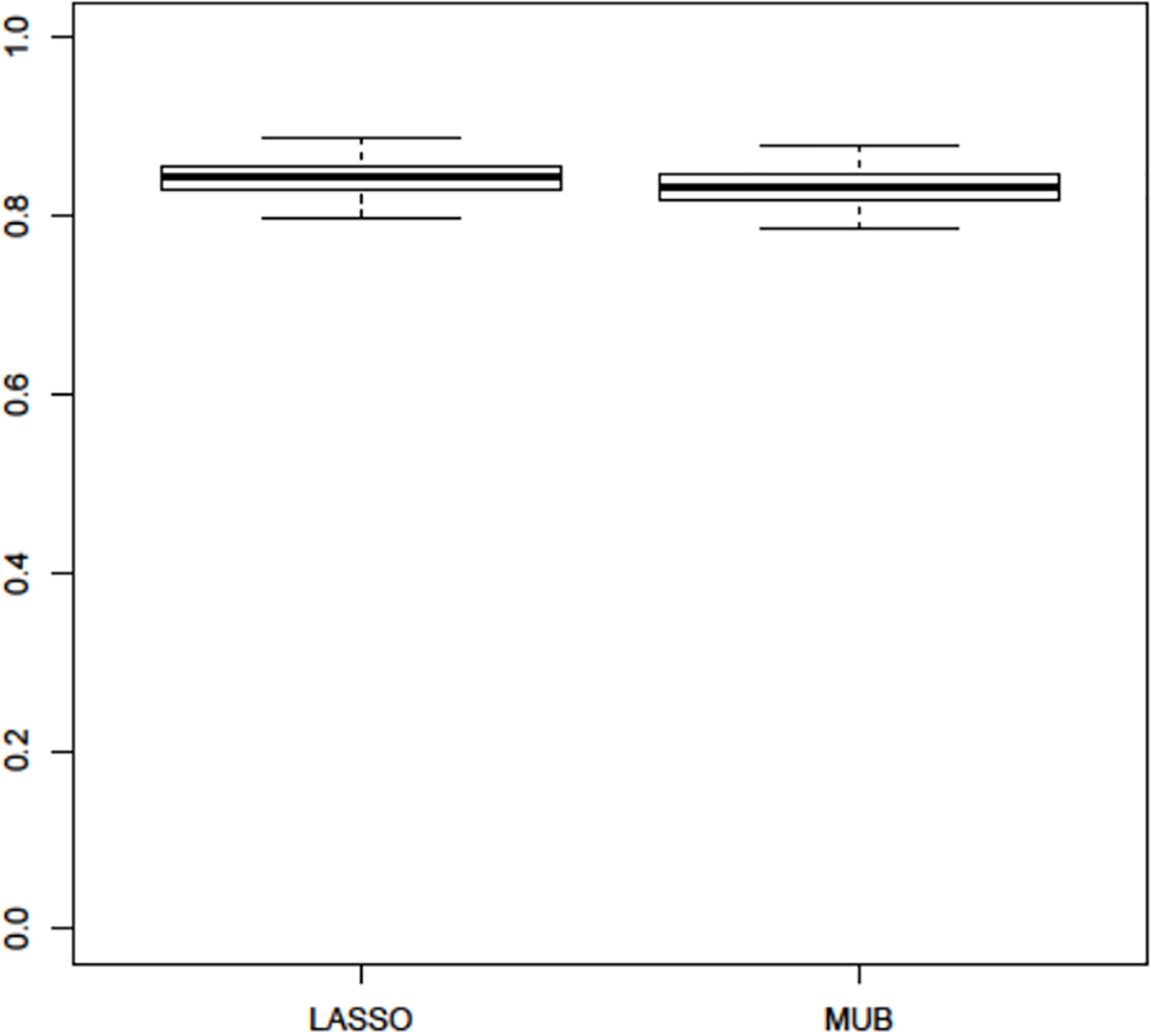
Pooled database—boxplot of the AUC corrected by the regular bootstrap internal validation technique using the optimism calculated within each bootstrap training sample.

**Fig 6:**
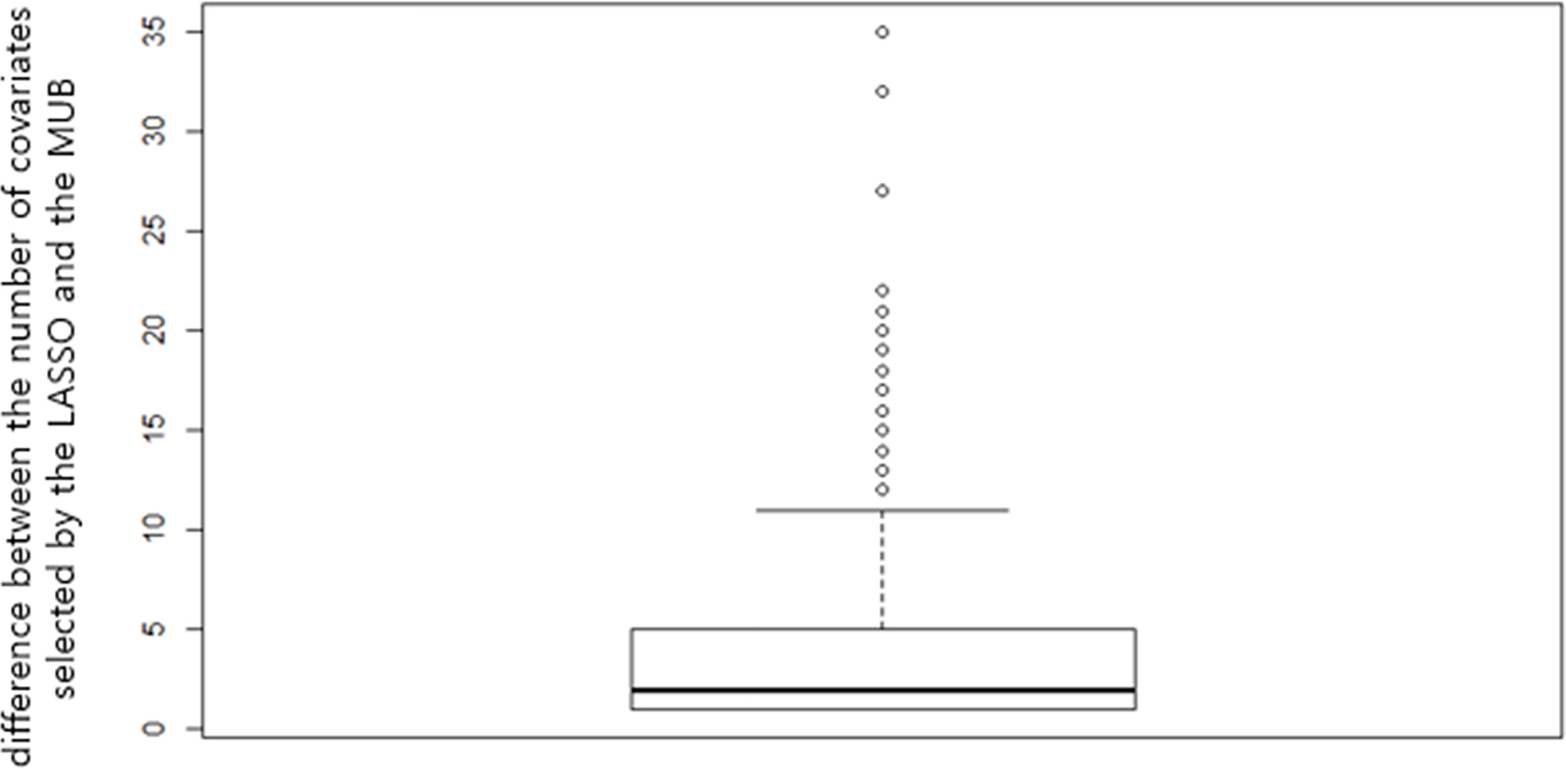
Pooled database—boxplot of the difference between the number of covariates selected by the LASSO and the MUB over the 100 bootstrap training samples.

**Fig 7:**
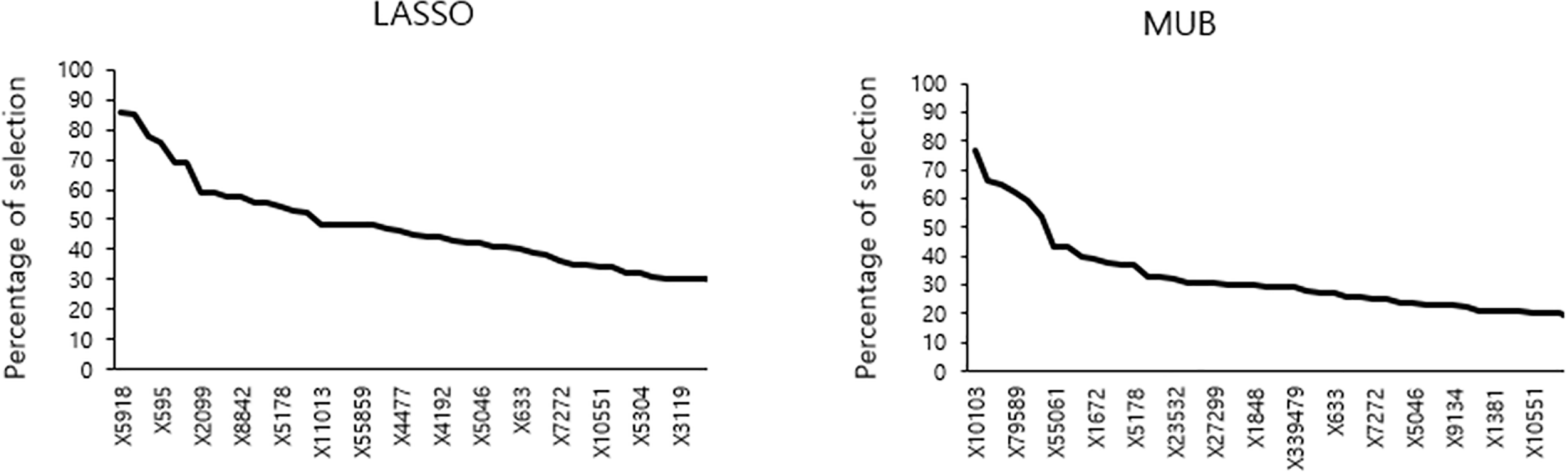
Pooled database—percentage of time each gene was selected during the bootstrap replications by each covariate selection method. Only the 15 most selected genes are represented.

Since the training and test samples were the same, the two AUC are overoptimistic and had thus to be corrected for overfitting. Using the regular bootstrap internal validation technique, the results obtained with both methods remained very comparable since the corrected AUC was 83% for the MUB and 84% for the LASSO. The boxplot of the AUC corrected for the optimism calculated with each bootstrap training sample is displayed on Fig 5 and shows that over the 100 replications the results remained very similar, although the LASSO appeared however to be slightly superior to the MUB in terms of predictive accuracy. The same observations were made when using different validation techniques: 80% vs 82% with the bootstrap .632, 78% vs 80% with the bootstrap .632+ and 75% vs 76% by double-loop cross-validation for the MUB and the LASSO respectively (corresponding boxplots are shown in Figures A, B and C in S1 File). However, it has to be emphasized that over the 100 bootstrap replications, the mean (standard deviation) number of covariates selected with the MUB was remarkably smaller than the LASSO’s: 32.87 (2.48) vs 51.27 (3.48). The boxplot of the difference between the number of covariates selected by the LASSO and the MUB over the 100 bootstrap training samples is depicted on Fig 6, and it appears clearly that the MUB penalization always selected less covariates than the LASSO. On Fig 7 is represented the percentage of time each gene was selected during the bootstrap replications by each covariate selection method. Only the 15 most selected genes are represented. The slopes of the curves appeared to be rather similar, although the ranking of the genes (by decreasing percentage of selection) was markedly different.

**Fig 8:**
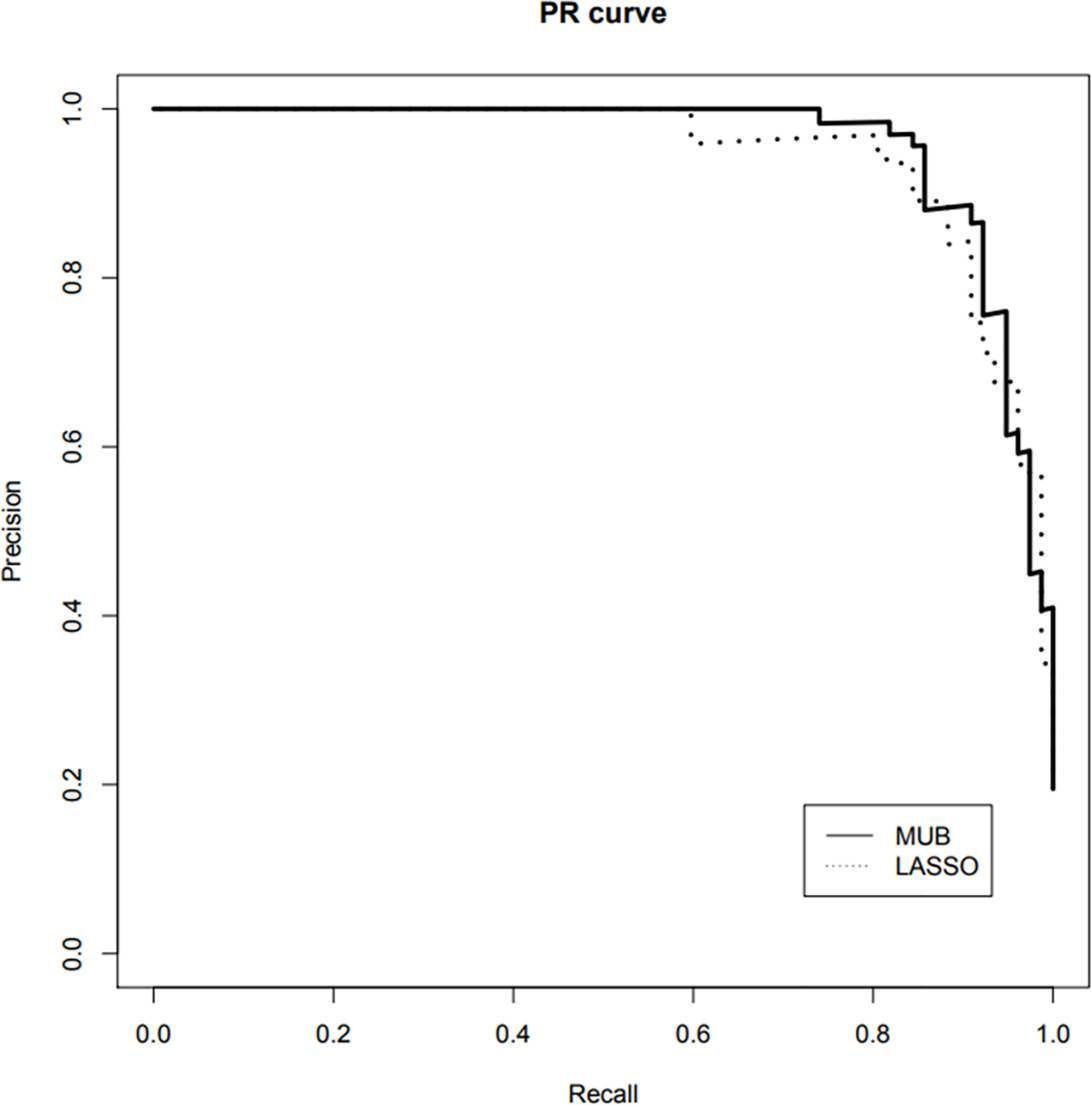
Pooled database–PR curves of the linear predictors built with the selected covariates of each method. MUB: solid line; LASSO dotted line.

On Fig 8 are plotted the respective PR curves of the LASSO and the MUB applied to the pooled dataset. It can be seen that the curves are very close to each other and again the respective AUCPR show no marked difference (LASSO: 95%, MUB: 96% in reclassification, LASSO: 43%, MUB: 41% by double-loop cross-validation). The boxplots of the AUCPR corrected by the double loop CV procedure are shown in Figure D in S1 File. It is moreover important to emphasize that the variables used to build these classifiers are the same as for the ROC curves. Indeed these variables are selected by maximisation of the h-likelihood, which is a criterion independent of the measure of predictive performance chosen.

### 3.2 Results by clinical subtypes

An overview of the results obtained with both covariate selection methods in each dataset is summarized in Table 2 (See Figures E to AJ in S1 File).

**Table 2:** Overview of the results obtained with both covariate selection methods in each dataset. n: sample size; sd: standard deviation; AUC: Area Under the Curve; CV: cross-validation.

	LASSO								MUB							
dataset	n	number of selected covariates	mean number of covariates selected during validation (sd)	AUC	AUC regular bootstrap	AUC .632	AUC .632+	AUC double-loop CV	number of covariates selected	mean number of covariates selected during validation (sd)	AUC	AUC regular bootstrap	AUC .632	AUC .632+	AUC double-loop CV	De Longs' test p-value
All	394	70	51.27 (3.48)	94%	84%	82%	80%	76%	24	32.87 (2.48)	94%	83%	80%	78%	75%	0.9411
Her2 +	82	14	37.54 (2.62)	88%	90%	78%	71%	66%	2	15.02 (1.49)	83%	88%	78%	71%	63%	0.0977
Luminal	199	30	43.91 (7.82)	100%	87%	77%	69%	62%	25	3.22 (1.07)	100%	85%	77%	69%	61%	0.1864
Triple -	104	7	47.76 (3.03)	80%	89%	75%	65%	58%	4	12.99 (1.29)	83%	86%	75%	65%	57%	0.325
Her2+ ER-	45	28	23.97 (2.02)	100%	89%	75%	65%	59%	3	10.55 (1.21)	92%	85%	76%	66%	54%	0.0917

Overall the results elicited on the pooled database were also observed in each of the clinical subtypes. Indeed in each of them, the MUB always selected less covariates than the LASSO, as for example 2 vs 14 for the Her2+ subtype. The covariates selected by the MUB were always a subset of those selected by the LASSO and the models were therefore nested. De Long’s test to compare the AUC of the classifiers in reclassification was never significant. Predictive performances tended to vary marginally with the validation method. They were in general slightly superior for the LASSO when correcting the AUC by the regular bootstrap internal validation technique or the double-loop cross-validation, whereas they were almost identical and even sometimes slightly superior for the MUB when correcting it with the bootstrap .632 or 632+ variants. The initial amount of overfitting was large in the luminal and Her2+ ER- subtypes where almost perfect AUC (i.e. equalling to 100%) were observed before correction, which could be attributed to the small group sample size of these subtypes. Interestingly the same pattern was always observed in all clinical subtypes for both covariate selection methods, in which the AUC were systematically ranked in the following decreasing order: regular bootstrap internal validation, bootstrap 632, bootstrap 632+, double-loop cross-validation.

The regression coefficients were also less shrunken with the LASSO than the MUB in the Her2+ and triple negative subtypes while the converse tended to be observed in the luminal and Her2+ ER- subtypes (See Figures E, M, U and AC in S1 File). It has however to be pointed out that the luminal subtype contained a very small proportion of patients responding to chemotherapy (7%) while the Her2+ ER- subtype had a very low sample size. These results have therefore to be treated with caution.

Surprisingly, using the MUB penalization the number of covariates selected in the actual luminal subtype was much larger than the mean number of covariates selected during bootstrap internal validation (25 vs 3.22). The converse was observed with the LASSO in the triple negative subtype, were a very small number of covariates was selected as compared to the mean number of covariates selected during bootstrap internal validation (7 vs 47.76).

### 3.3 Comparison with the original study

The mean number of covariates selected during double loop CV by the best model of de Ronde et al [15] and the corresponding corrected AUC are reported in Table 3 and compared to our own results. Despite the fact that no results on bootstrap internal validation was available for the study of de Ronde et al, it is hoped that this would give an order of magnitude and would help inform the comparison.

**Table 3:** Mean number of covariates selected and AUC corrected by double loop cross-validation for the LASSO, the MUB and the best model of de Ronde et al [15].

	LASSO		MUB		Best model from de Ronde et al [15]	
dataset	mean number of covariates selected during bootstrap internal validation	AUC double-loop CV	mean number of covariates selected during bootstrap internal validation	AUC double-loop CV	mean number of covariates selected during double loop CV	AUC double-loop CV
All	51	76%	33	75%	31	77%
Her2 +	38	66%	15	63%	31	69%
Luminal	44	62%	3	61%	41	61%
Triple Negative	48	58%	13	57%	53	68%
Her2+ ER-	24	59%	11	54%	58	51%

Using double loop CV, the performances published by de Ronde et al [15] were slightly superior for the Her2+ subtype and the pooled database, and were markedly better for the triple negative subtype. However they were outperformed by the LASSO and MUB in luminal and Her2+ER- subtypes. Interestingly, in each clinical subtype the average number of selected covariates was always smaller with the MUB than with the best model of de Ronde et al [15], whereas the LASSO only selected fewer genes in the triple negative and Her2+ER- subtypes. In the pooled database, on average the MUB only selected two more variables than the method of de Ronde et al, while the LASSO selected twenty more.

## 4. Conclusion

In this study, we found consistently in several different datasets that the MUB penalization tended to select a remarkably smaller number of covariates than the LASSO’s while offering similar predictive accuracy (the models obtained were actually nested). Indeed the difference between the performances of the classifiers built with the covariates selected with each covariate selection method was relatively slight and varied moderately by datasets and validation technique. When comparing the results obtained to those published in the study where the data were originally described and analysed, the predictive accuracy obtained with the LASSO and the MUB varied only moderately from the performance of de Ronde et al [15]. However, we found again that the number of covariates selected appeared to be much smaller. In these high-dimensional datasets, the MUB appeared therefore to be an efficient method to select a small number of important predictive genes of resistance to chemotherapy while offering encouraging predictive performances. Although Bayesian alternatives could be considered, we think that in a high-dimensional setting, the h-likelihood based method is easier to estimate. Thus, when sparsity is required, MUB seemed therefore to be the best option in high-dimension. Investigation in other datasets is to be planned in order to confirm these findings in other high-dimensional datasets.

## Supporting information

S1 File Boxplots of the AUC corrected by the bootstrap .632 and .632+ variants and by double-loop cross-validation on the pooled database as well as all Figures illustrating the results obtained with the specific clinical subtype subtypes. The boxplots of the AUCPR obtained by double-loop cross-validation on the pooled database are also given.
(PPTX)

S1 Code Main R code to run the analysis. (R)

S1 Functions Library of R functions to run the main code. (R)
