# Supplementary material for: Comparison of the modified unbounded penalty and the LASSO to select predictive genes of response to chemotherapy in breast cancer
Source: PLoS One. 2018 Oct 1;13(10):e0204897. doi: 10.1371/journal.pone.0204897 (PMC6166949; doi:10.1371/journal.pone.0204897)
Supplement: S1 File — The boxplots of the AUCPR obtained by double-loop cross-validation on the pooled database are also given. (PPTX) [file pone.0204897.s001.pptx]

## Slide 1
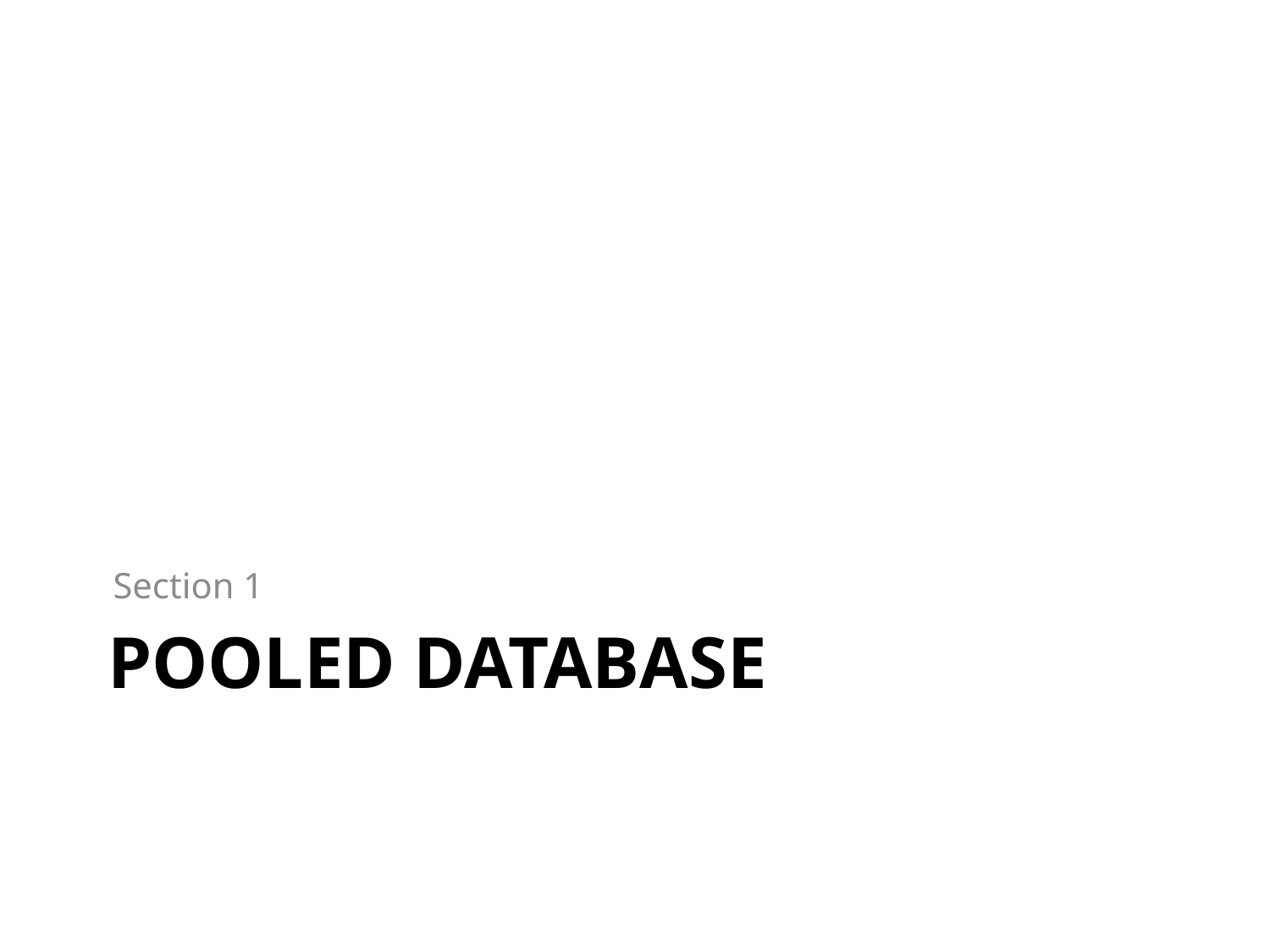

Section 1
# POOLED DATABASE

## Slide 2
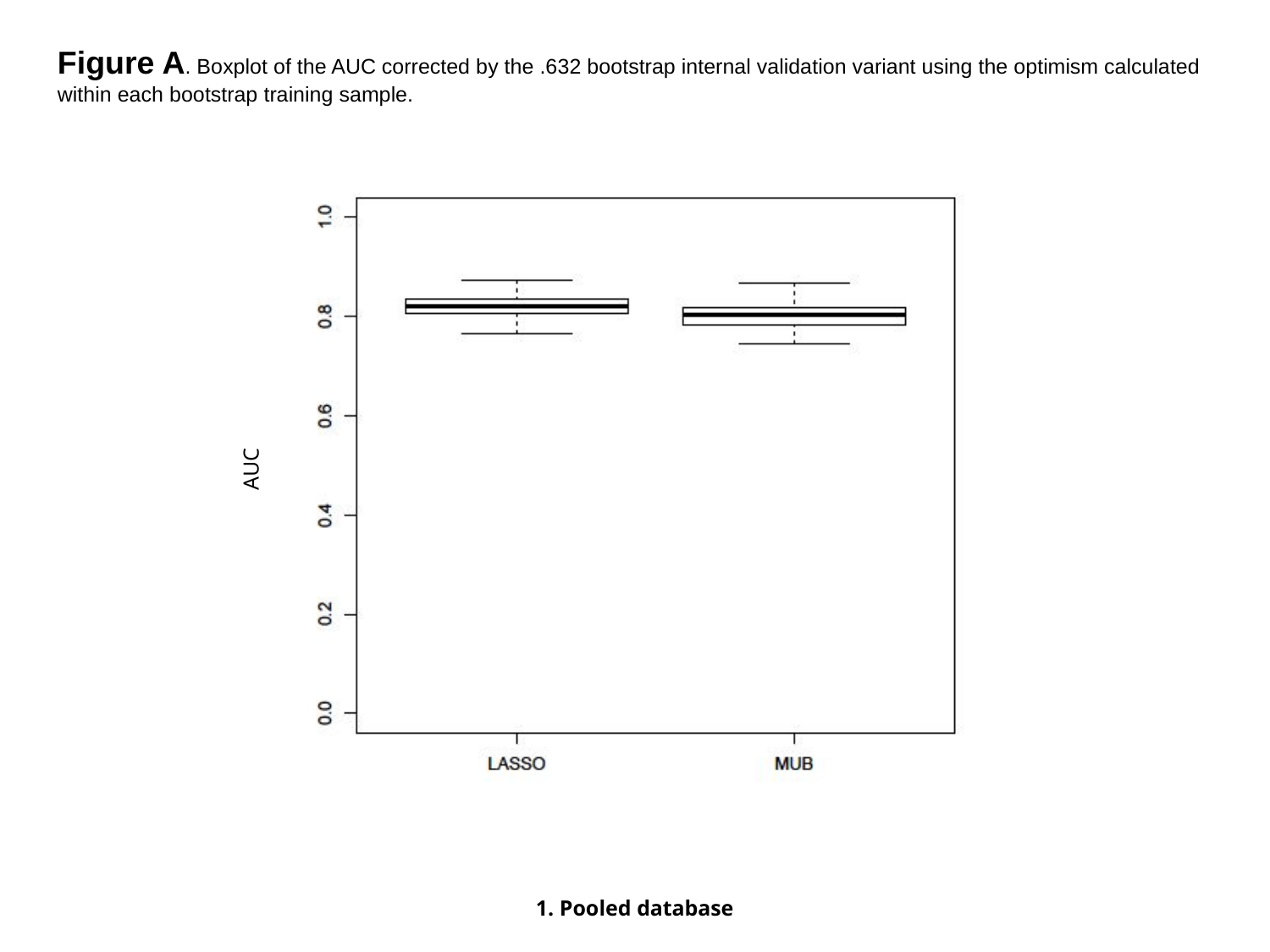

Figure A. Boxplot of the AUC corrected by the .632 bootstrap internal validation variant using the optimism calculated within each bootstrap training sample.
AUC
1. Pooled database

## Slide 3
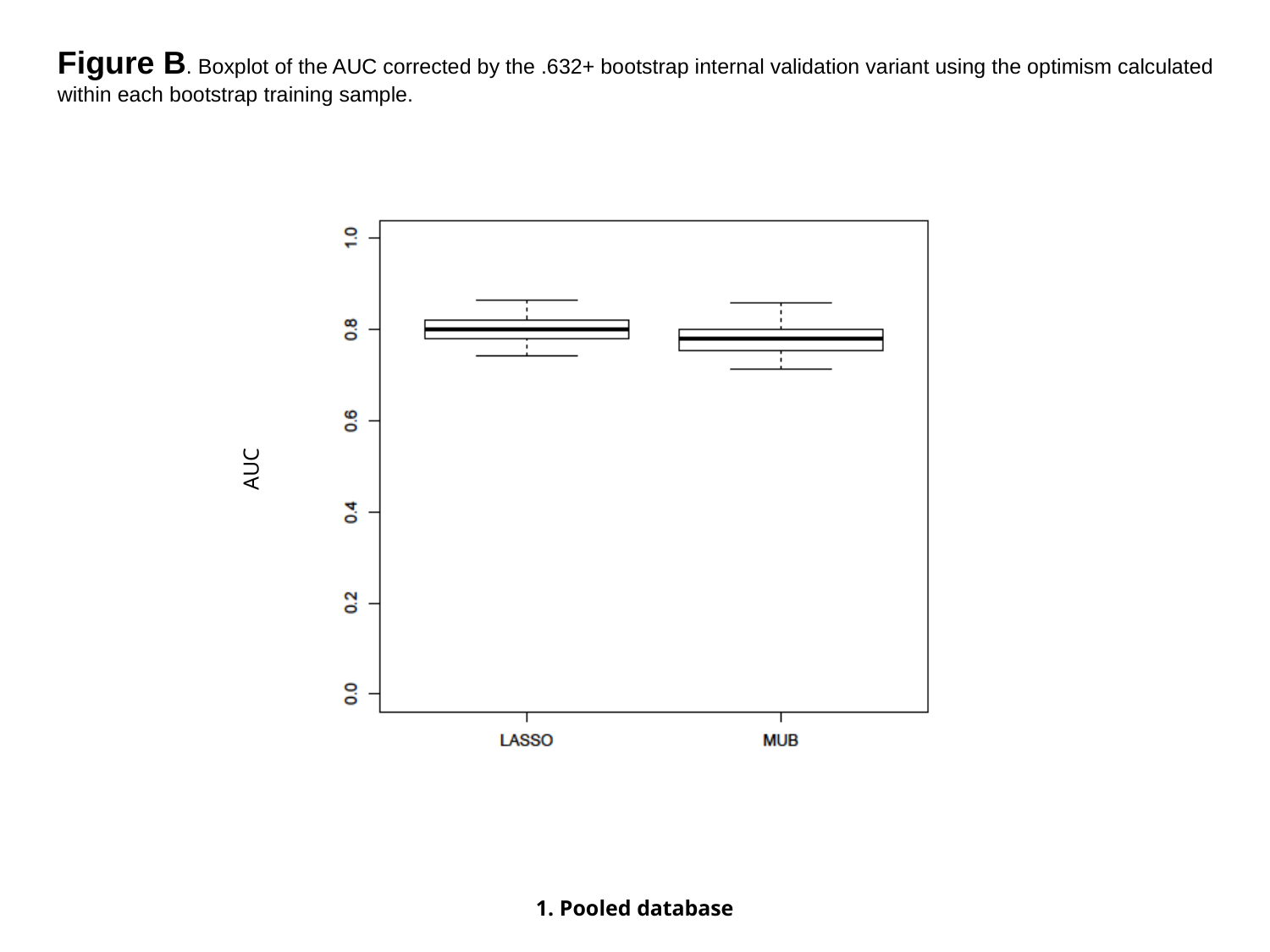

Figure B. Boxplot of the AUC corrected by the .632+ bootstrap internal validation variant using the optimism calculated within each bootstrap training sample.
AUC
1. Pooled database

## Slide 4
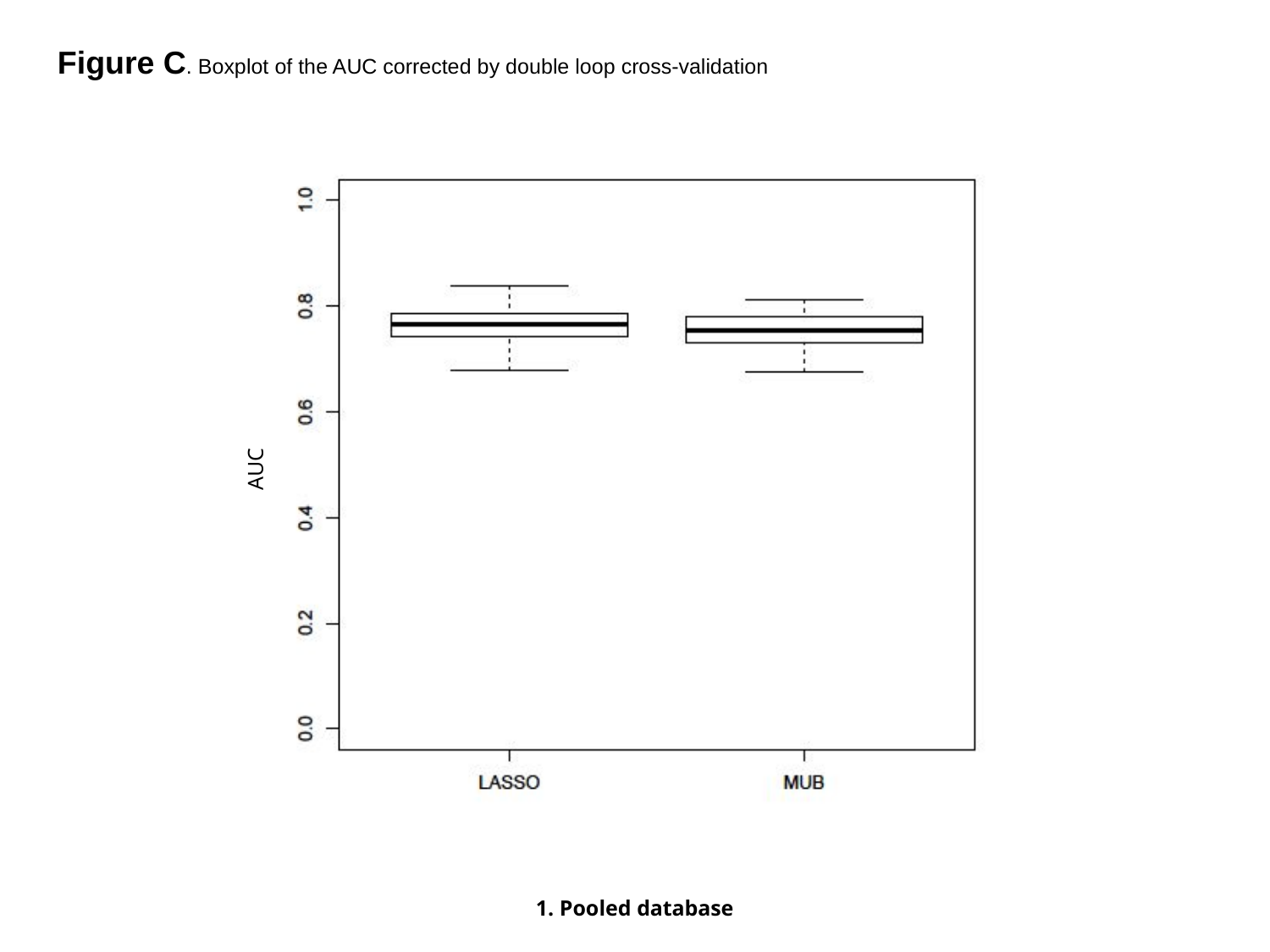

Figure C. Boxplot of the AUC corrected by double loop cross-validation
AUC
1. Pooled database

## Slide 5
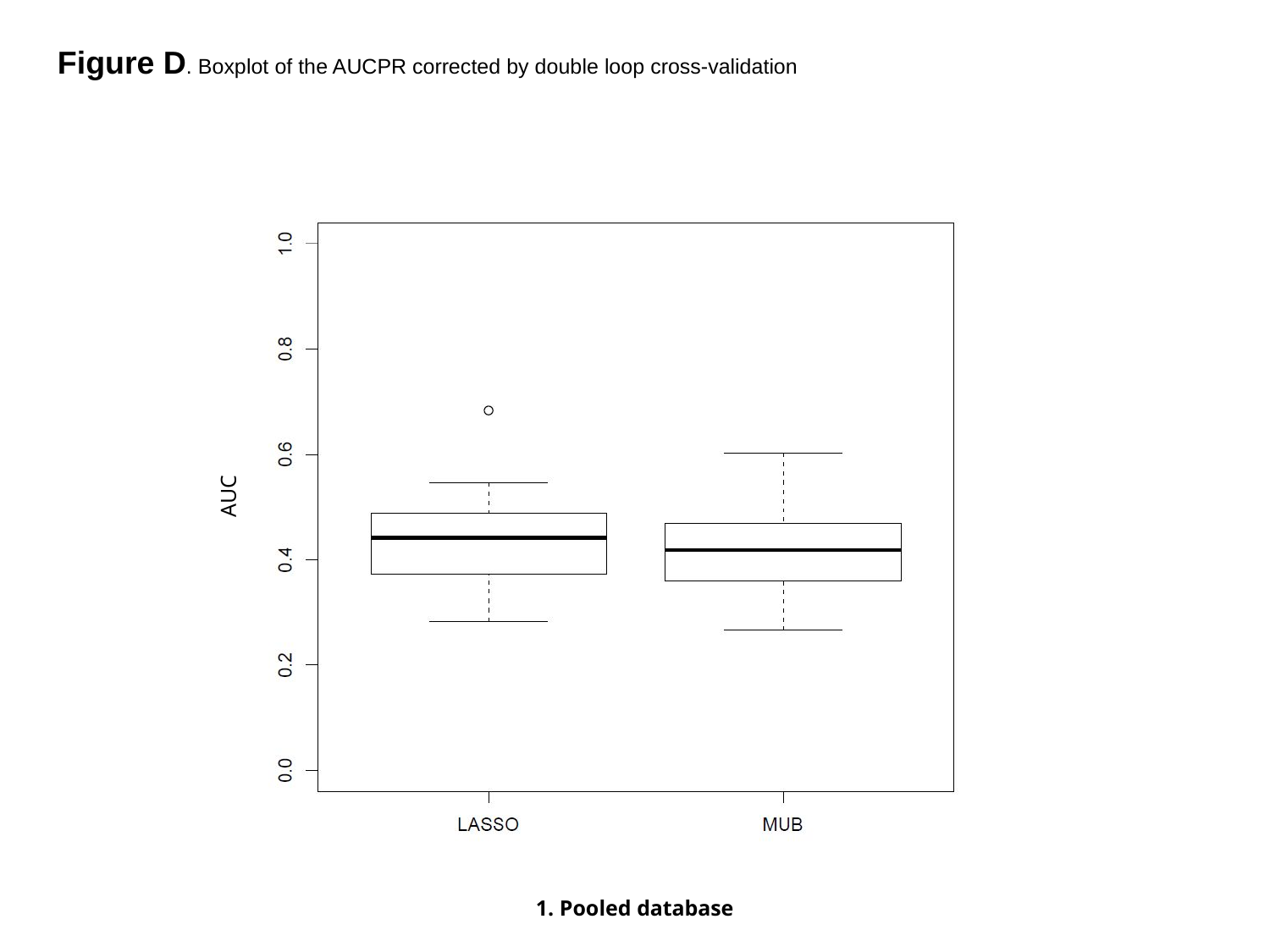

Figure D. Boxplot of the AUCPR corrected by double loop cross-validation
AUC
1. Pooled database

## Slide 6
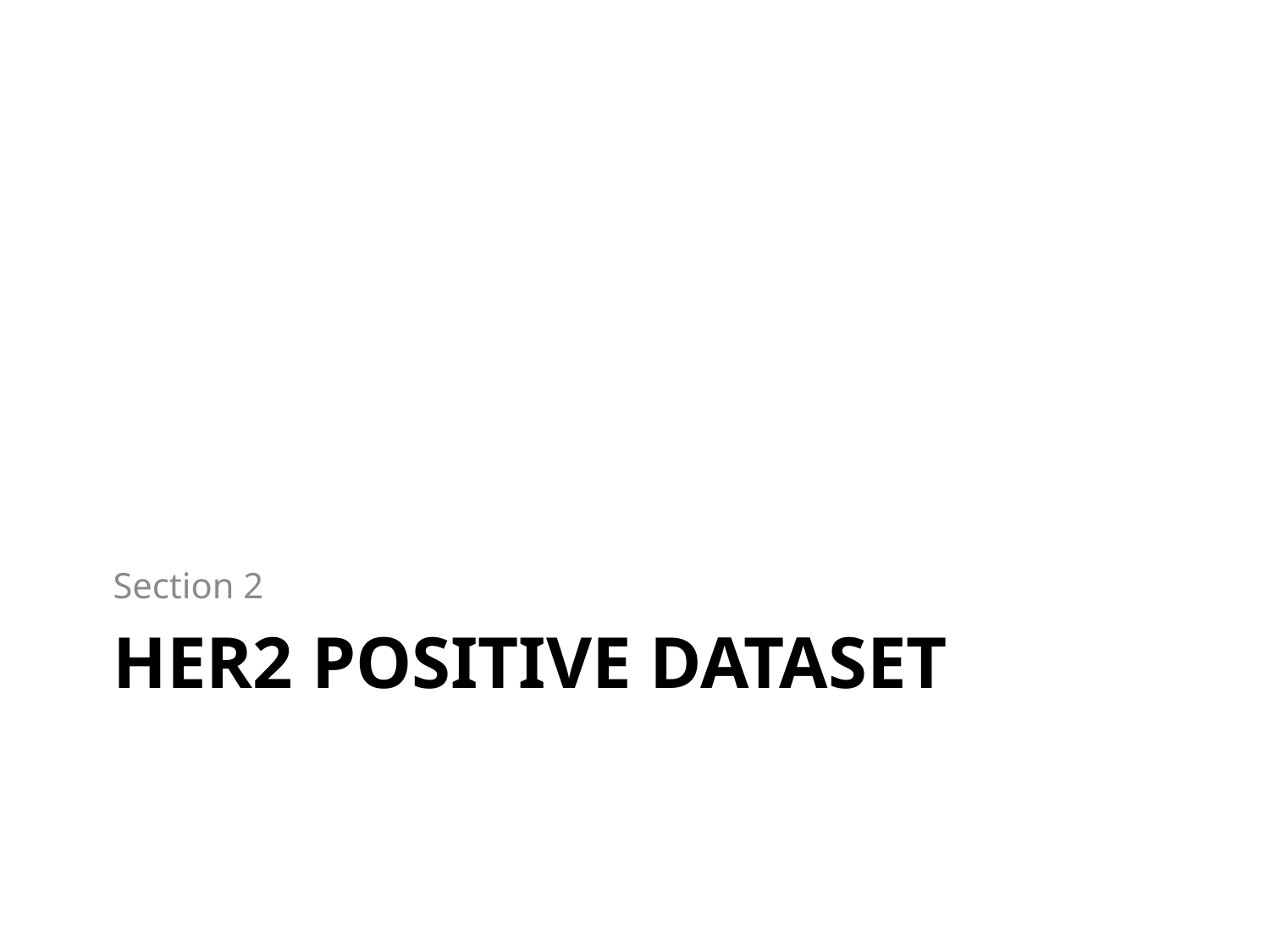

Section 2
# Her2 positive dataset

## Slide 7
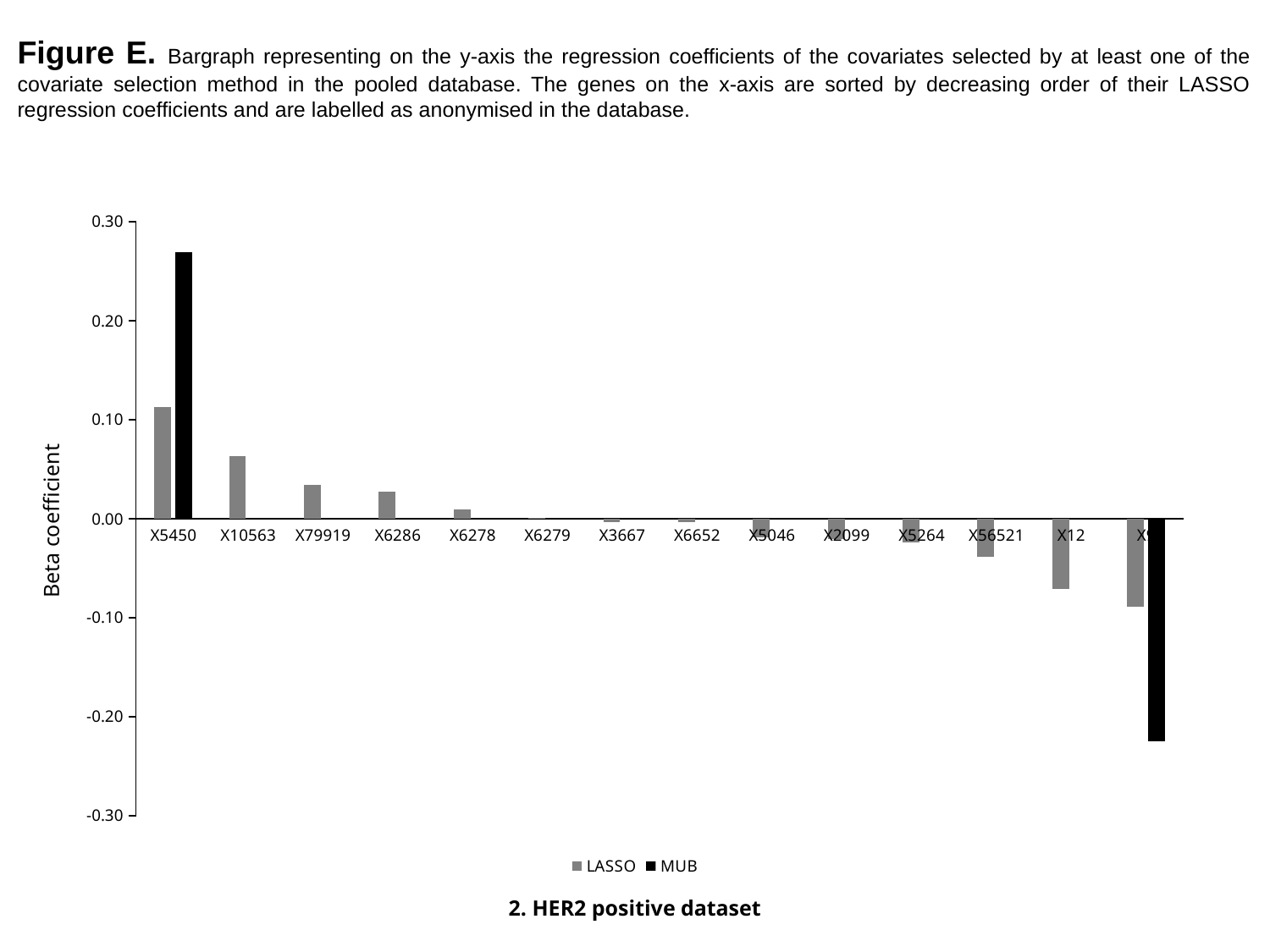

Figure E. Bargraph representing on the y-axis the regression coefficients of the covariates selected by at least one of the covariate selection method in the pooled database. The genes on the x-axis are sorted by decreasing order of their LASSO regression coefficients and are labelled as anonymised in the database.
### Chart
| Category | LASSO | MUB |
|---|---|---|
| X5450 | 0.112381971322619 | 0.268847018271375 |
| X10563 | 0.0629485960231775 | 0.0 |
| X79919 | 0.0344031657983069 | 0.0 |
| X6286 | 0.0269910877261674 | 0.0 |
| X6278 | 0.00894437708952887 | 0.0 |
| X6279 | 0.00113708765585514 | 0.0 |
| X3667 | -0.00324379775945287 | 0.0 |
| X6652 | -0.00342825221699747 | 0.0 |
| X5046 | -0.0190299877847924 | 0.0 |
| X2099 | -0.0204812084618882 | 0.0 |
| X5264 | -0.0236305971713572 | 0.0 |
| X56521 | -0.0385097093861267 | 0.0 |
| X12 | -0.0708927651982128 | 0.0 |
| X9 | -0.0890595065675773 | -0.224779704253377 |Beta coefficient
2. HER2 positive dataset

## Slide 8
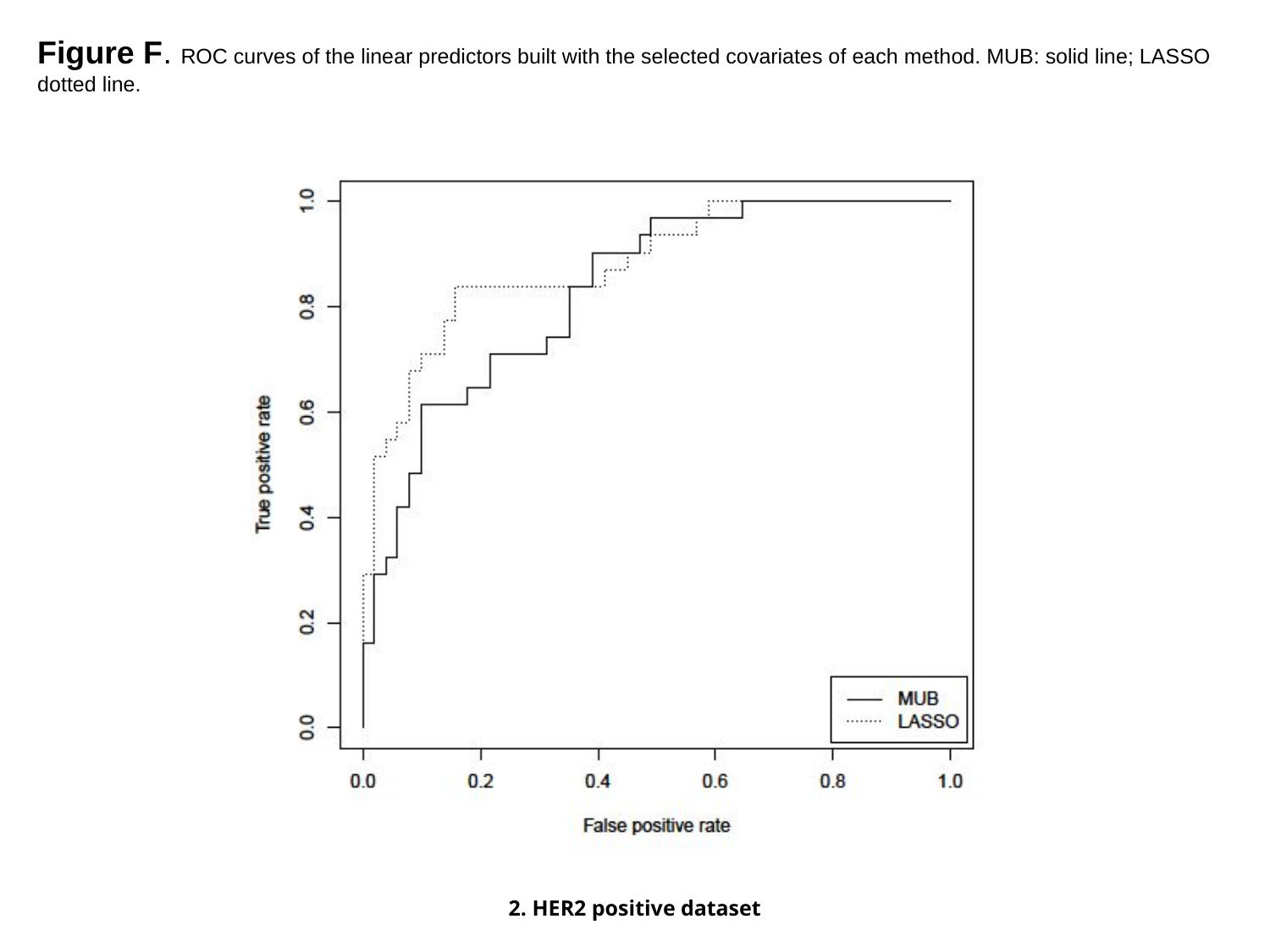

Figure F. ROC curves of the linear predictors built with the selected covariates of each method. MUB: solid line; LASSO dotted line.
2. HER2 positive dataset

## Slide 9
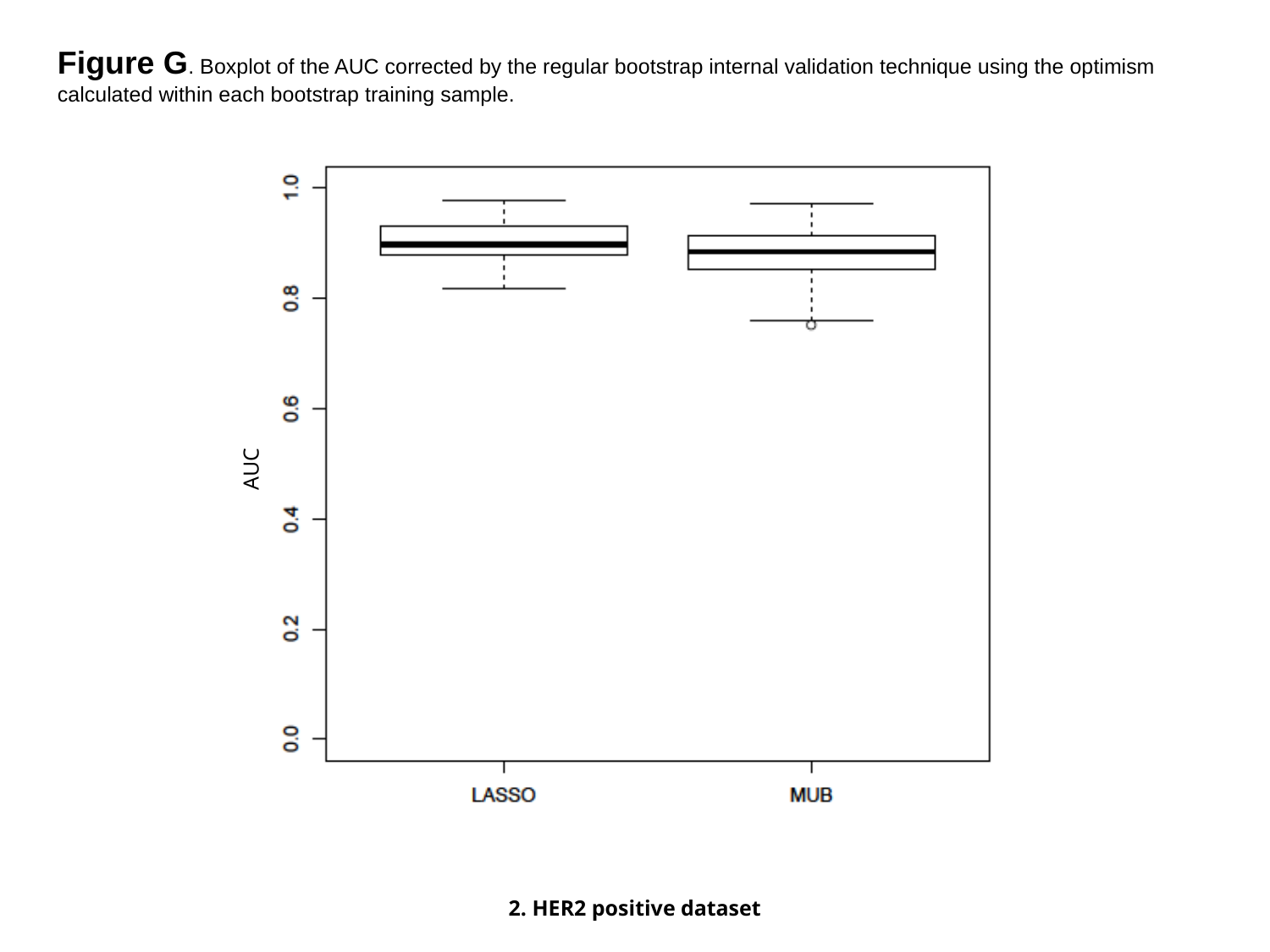

Figure G. Boxplot of the AUC corrected by the regular bootstrap internal validation technique using the optimism calculated within each bootstrap training sample.
AUC
2. HER2 positive dataset

## Slide 10
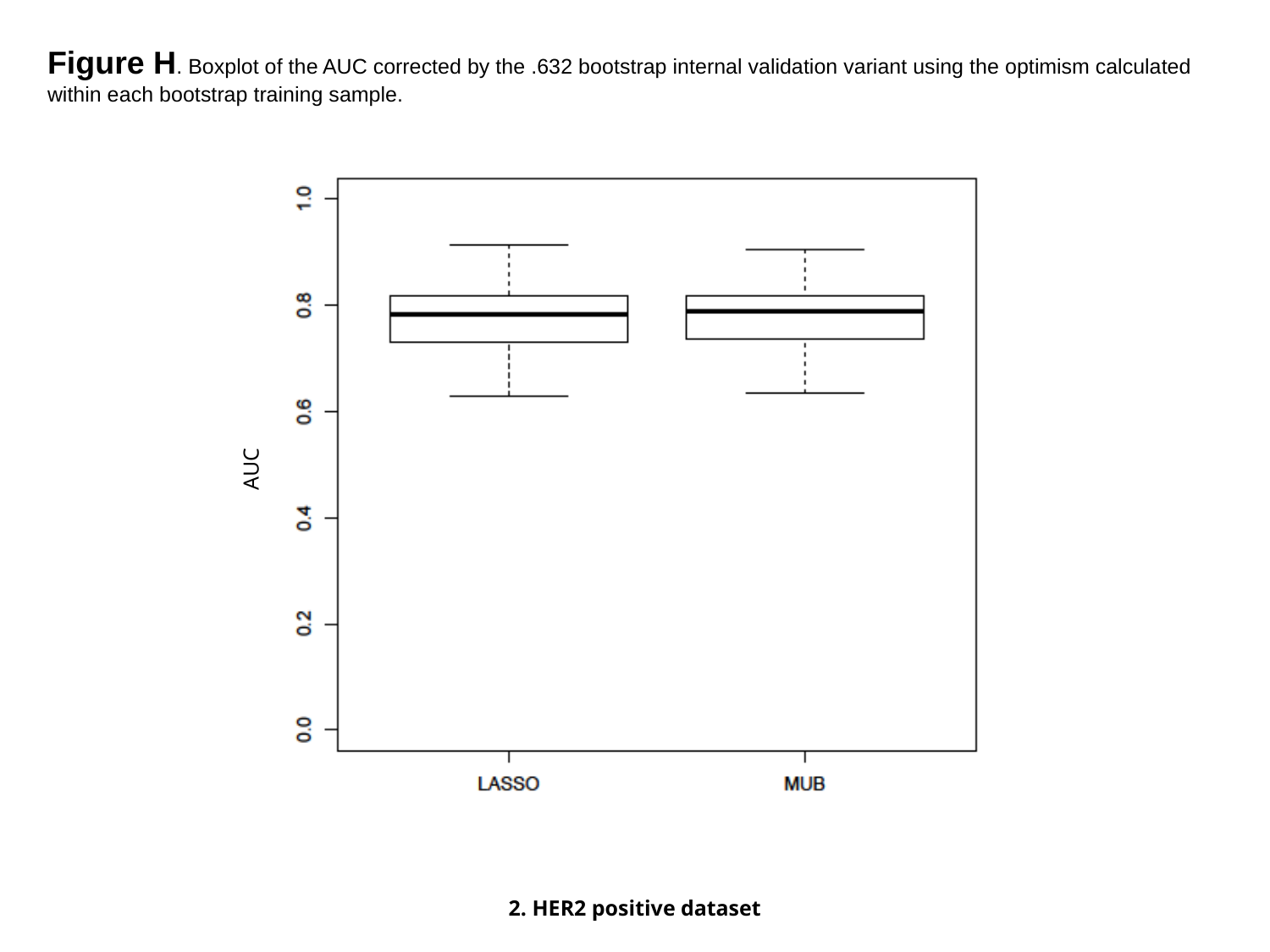

Figure H. Boxplot of the AUC corrected by the .632 bootstrap internal validation variant using the optimism calculated within each bootstrap training sample.
AUC
2. HER2 positive dataset

## Slide 11
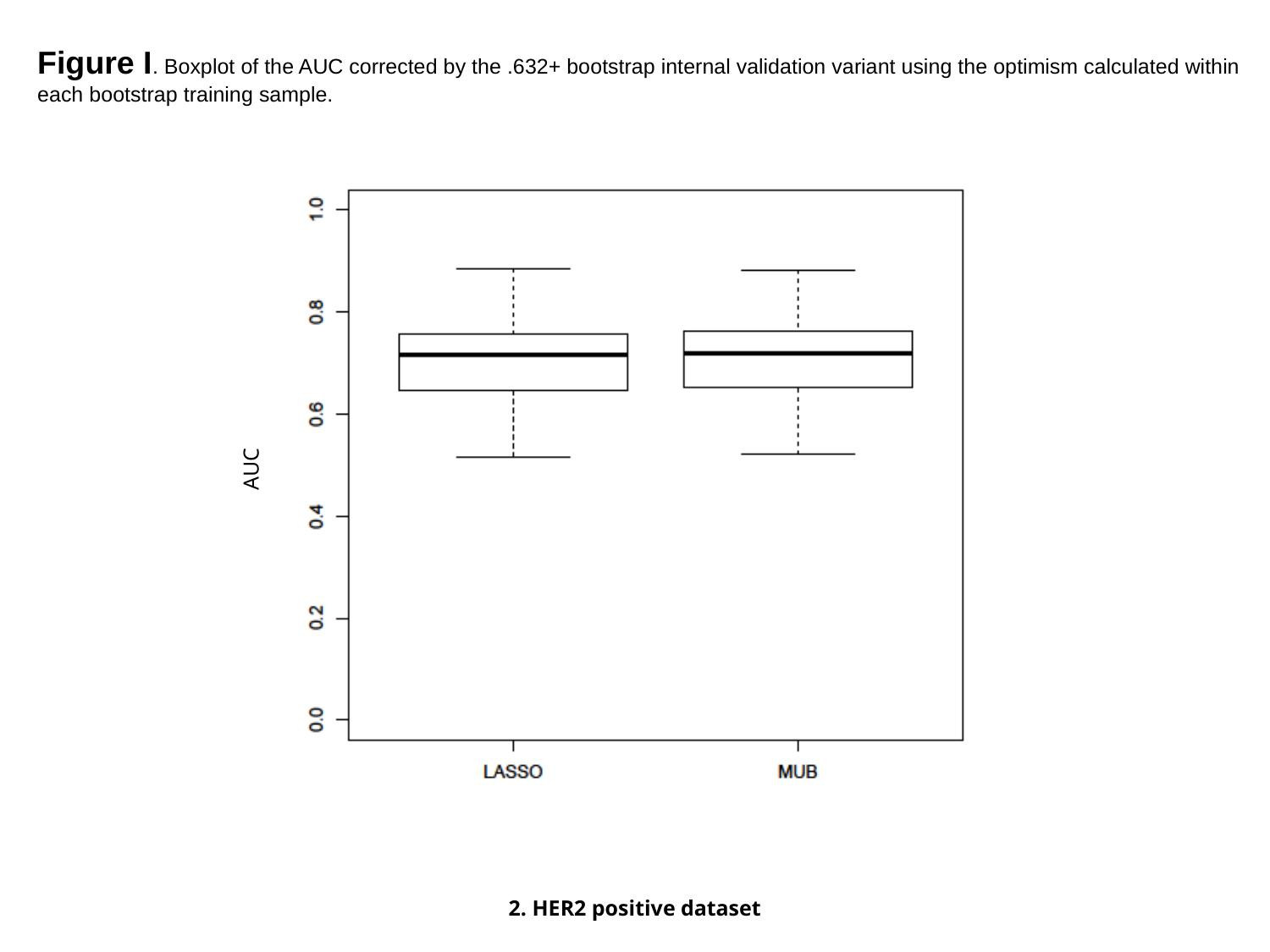

Figure I. Boxplot of the AUC corrected by the .632+ bootstrap internal validation variant using the optimism calculated within each bootstrap training sample.
AUC
2. HER2 positive dataset

## Slide 12
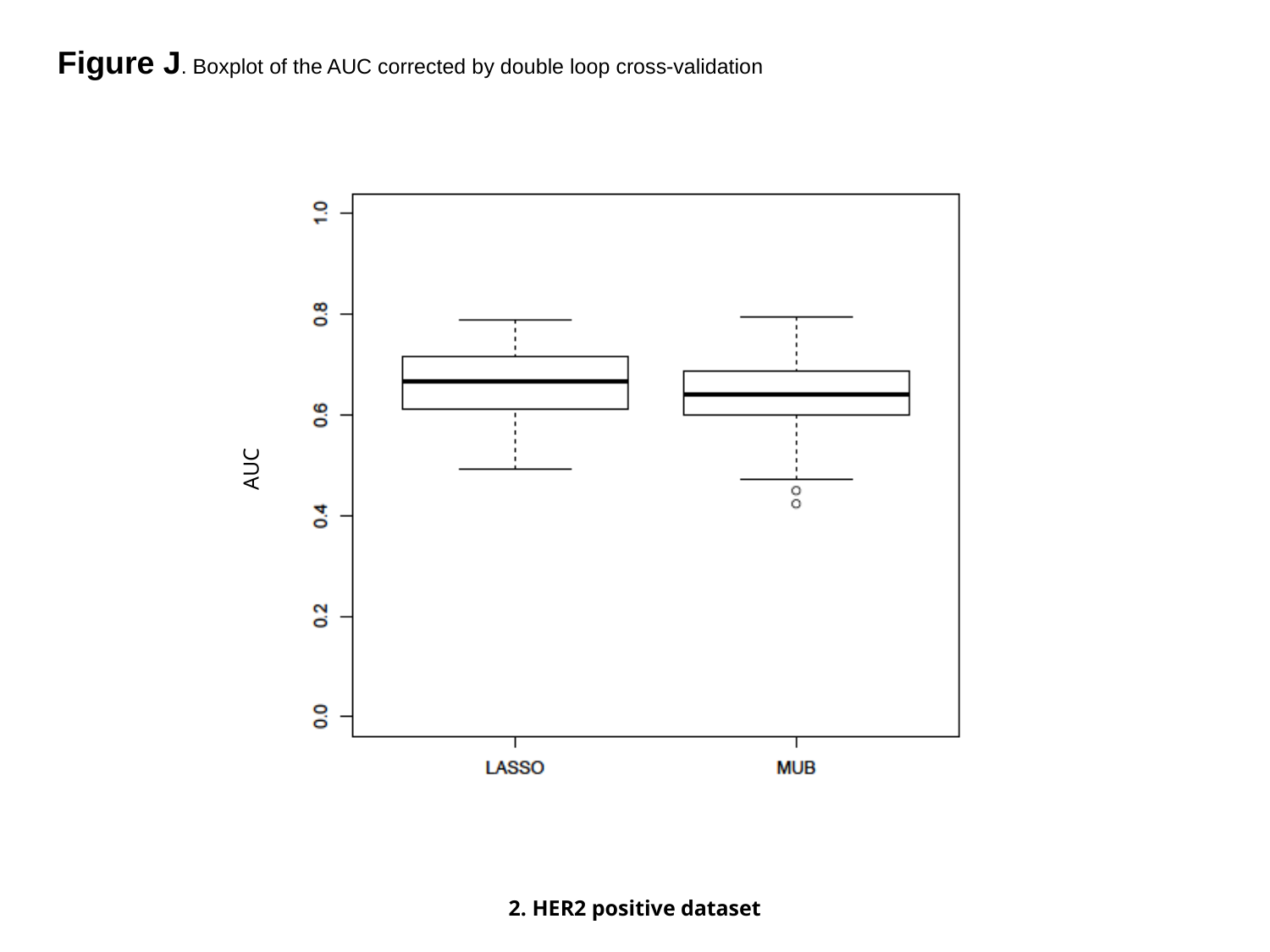

Figure J. Boxplot of the AUC corrected by double loop cross-validation
AUC
2. HER2 positive dataset

## Slide 13
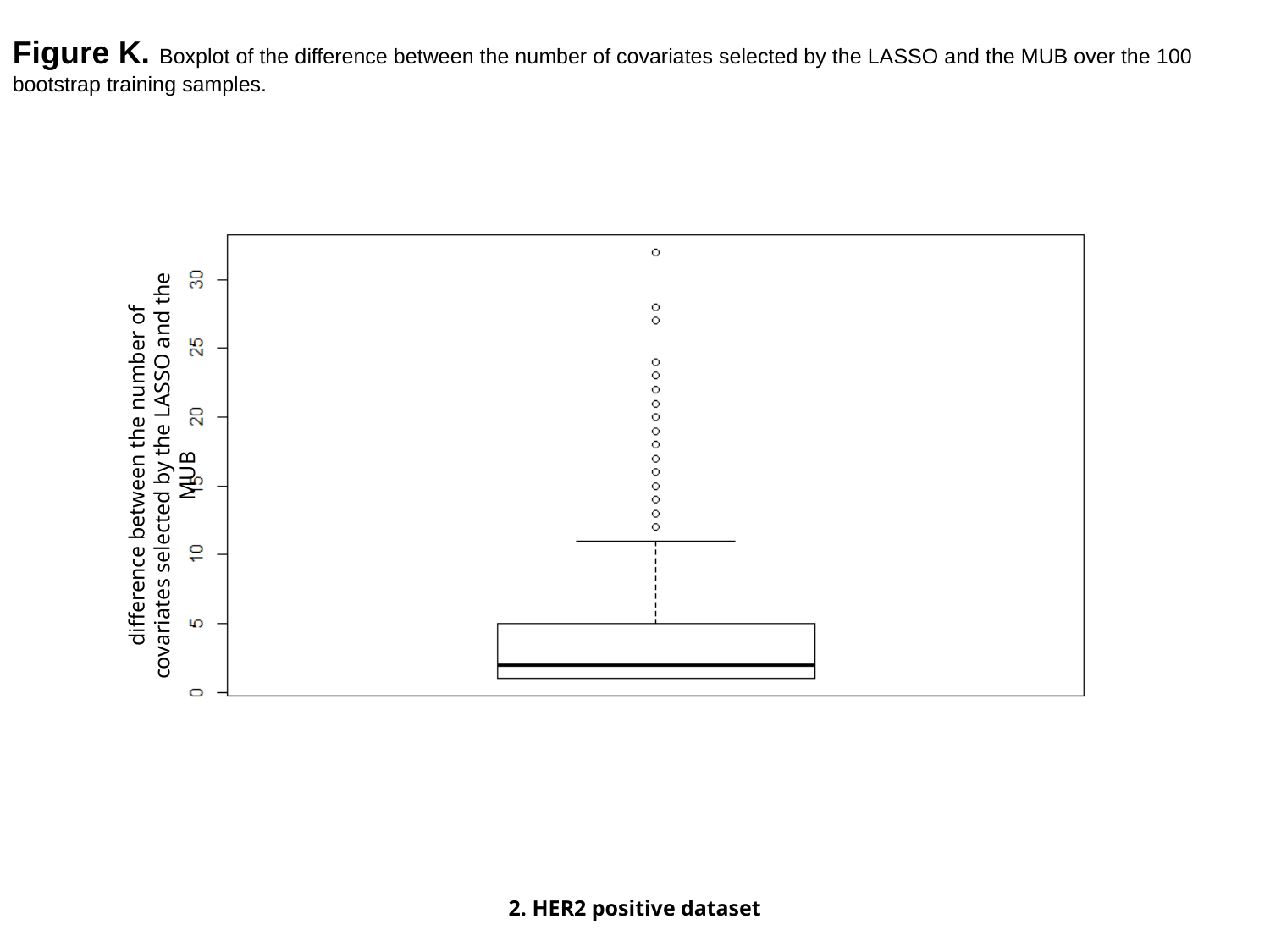

Figure K. Boxplot of the difference between the number of covariates selected by the LASSO and the MUB over the 100 bootstrap training samples.
difference between the number of covariates selected by the LASSO and the MUB
2. HER2 positive dataset

## Slide 14
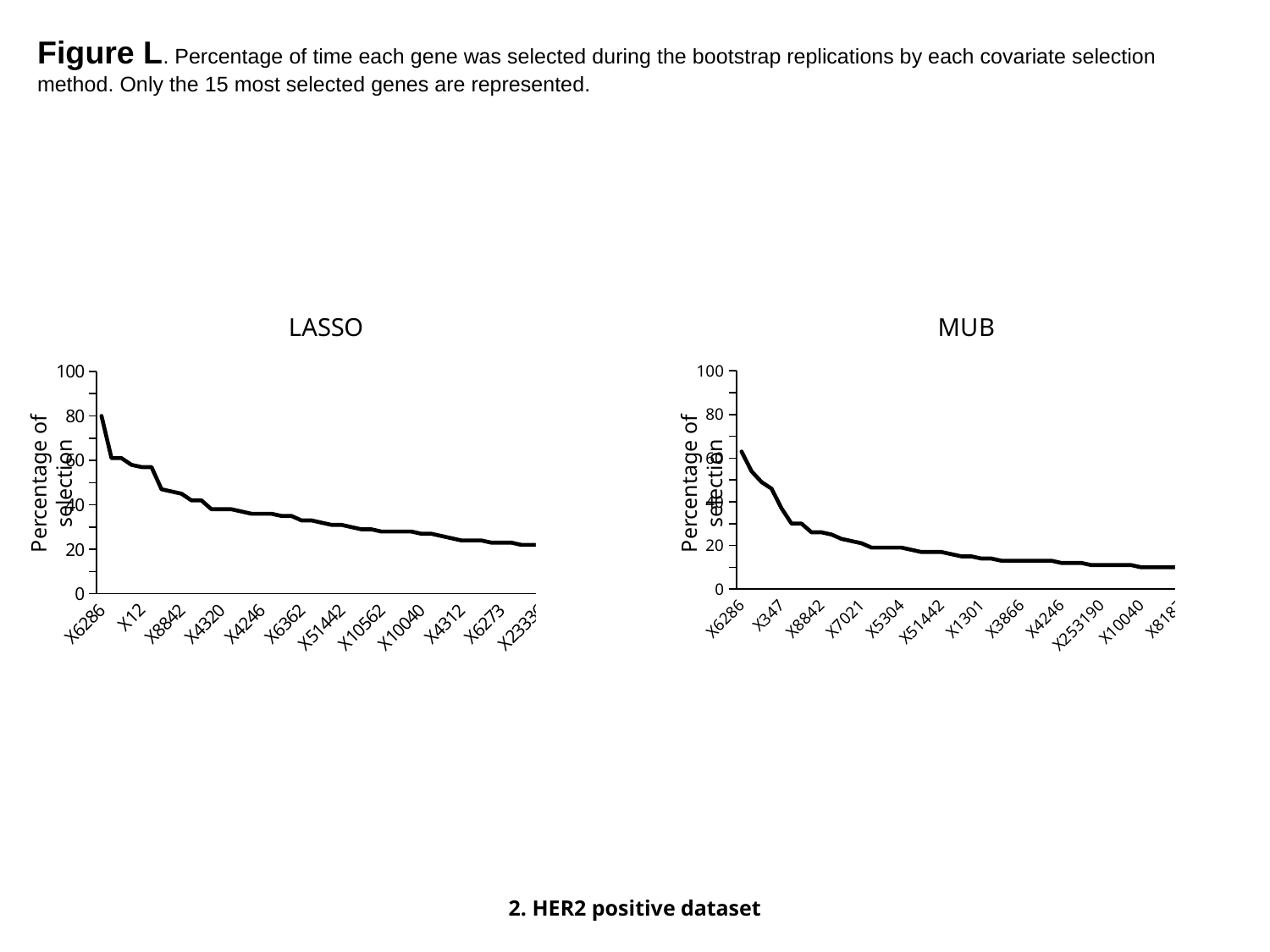

Figure L. Percentage of time each gene was selected during the bootstrap replications by each covariate selection method. Only the 15 most selected genes are represented.
### Chart:
| Category | LASSO |
|---|---|
| X6286 | 80.0 |
| X5450 | 61.0 |
| X79919 | 61.0 |
| X80736 | 58.0 |
| X12 | 57.0 |
| X347 | 57.0 |
| X5168 | 47.0 |
| X5304 | 46.0 |
| X8842 | 45.0 |
| X10321 | 42.0 |
| X10563 | 42.0 |
| X339479 | 38.0 |
| X4320 | 38.0 |
| X6898 | 38.0 |
| X2244 | 37.0 |
| X1301 | 36.0 |
| X4246 | 36.0 |
| X4969 | 36.0 |
| X5264 | 35.0 |
| X56521 | 35.0 |
| X6362 | 33.0 |
| X7021 | 33.0 |
| X3866 | 32.0 |
| X1081 | 31.0 |
| X51442 | 31.0 |
| X4283 | 30.0 |
| X4477 | 29.0 |
| X6278 | 29.0 |
| X10562 | 28.0 |
| X1728 | 28.0 |
| X2877 | 28.0 |
| X3929 | 28.0 |
| X10040 | 27.0 |
| X10103 | 27.0 |
| X4256 | 26.0 |
| X2001 | 25.0 |
| X4312 | 24.0 |
| X8476 | 24.0 |
| X8644 | 24.0 |
| X3212 | 23.0 |
| X6273 | 23.0 |
| X84525 | 23.0 |
| X1978 | 22.0 |
| X222 | 22.0 |
| X23336 | 22.0 |
| X1047 | 21.0 |
| X3434 | 21.0 |
| X7031 | 21.0 |
| X1360 | 20.0 |
| X4680 | 20.0 |
### Chart:
| Category | MUB |
|---|---|
| X6286 | 63.0 |
| X5450 | 54.0 |
| X12 | 49.0 |
| X79919 | 46.0 |
| X347 | 37.0 |
| X10563 | 30.0 |
| X80736 | 30.0 |
| X5264 | 26.0 |
| X8842 | 26.0 |
| X56521 | 25.0 |
| X6362 | 23.0 |
| X10321 | 22.0 |
| X7021 | 21.0 |
| X10103 | 19.0 |
| X2244 | 19.0 |
| X4283 | 19.0 |
| X5304 | 19.0 |
| X6898 | 18.0 |
| X1728 | 17.0 |
| X339479 | 17.0 |
| X51442 | 17.0 |
| X6278 | 16.0 |
| X4320 | 15.0 |
| X5168 | 15.0 |
| X1301 | 14.0 |
| X9498 | 14.0 |
| X1978 | 13.0 |
| X2877 | 13.0 |
| X3866 | 13.0 |
| X4969 | 13.0 |
| X79844 | 13.0 |
| X8476 | 13.0 |
| X4246 | 12.0 |
| X4312 | 12.0 |
| X4477 | 12.0 |
| X10562 | 11.0 |
| X253190 | 11.0 |
| X3929 | 11.0 |
| X6273 | 11.0 |
| X9 | 11.0 |
| X10040 | 10.0 |
| X4256 | 10.0 |
| X6279 | 10.0 |
| X7031 | 10.0 |
| X8187 | 10.0 |
| X10551 | 9.0 |
| X3512 | 9.0 |
| X4680 | 9.0 |
| X4830 | 9.0 |
| X8644 | 9.0 |Percentage of selection
Percentage of selection
2. HER2 positive dataset

## Slide 15
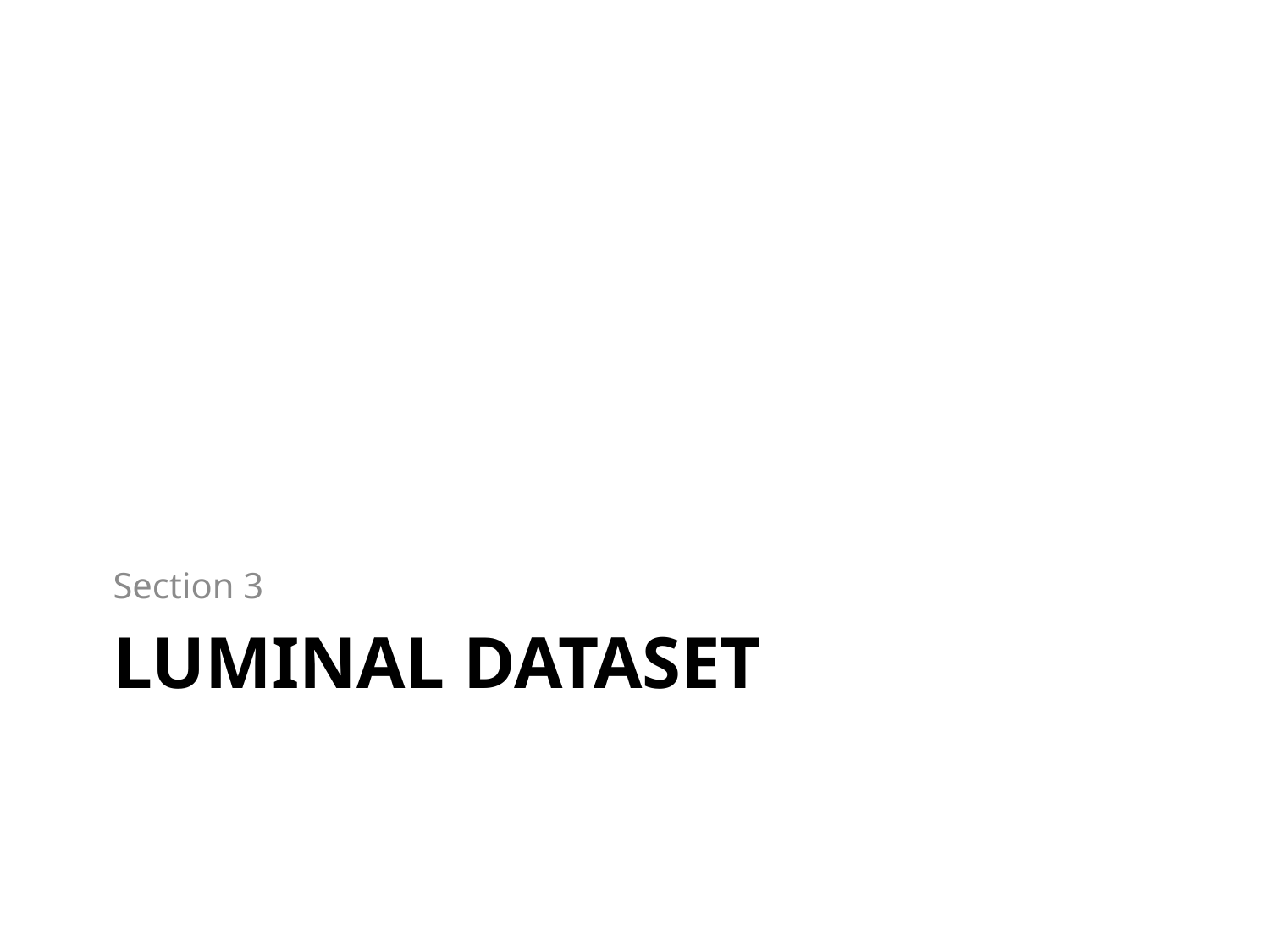

Section 3
# Luminal dataset

## Slide 16
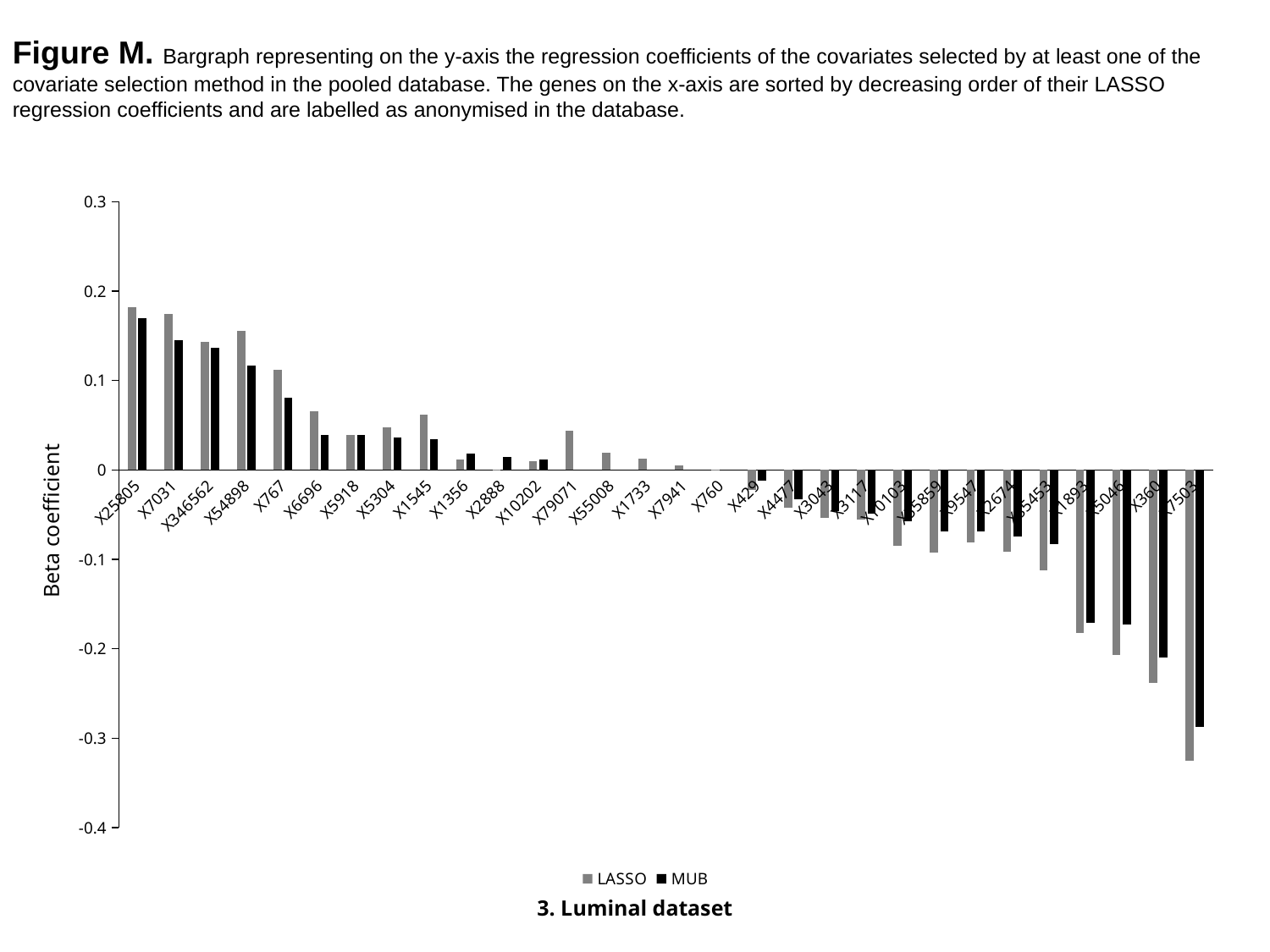

Figure M. Bargraph representing on the y-axis the regression coefficients of the covariates selected by at least one of the covariate selection method in the pooled database. The genes on the x-axis are sorted by decreasing order of their LASSO regression coefficients and are labelled as anonymised in the database.
### Chart
| Category | LASSO | MUB |
|---|---|---|
| X25805 | 0.182412736362159 | 0.169326500192991 |
| X7031 | 0.174885606642038 | 0.145396083796539 |
| X346562 | 0.142932581225698 | 0.136695904230077 |
| X54898 | 0.155216686636199 | 0.116868803411908 |
| X767 | 0.112072182960234 | 0.0812115945954413 |
| X6696 | 0.0655682519731966 | 0.0393415648590942 |
| X5918 | 0.0394016858586637 | 0.0391474344893026 |
| X5304 | 0.0476624119364937 | 0.0363746385473794 |
| X1545 | 0.0614624847305024 | 0.0341791670353592 |
| X1356 | 0.0115466112151075 | 0.018217371711359 |
| X2888 | 0.00079704868803113 | 0.0143552016767088 |
| X10202 | 0.0100215579104788 | 0.011327607272446 |
| X79071 | 0.0441774153559274 | 0.0 |
| X55008 | 0.019294596127639 | 0.0 |
| X1733 | 0.0125599229406096 | 0.0 |
| X7941 | 0.00513675048659385 | 0.0 |
| X760 | -0.000658830000693749 | 0.0 |
| X429 | -0.0222099813182124 | -0.0118411947949532 |
| X4477 | -0.0427060651735471 | -0.033112644195549 |
| X3043 | -0.0534617350970575 | -0.0463332988853705 |
| X3117 | -0.0555442186792334 | -0.0486718092125718 |
| X10103 | -0.0844800207720273 | -0.0572026606928612 |
| X55859 | -0.0919507595926857 | -0.0684487374205696 |
| X9547 | -0.0809825540887369 | -0.0688565645104432 |
| X2674 | -0.0919190929014118 | -0.0745734470978475 |
| X85453 | -0.112717463527584 | -0.0826056432917794 |
| X1893 | -0.182777900093415 | -0.170821364282852 |
| X5046 | -0.206617100345796 | -0.172785330594734 |
| X360 | -0.237796685225295 | -0.209495357839321 |
| X7503 | -0.324956994390778 | -0.28778073997975 |Beta coefficient
3. Luminal dataset

## Slide 17
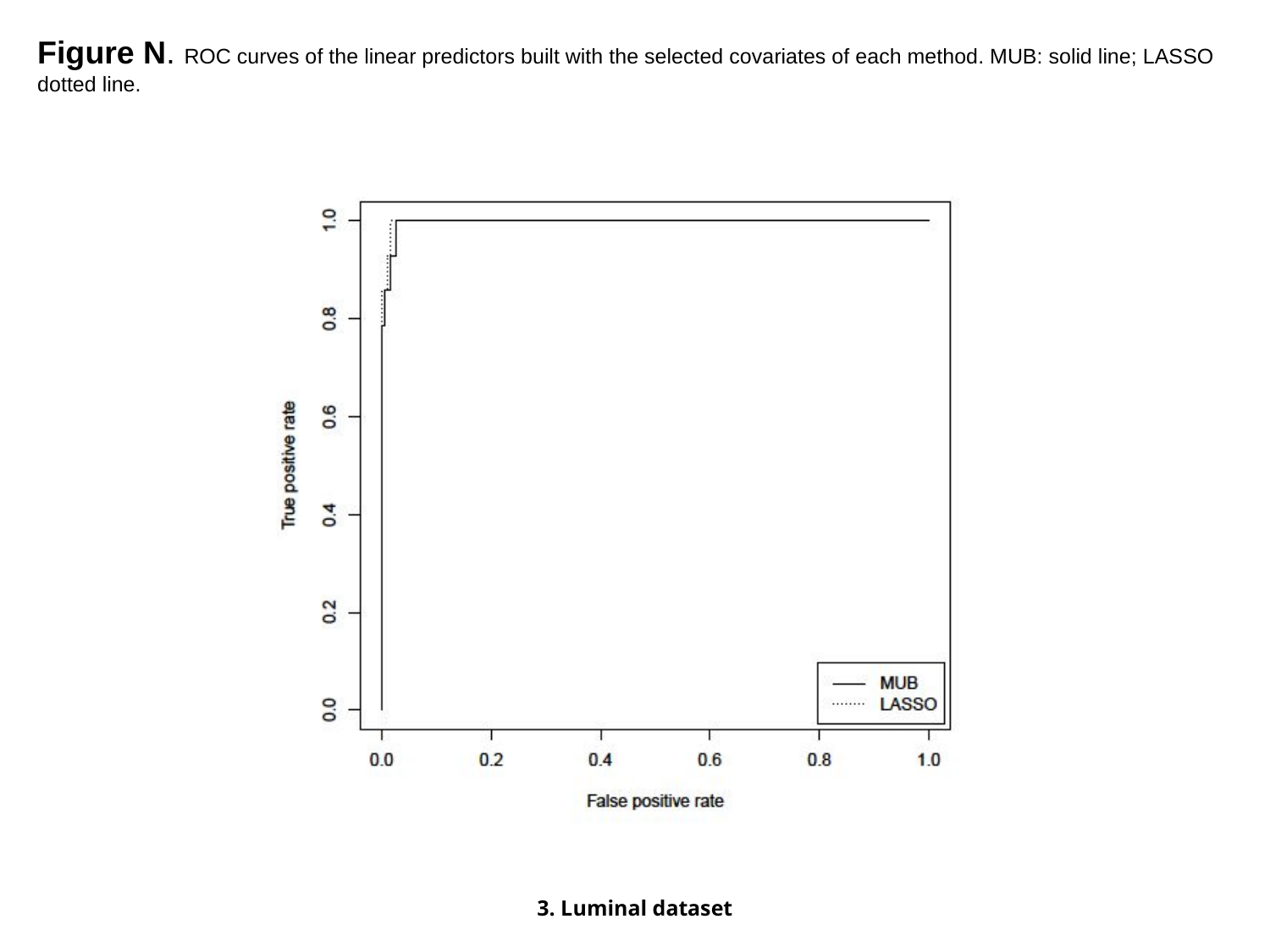

Figure N. ROC curves of the linear predictors built with the selected covariates of each method. MUB: solid line; LASSO dotted line.
3. Luminal dataset

## Slide 18
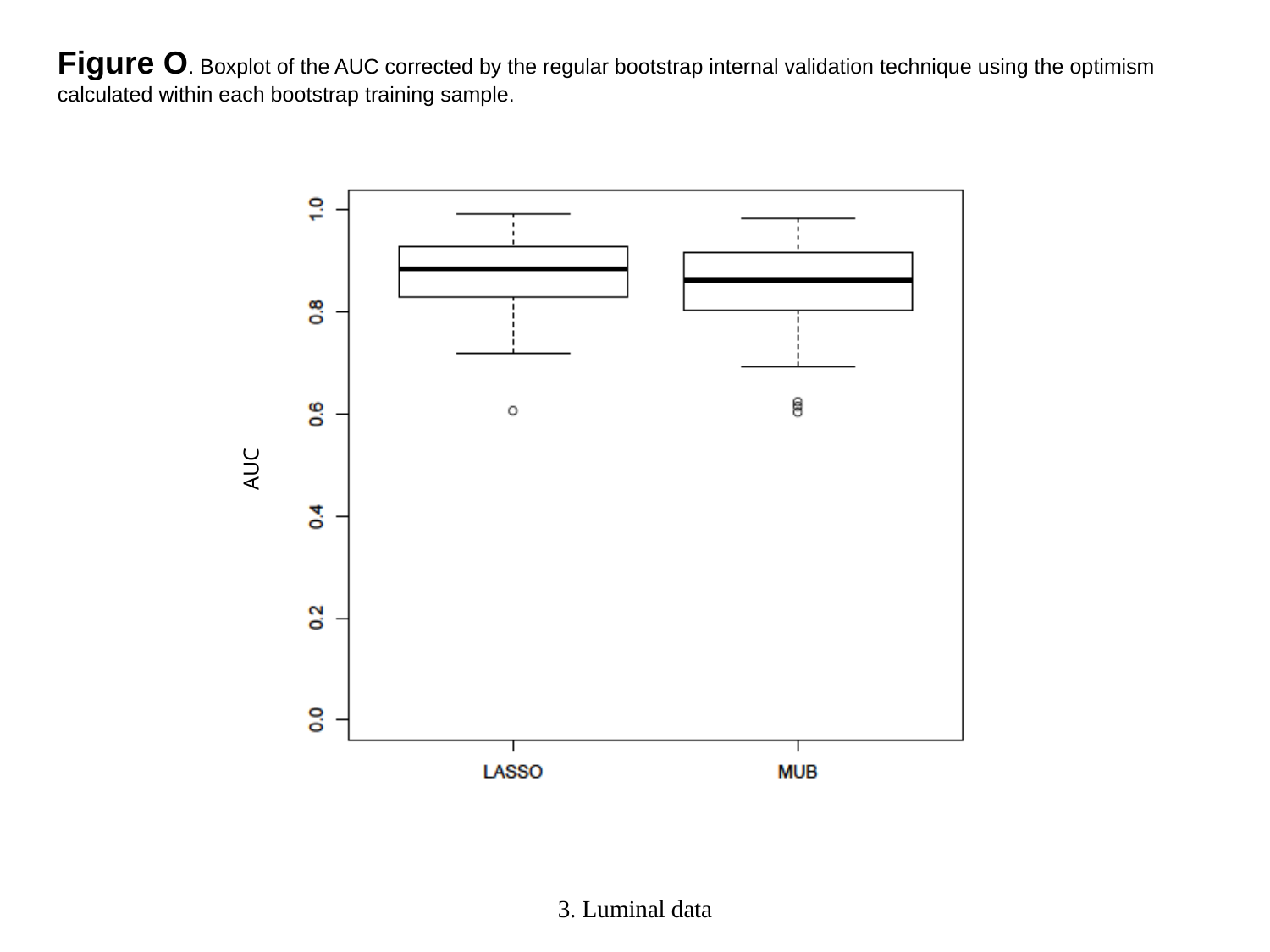

Figure O. Boxplot of the AUC corrected by the regular bootstrap internal validation technique using the optimism calculated within each bootstrap training sample.
AUC
3. Luminal data

## Slide 19
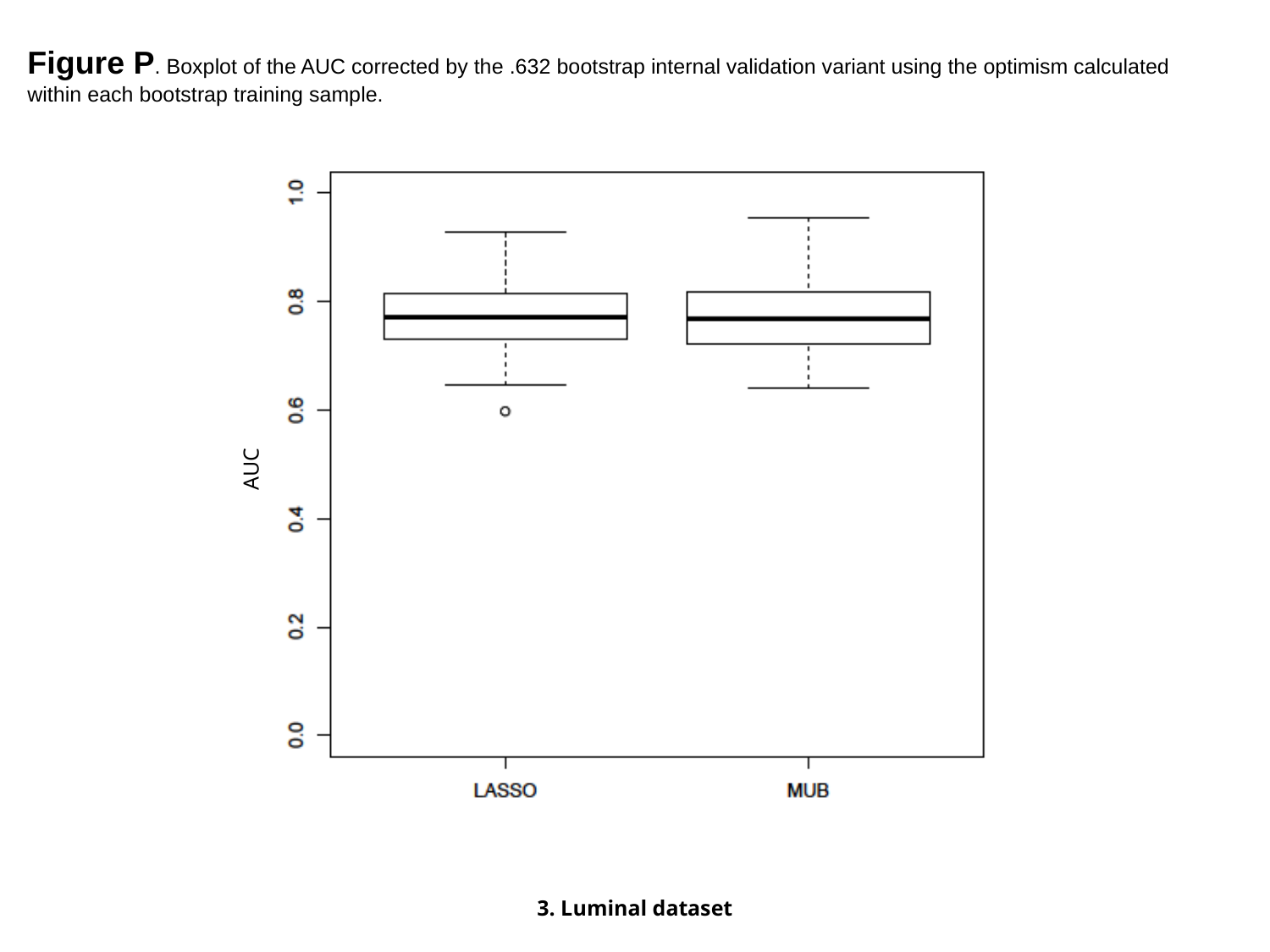

Figure P. Boxplot of the AUC corrected by the .632 bootstrap internal validation variant using the optimism calculated within each bootstrap training sample.
AUC
3. Luminal dataset

## Slide 20
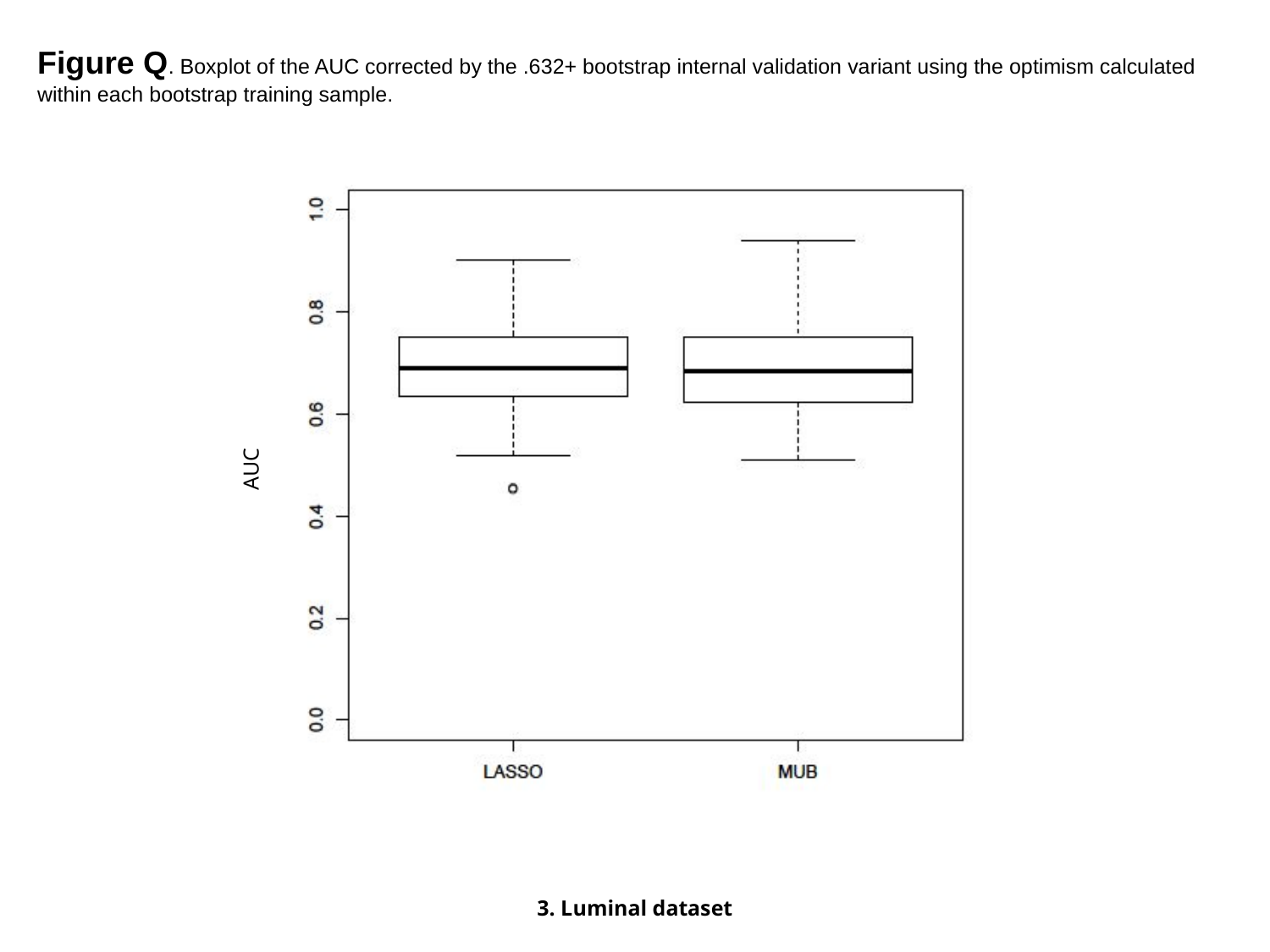

Figure Q. Boxplot of the AUC corrected by the .632+ bootstrap internal validation variant using the optimism calculated within each bootstrap training sample.
AUC
3. Luminal dataset

## Slide 21
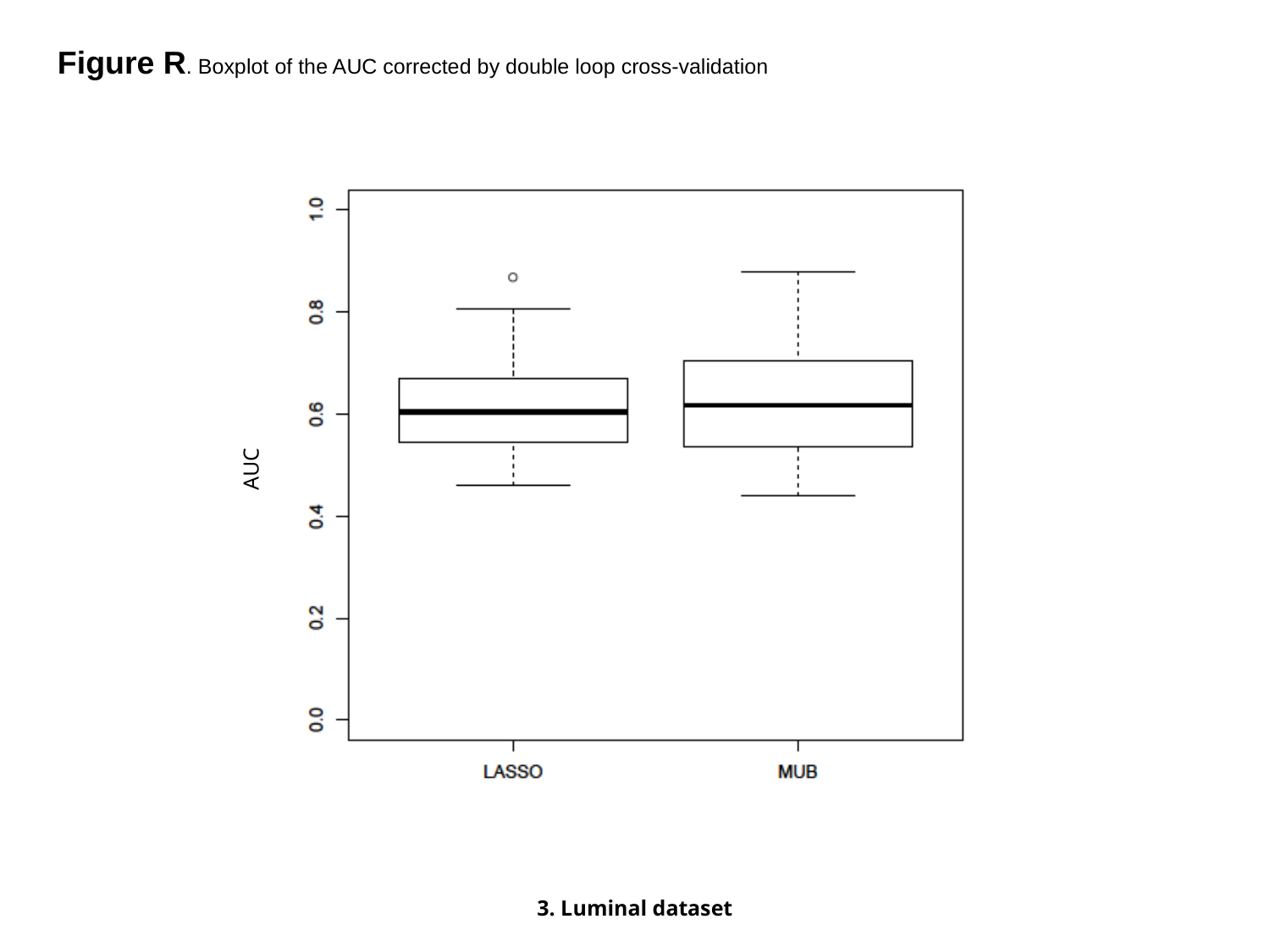

Figure R. Boxplot of the AUC corrected by double loop cross-validation
AUC
3. Luminal dataset

## Slide 22
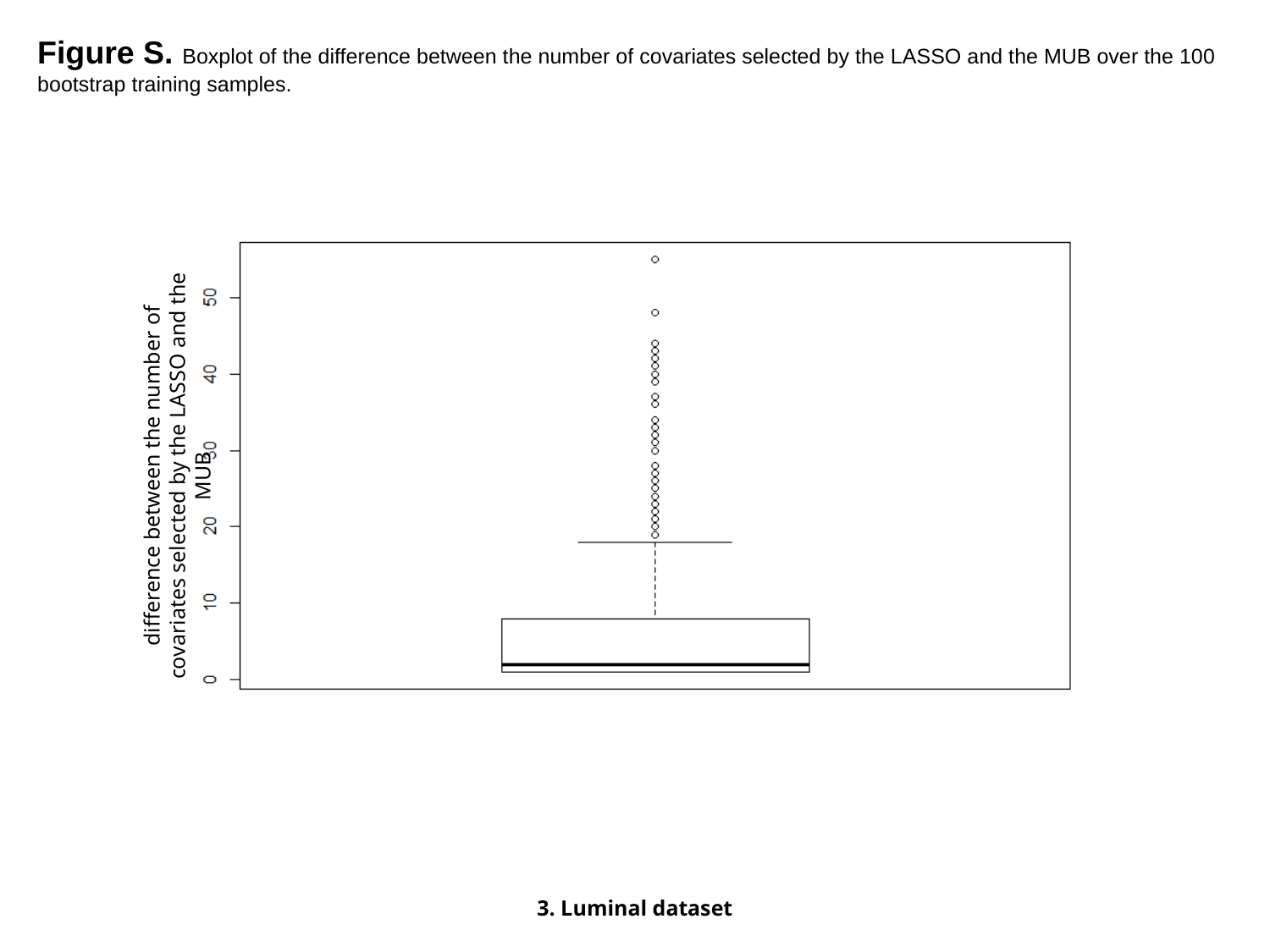

Figure S. Boxplot of the difference between the number of covariates selected by the LASSO and the MUB over the 100 bootstrap training samples.
difference between the number of covariates selected by the LASSO and the MUB
3. Luminal dataset

## Slide 23
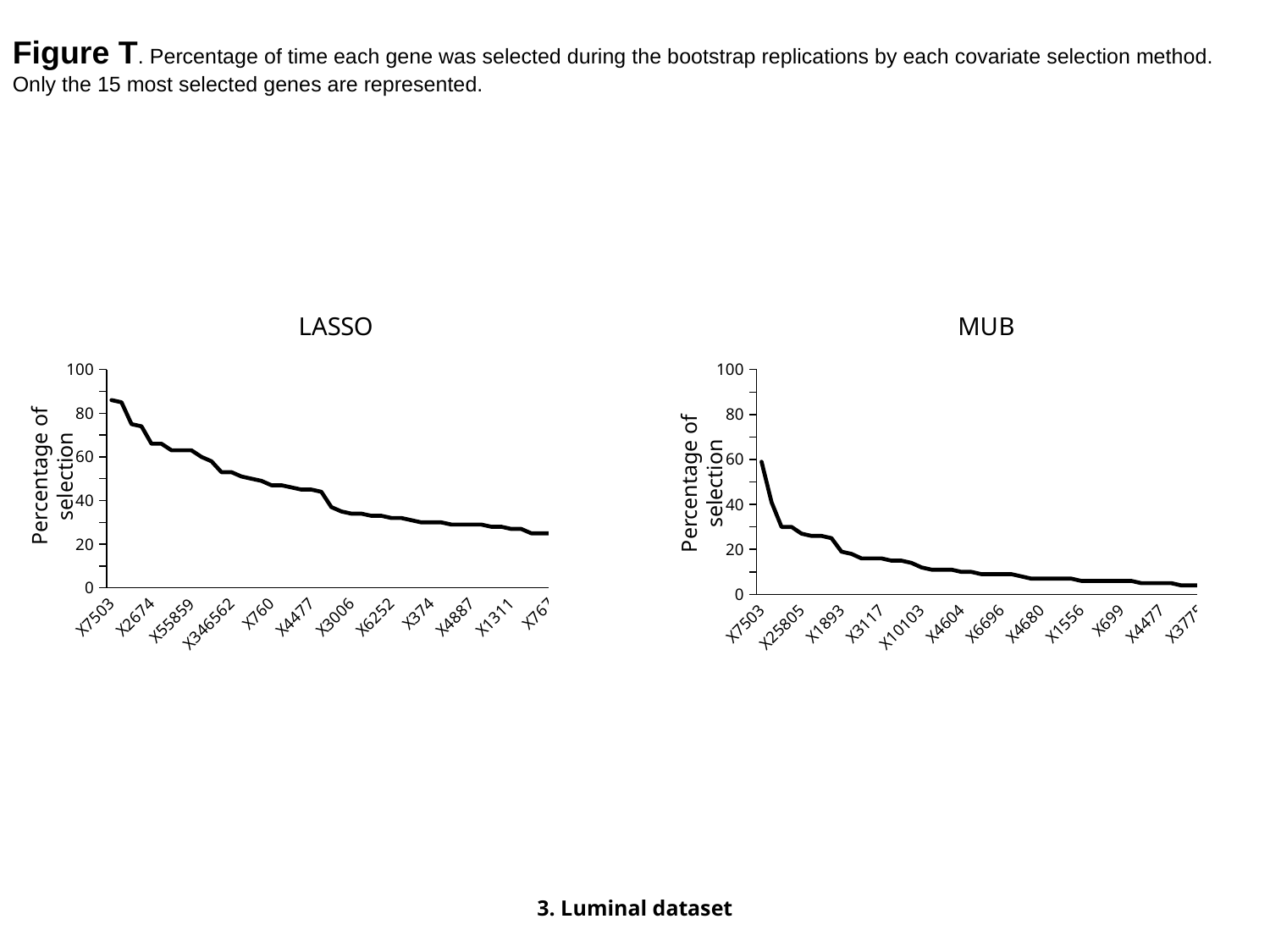

Figure T. Percentage of time each gene was selected during the bootstrap replications by each covariate selection method. Only the 15 most selected genes are represented.
### Chart:
| Category | LASSO |
|---|---|
| X7503 | 86.0 |
| X360 | 85.0 |
| X25805 | 75.0 |
| X7031 | 74.0 |
| X2674 | 66.0 |
| X85453 | 66.0 |
| X5304 | 63.0 |
| X54898 | 63.0 |
| X55859 | 63.0 |
| X10202 | 60.0 |
| X1893 | 58.0 |
| X3117 | 53.0 |
| X346562 | 53.0 |
| X5046 | 51.0 |
| X3043 | 50.0 |
| X2888 | 49.0 |
| X760 | 47.0 |
| X9547 | 47.0 |
| X1556 | 46.0 |
| X11283 | 45.0 |
| X4477 | 45.0 |
| X10103 | 44.0 |
| X4680 | 37.0 |
| X1545 | 35.0 |
| X3006 | 34.0 |
| X4604 | 34.0 |
| X6696 | 33.0 |
| X9536 | 33.0 |
| X6252 | 32.0 |
| X79589 | 32.0 |
| X4283 | 31.0 |
| X1733 | 30.0 |
| X374 | 30.0 |
| X7021 | 30.0 |
| X1047 | 29.0 |
| X3119 | 29.0 |
| X4887 | 29.0 |
| X5918 | 29.0 |
| X4246 | 28.0 |
| X429 | 28.0 |
| X1311 | 27.0 |
| X247 | 27.0 |
| X2167 | 25.0 |
| X347902 | 25.0 |
| X767 | 25.0 |
| X4137 | 24.0 |
| X4250 | 24.0 |
| X55008 | 24.0 |
| X7138 | 24.0 |
| X79071 | 24.0 |
### Chart:
| Category | MUB |
|---|---|
| X7503 | 59.0 |
| X360 | 41.0 |
| X346562 | 30.0 |
| X54898 | 30.0 |
| X25805 | 27.0 |
| X7031 | 26.0 |
| X85453 | 26.0 |
| X2674 | 25.0 |
| X1893 | 19.0 |
| X10202 | 18.0 |
| X2888 | 16.0 |
| X3043 | 16.0 |
| X3117 | 16.0 |
| X5046 | 15.0 |
| X5304 | 15.0 |
| X4887 | 14.0 |
| X10103 | 12.0 |
| X11283 | 11.0 |
| X1311 | 11.0 |
| X79589 | 11.0 |
| X4604 | 10.0 |
| X55008 | 10.0 |
| X1545 | 9.0 |
| X3006 | 9.0 |
| X6696 | 9.0 |
| X79071 | 9.0 |
| X55859 | 8.0 |
| X27250 | 7.0 |
| X4680 | 7.0 |
| X767 | 7.0 |
| X9547 | 7.0 |
| X9837 | 7.0 |
| X1556 | 6.0 |
| X3119 | 6.0 |
| X374 | 6.0 |
| X5918 | 6.0 |
| X699 | 6.0 |
| X7138 | 6.0 |
| X1733 | 5.0 |
| X2167 | 5.0 |
| X4477 | 5.0 |
| X7941 | 5.0 |
| X10267 | 4.0 |
| X1178 | 4.0 |
| X3775 | 4.0 |
| X4137 | 4.0 |
| X4751 | 4.0 |
| X7272 | 4.0 |
| X760 | 4.0 |
| X11212 | 3.0 |
Percentage of selection
Percentage of selection
3. Luminal dataset

## Slide 24
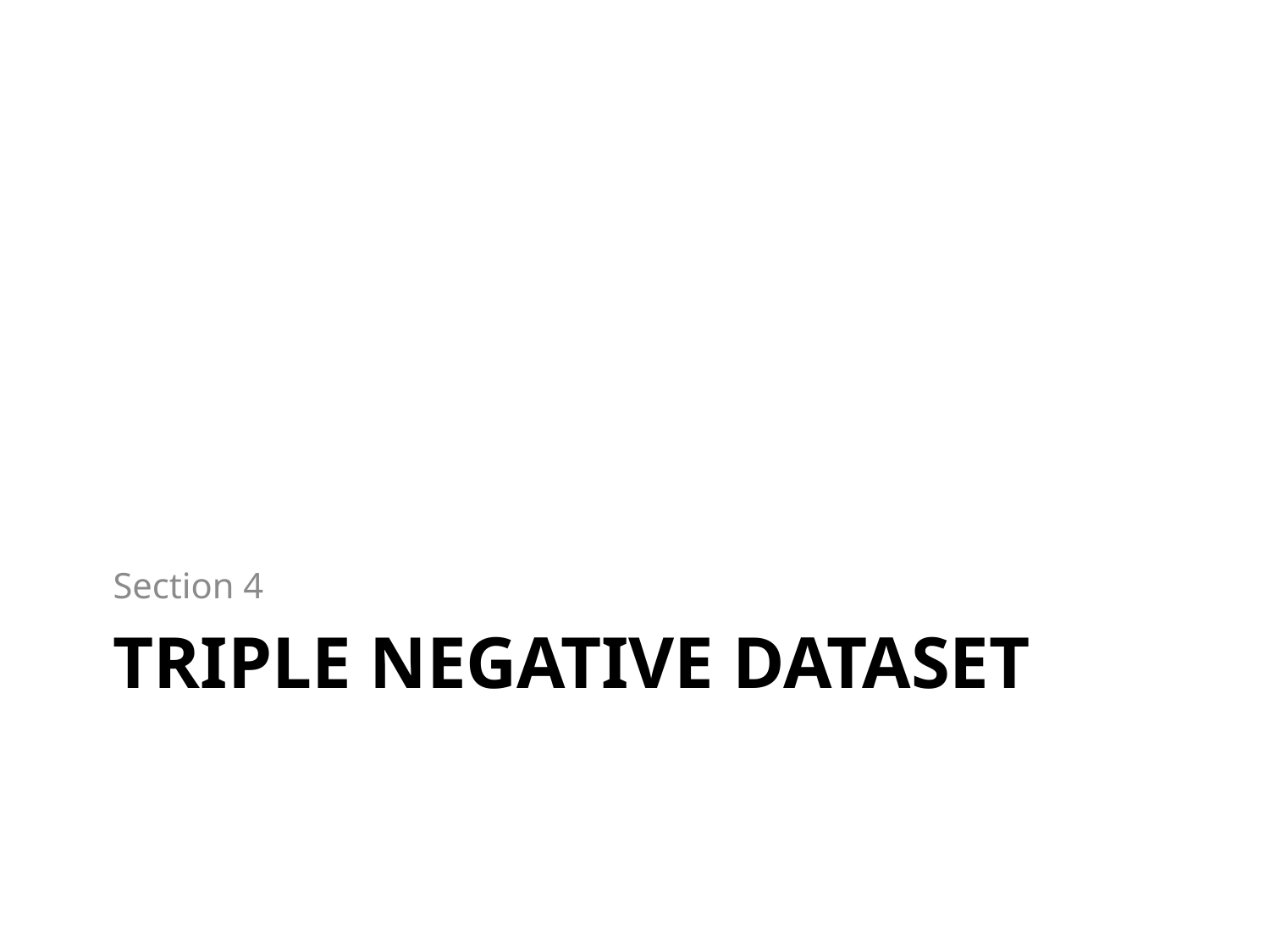

Section 4
# Triple negative dataset

## Slide 25
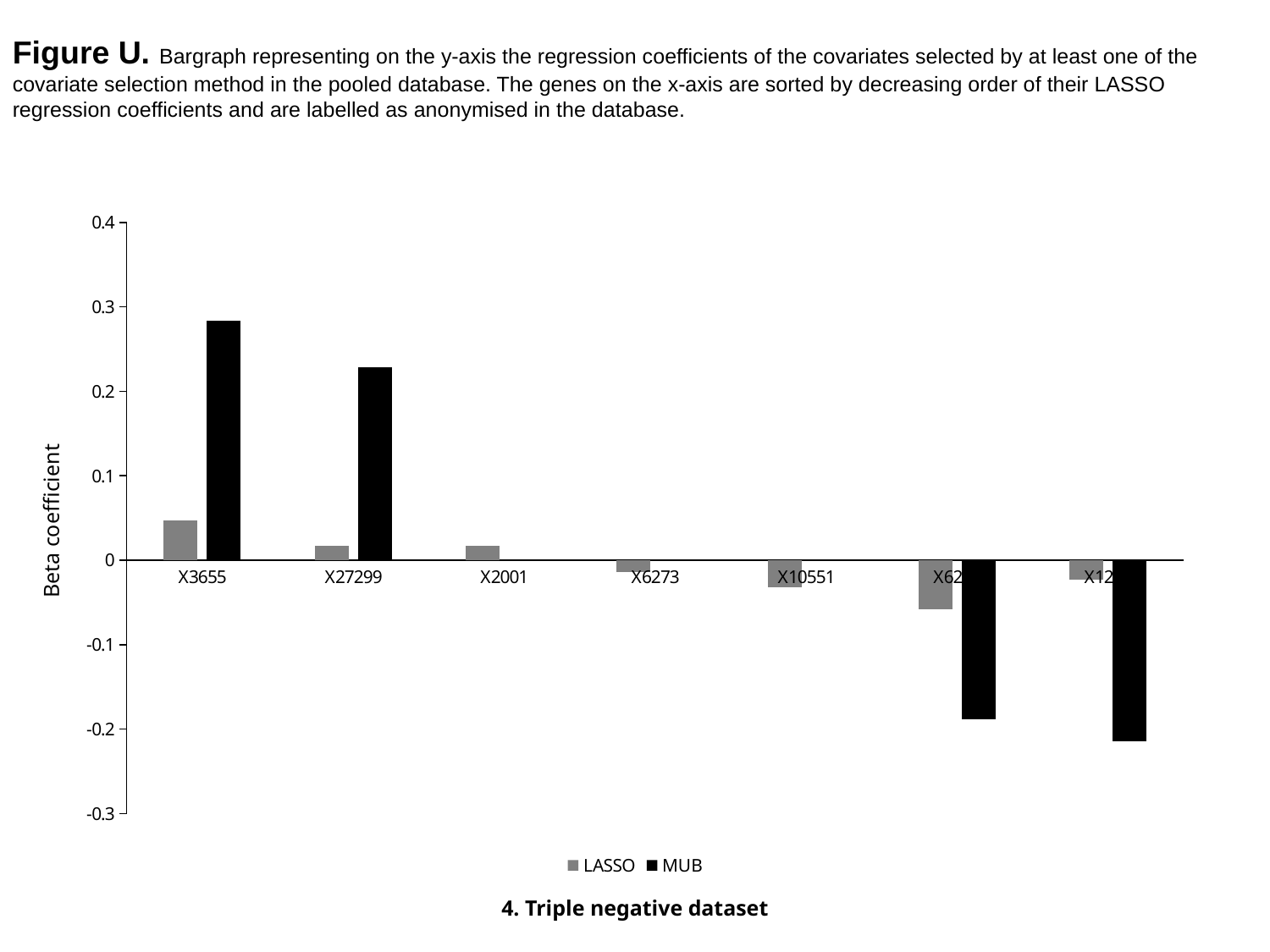

Figure U. Bargraph representing on the y-axis the regression coefficients of the covariates selected by at least one of the covariate selection method in the pooled database. The genes on the x-axis are sorted by decreasing order of their LASSO regression coefficients and are labelled as anonymised in the database.
### Chart
| Category | LASSO | MUB |
|---|---|---|
| X3655 | 0.0470360651743422 | 0.283322712753253 |
| X27299 | 0.0165314801630992 | 0.228344743425392 |
| X2001 | 0.0165266470516093 | 0.0 |
| X6273 | -0.0135405683373249 | 0.0 |
| X10551 | -0.0316758040341426 | 0.0 |
| X6286 | -0.0578724080003355 | -0.188758113273436 |
| X1277 | -0.0230921792892358 | -0.214276571352275 |Beta coefficient
4. Triple negative dataset

## Slide 26
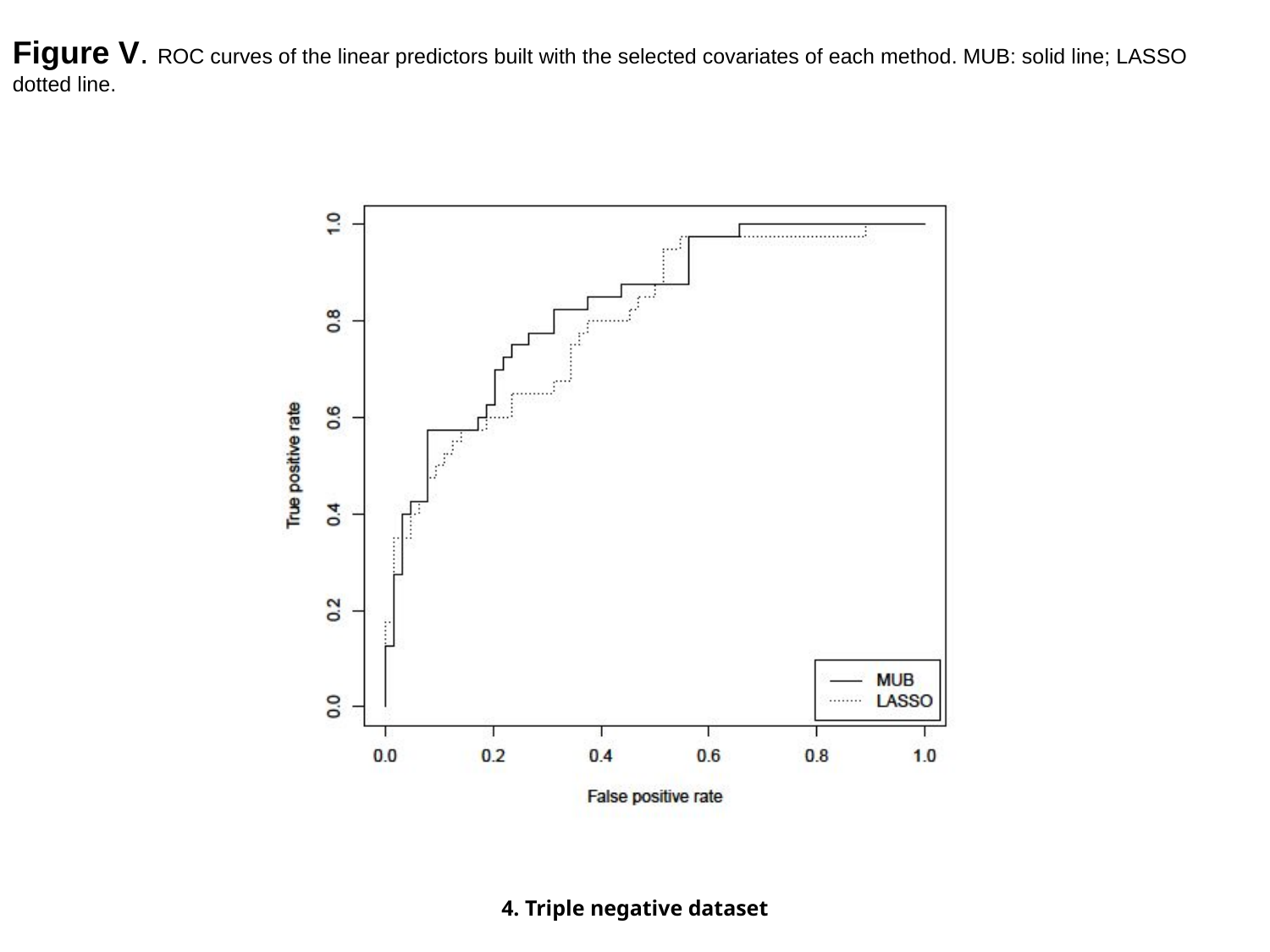

Figure V. ROC curves of the linear predictors built with the selected covariates of each method. MUB: solid line; LASSO dotted line.
4. Triple negative dataset

## Slide 27
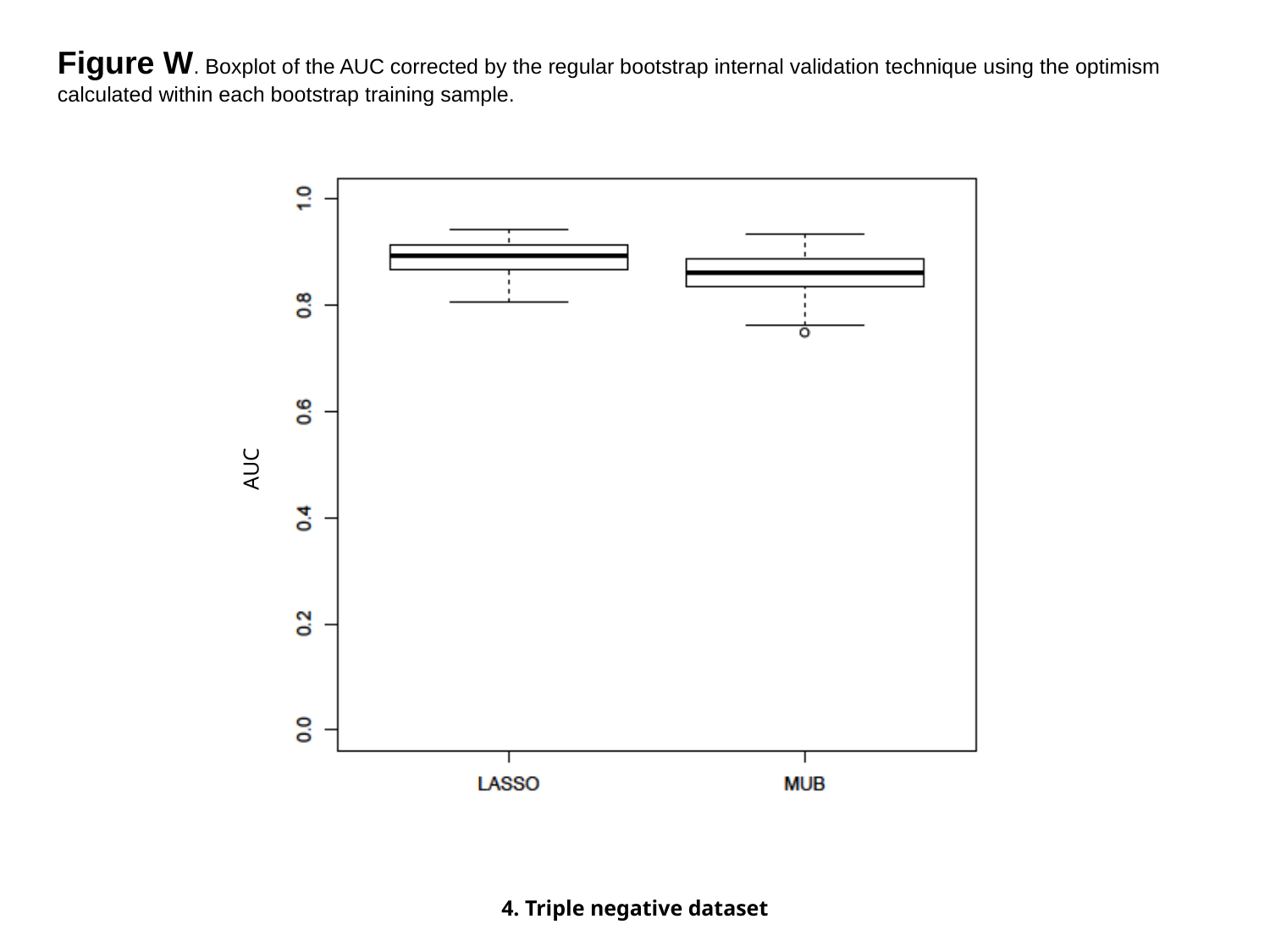

Figure W. Boxplot of the AUC corrected by the regular bootstrap internal validation technique using the optimism calculated within each bootstrap training sample.
AUC
4. Triple negative dataset

## Slide 28
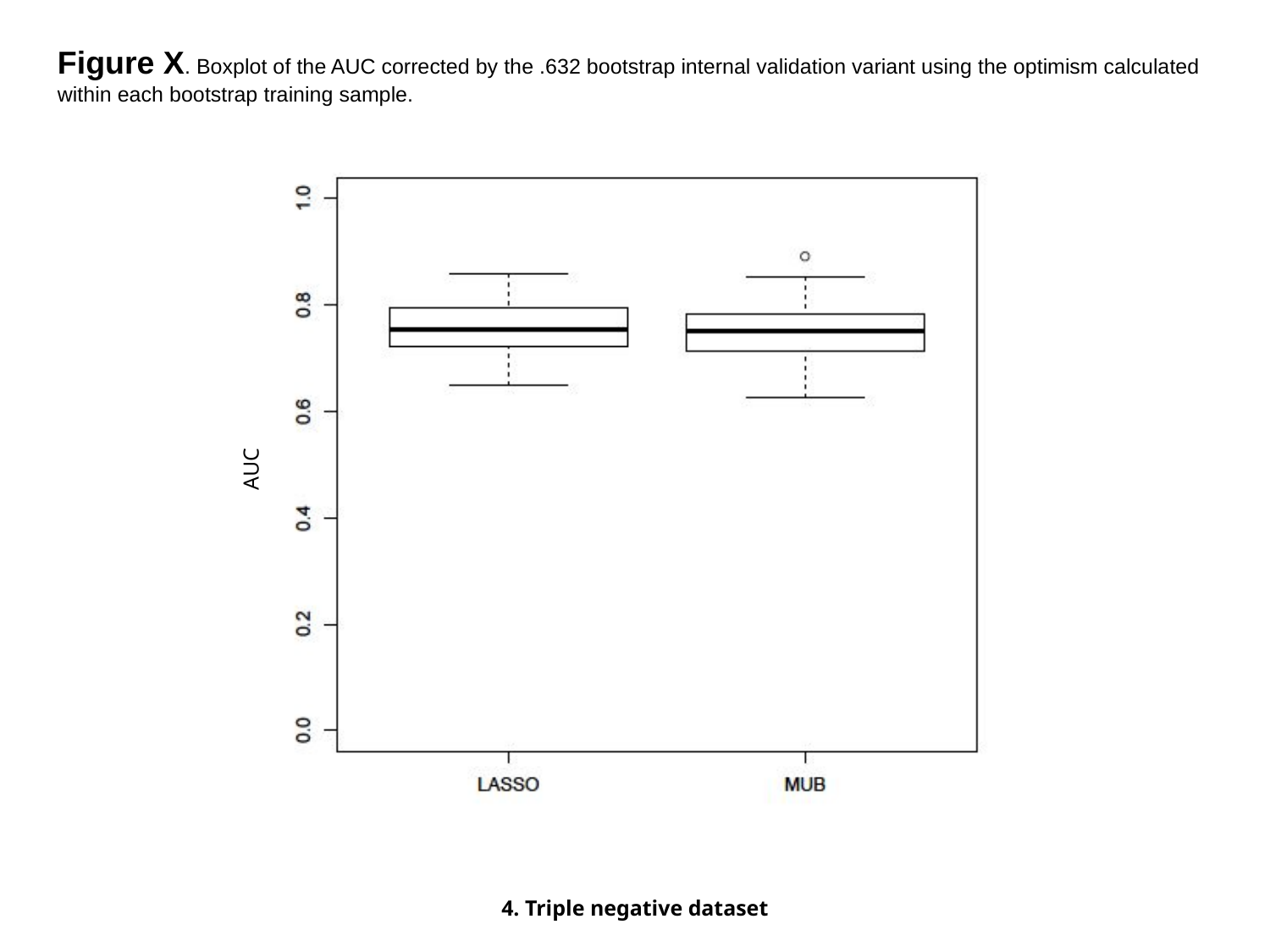

Figure X. Boxplot of the AUC corrected by the .632 bootstrap internal validation variant using the optimism calculated within each bootstrap training sample.
AUC
4. Triple negative dataset

## Slide 29
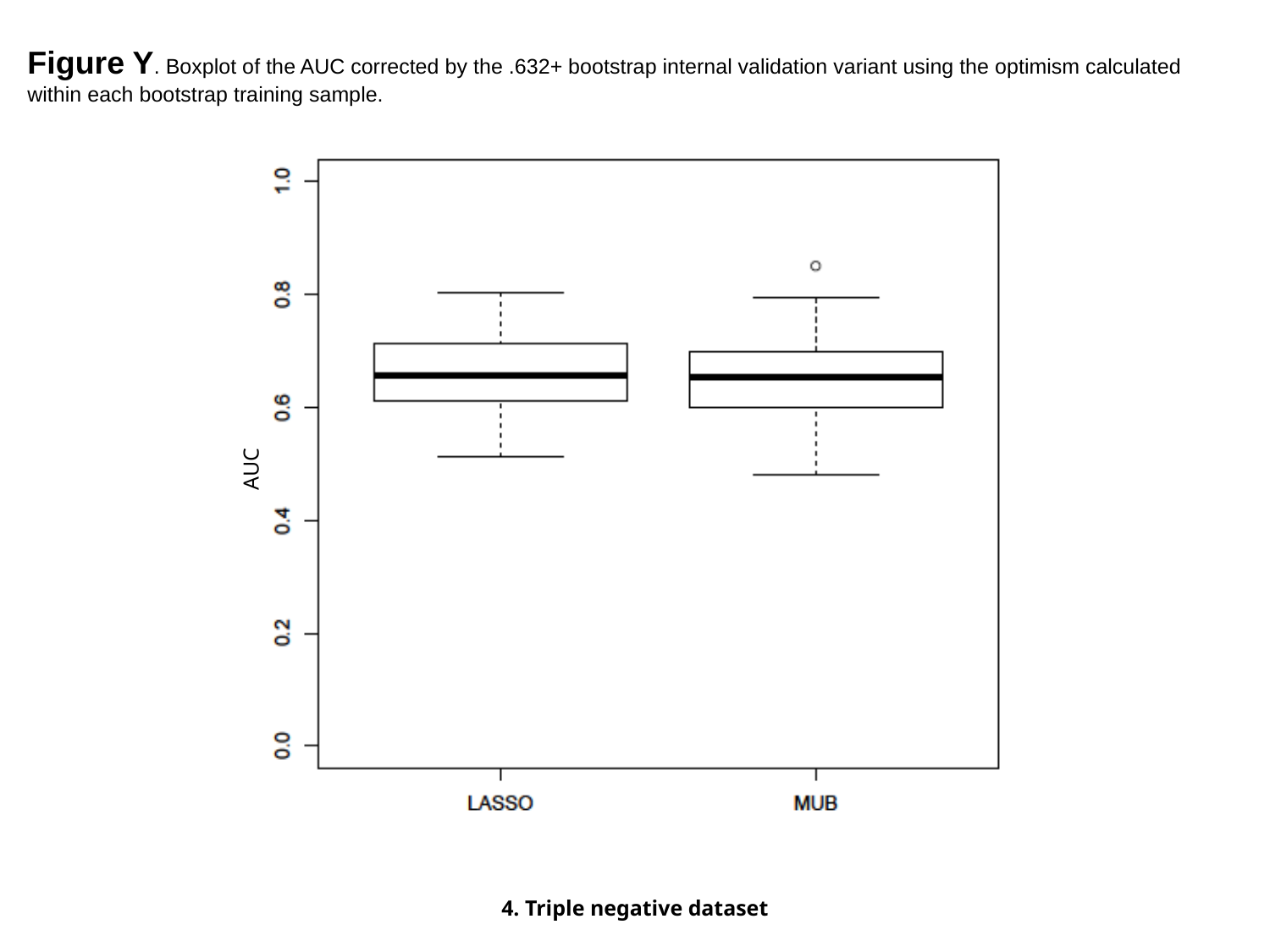

Figure Y. Boxplot of the AUC corrected by the .632+ bootstrap internal validation variant using the optimism calculated within each bootstrap training sample.
AUC
4. Triple negative dataset

## Slide 30
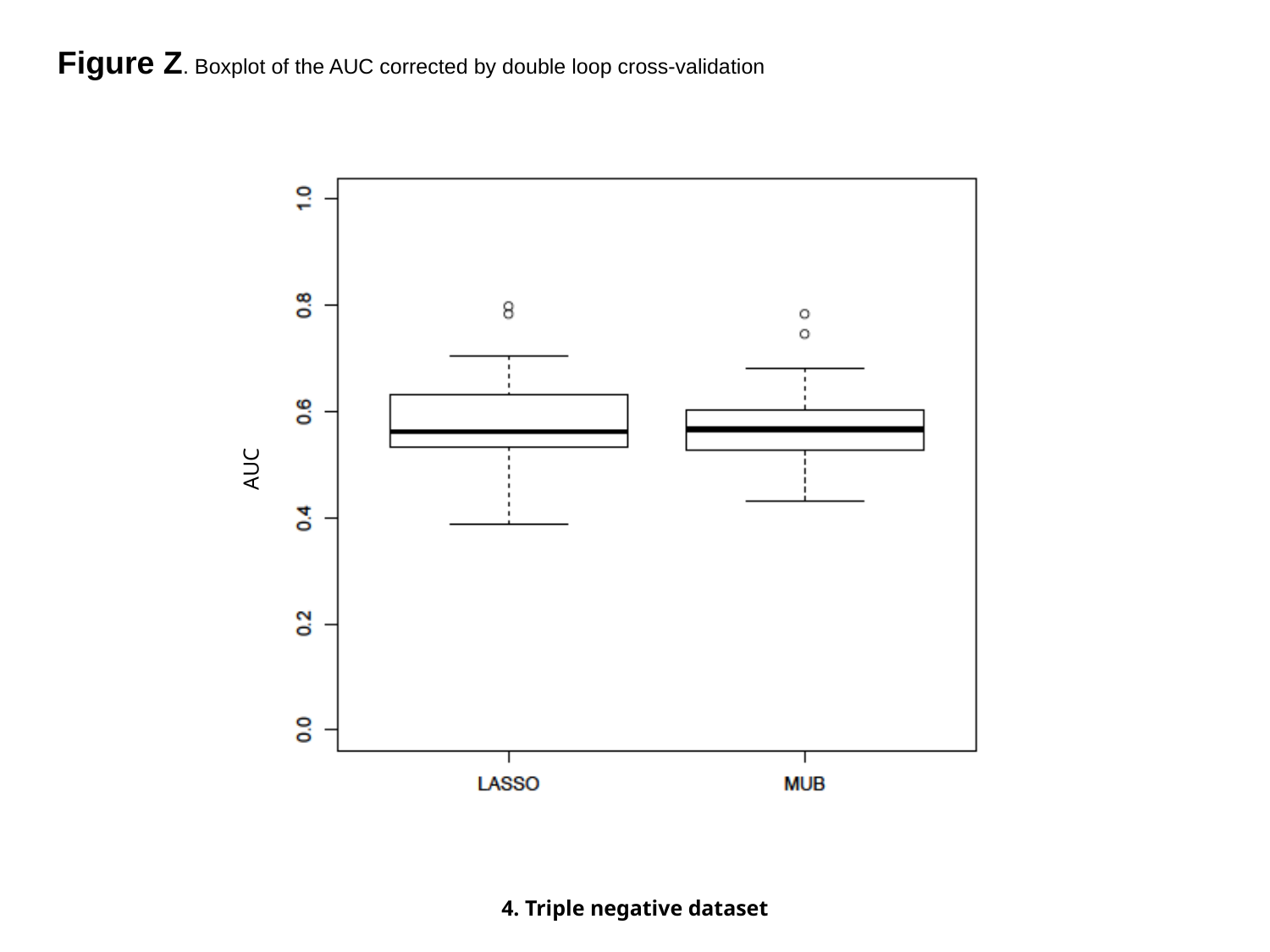

Figure Z. Boxplot of the AUC corrected by double loop cross-validation
AUC
4. Triple negative dataset

## Slide 31
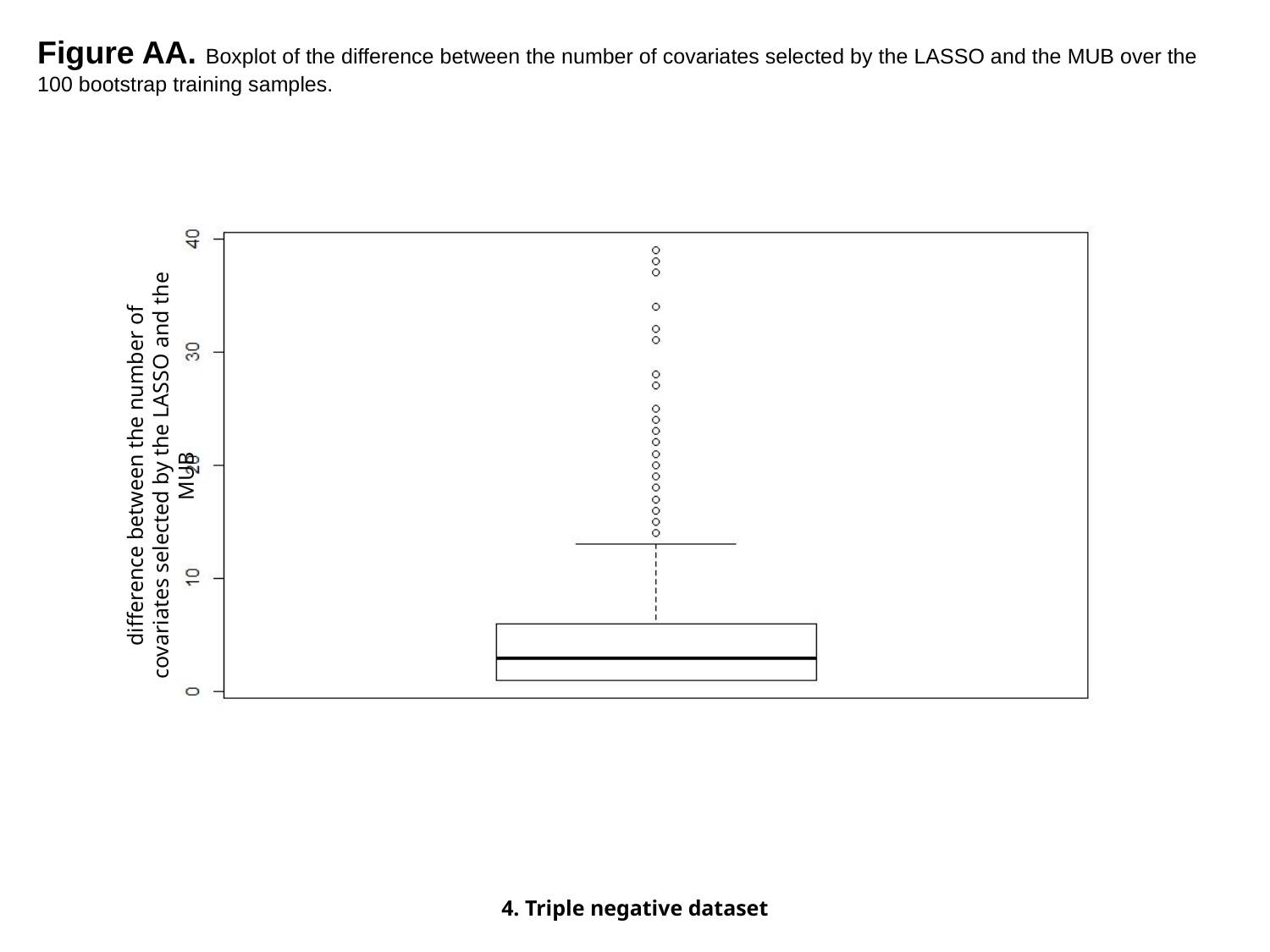

Figure AA. Boxplot of the difference between the number of covariates selected by the LASSO and the MUB over the 100 bootstrap training samples.
difference between the number of covariates selected by the LASSO and the MUB
4. Triple negative dataset

## Slide 32
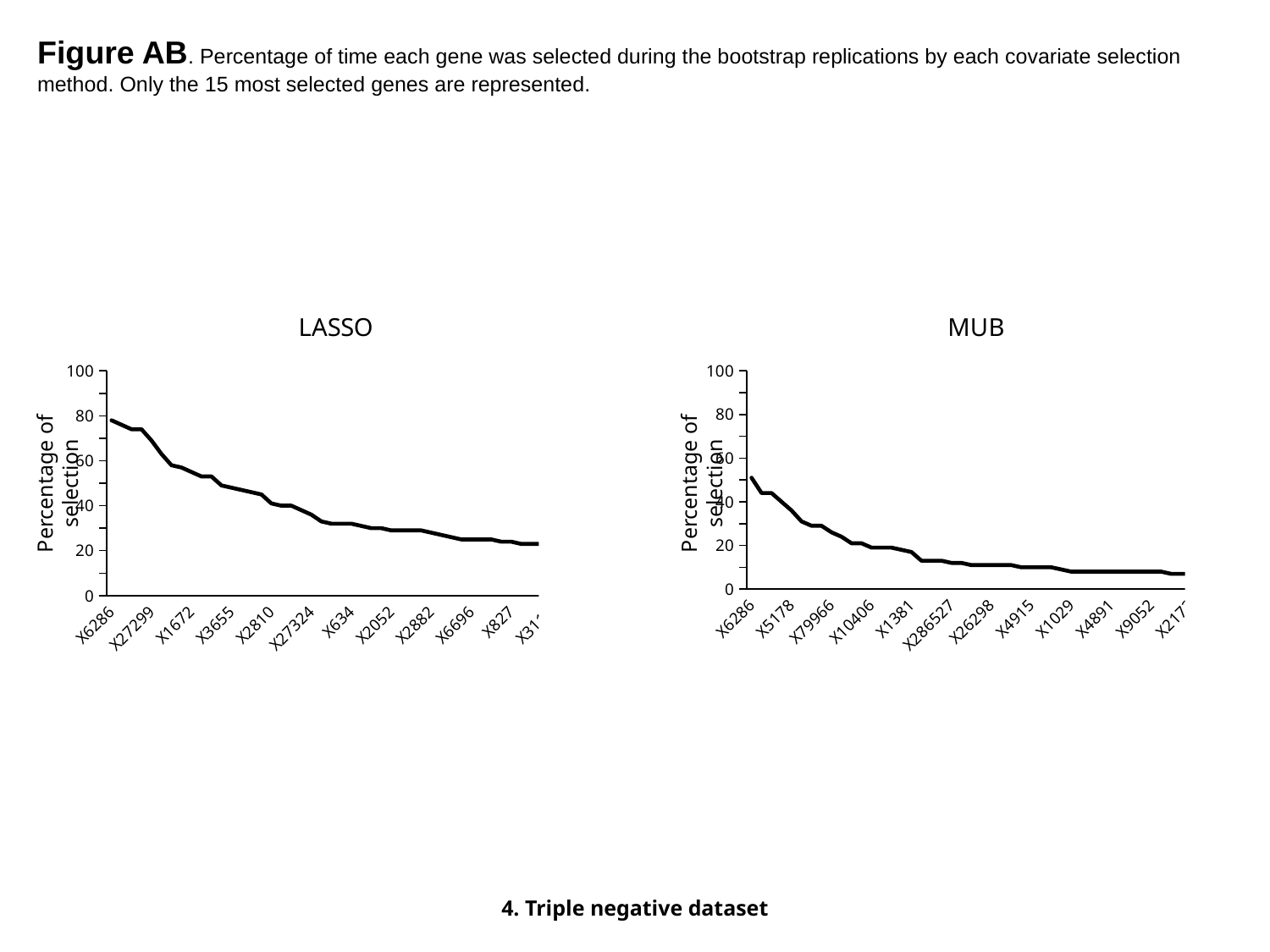

Figure AB. Percentage of time each gene was selected during the bootstrap replications by each covariate selection method. Only the 15 most selected genes are represented.
### Chart:
| Category | LASSO |
|---|---|
| X6286 | 78.0 |
| X563 | 76.0 |
| X5178 | 74.0 |
| X6273 | 74.0 |
| X27299 | 69.0 |
| X8788 | 63.0 |
| X10406 | 58.0 |
| X1278 | 57.0 |
| X1672 | 55.0 |
| X10551 | 53.0 |
| X79966 | 53.0 |
| X5918 | 49.0 |
| X3655 | 48.0 |
| X4915 | 47.0 |
| X6715 | 46.0 |
| X81569 | 45.0 |
| X2810 | 41.0 |
| X1381 | 40.0 |
| X51442 | 40.0 |
| X3576 | 38.0 |
| X27324 | 36.0 |
| X1830 | 33.0 |
| X26298 | 32.0 |
| X5320 | 32.0 |
| X634 | 32.0 |
| X140597 | 31.0 |
| X286527 | 30.0 |
| X9052 | 30.0 |
| X2052 | 29.0 |
| X347 | 29.0 |
| X55765 | 29.0 |
| X6590 | 29.0 |
| X2882 | 28.0 |
| X7018 | 27.0 |
| X1277 | 26.0 |
| X22943 | 25.0 |
| X6696 | 25.0 |
| X7837 | 25.0 |
| X79083 | 25.0 |
| X4604 | 24.0 |
| X827 | 24.0 |
| X1029 | 23.0 |
| X1728 | 23.0 |
| X26577 | 23.0 |
| X3117 | 23.0 |
| X4435 | 23.0 |
| X5806 | 23.0 |
| X7503 | 23.0 |
| X11013 | 22.0 |
| X2173 | 22.0 |
### Chart:
| Category | MUB |
|---|---|
| X6286 | 51.0 |
| X27299 | 44.0 |
| X563 | 44.0 |
| X6273 | 40.0 |
| X5178 | 36.0 |
| X8788 | 31.0 |
| X1278 | 29.0 |
| X3655 | 29.0 |
| X79966 | 26.0 |
| X6715 | 24.0 |
| X1672 | 21.0 |
| X81569 | 21.0 |
| X10406 | 19.0 |
| X10551 | 19.0 |
| X2810 | 19.0 |
| X5918 | 18.0 |
| X1381 | 17.0 |
| X1830 | 13.0 |
| X27324 | 13.0 |
| X51442 | 13.0 |
| X286527 | 12.0 |
| X79083 | 12.0 |
| X11013 | 11.0 |
| X140597 | 11.0 |
| X26298 | 11.0 |
| X55765 | 11.0 |
| X7018 | 11.0 |
| X3576 | 10.0 |
| X4915 | 10.0 |
| X7162 | 10.0 |
| X7837 | 10.0 |
| X2052 | 9.0 |
| X1029 | 8.0 |
| X1728 | 8.0 |
| X2167 | 8.0 |
| X4781 | 8.0 |
| X4891 | 8.0 |
| X595 | 8.0 |
| X634 | 8.0 |
| X827 | 8.0 |
| X9052 | 8.0 |
| X9244 | 8.0 |
| X1277 | 7.0 |
| X1848 | 7.0 |
| X2173 | 7.0 |
| X347 | 7.0 |
| X5806 | 7.0 |
| X7503 | 7.0 |
| X80162 | 7.0 |
| X2564 | 6.0 |
Percentage of selection
Percentage of selection
4. Triple negative dataset

## Slide 33
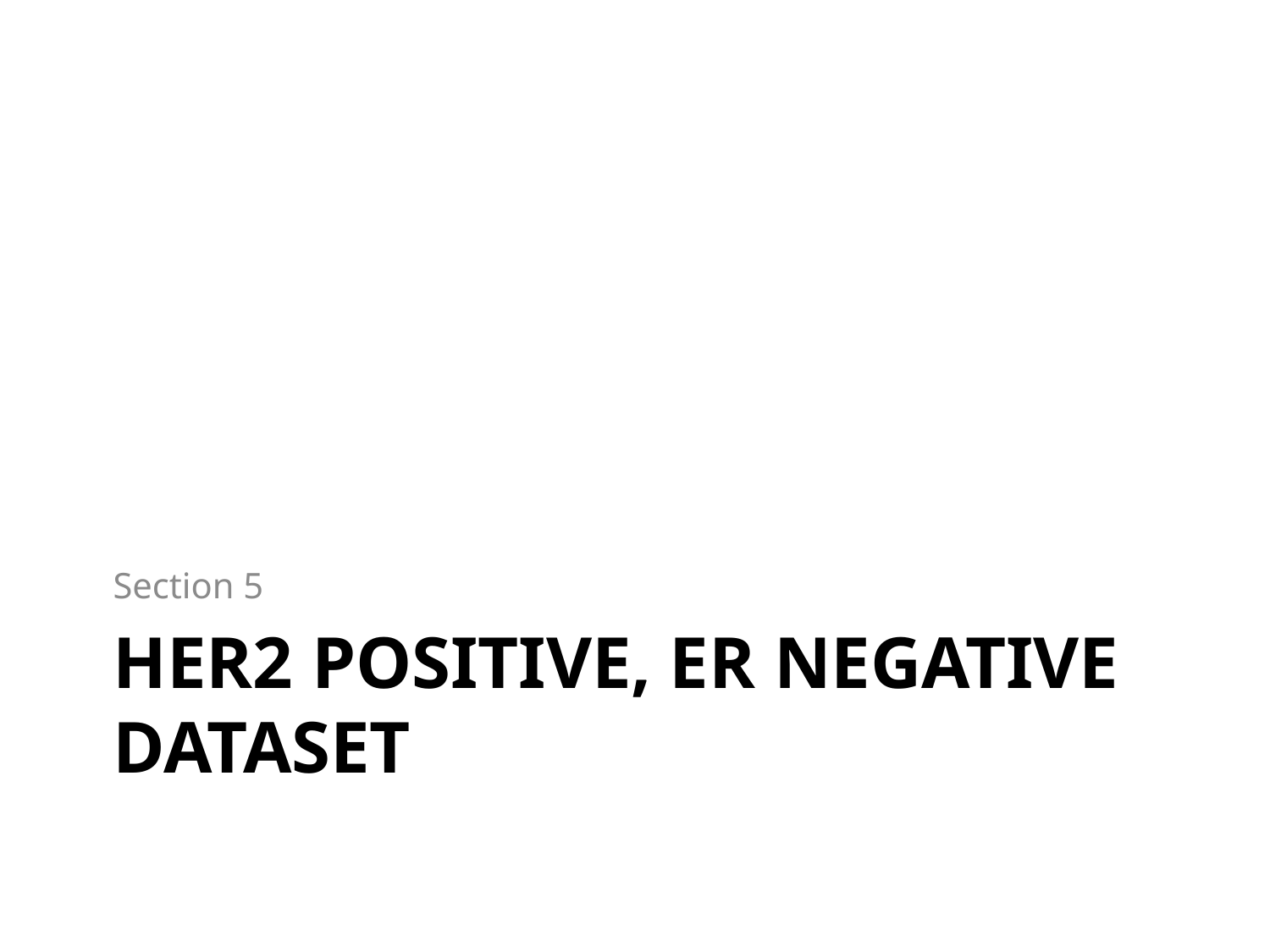

Section 5
# HER2 positive, ER negative dataset

## Slide 34
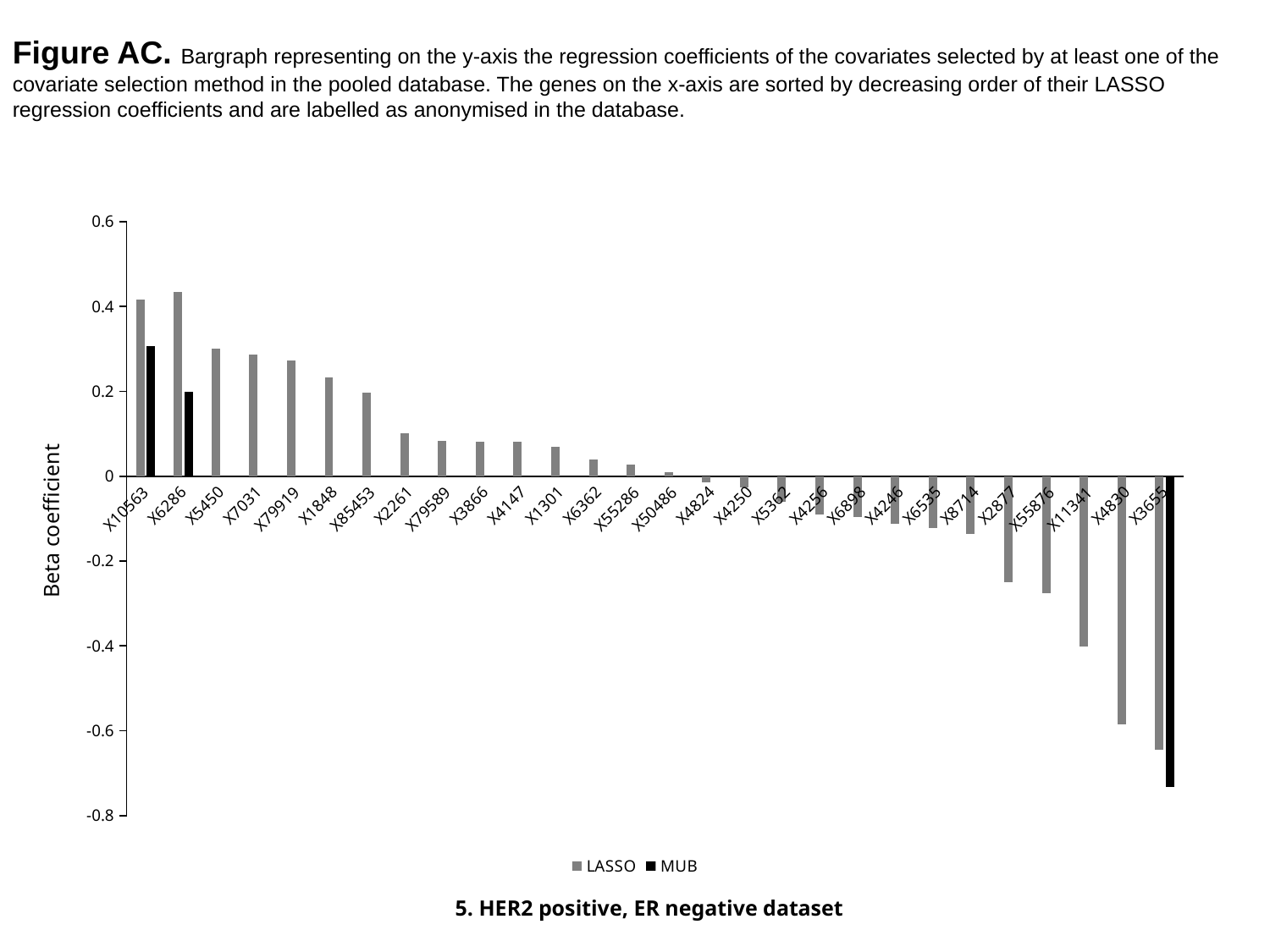

Figure AC. Bargraph representing on the y-axis the regression coefficients of the covariates selected by at least one of the covariate selection method in the pooled database. The genes on the x-axis are sorted by decreasing order of their LASSO regression coefficients and are labelled as anonymised in the database.
### Chart
| Category | LASSO | MUB |
|---|---|---|
| X10563 | 0.417239369758808 | 0.306291271960554 |
| X6286 | 0.433726528649391 | 0.199611612058031 |
| X5450 | 0.299963663824139 | 0.0 |
| X7031 | 0.286920250830119 | 0.0 |
| X79919 | 0.27241469169356 | 0.0 |
| X1848 | 0.232517161643158 | 0.0 |
| X85453 | 0.196331794224645 | 0.0 |
| X2261 | 0.100315873649326 | 0.0 |
| X79589 | 0.0837080448557958 | 0.0 |
| X3866 | 0.081707620345923 | 0.0 |
| X4147 | 0.080444465348632 | 0.0 |
| X1301 | 0.070244784328315 | 0.0 |
| X6362 | 0.0387938326733826 | 0.0 |
| X55286 | 0.0272405330248553 | 0.0 |
| X50486 | 0.00873519153884047 | 0.0 |
| X4824 | -0.0137085187562827 | 0.0 |
| X4250 | -0.027354673431692 | 0.0 |
| X5362 | -0.060231537137268 | 0.0 |
| X4256 | -0.090257515402407 | 0.0 |
| X6898 | -0.0966576117810868 | 0.0 |
| X4246 | -0.112308155956007 | 0.0 |
| X6535 | -0.12186222170788 | 0.0 |
| X8714 | -0.135252577269147 | 0.0 |
| X2877 | -0.250350294100667 | 0.0 |
| X55876 | -0.275611990151095 | 0.0 |
| X11341 | -0.401372410505261 | 0.0 |
| X4830 | -0.584554419538268 | 0.0 |
| X3655 | -0.644542490706321 | -0.733263712969014 |Beta coefficient
5. HER2 positive, ER negative dataset

## Slide 35
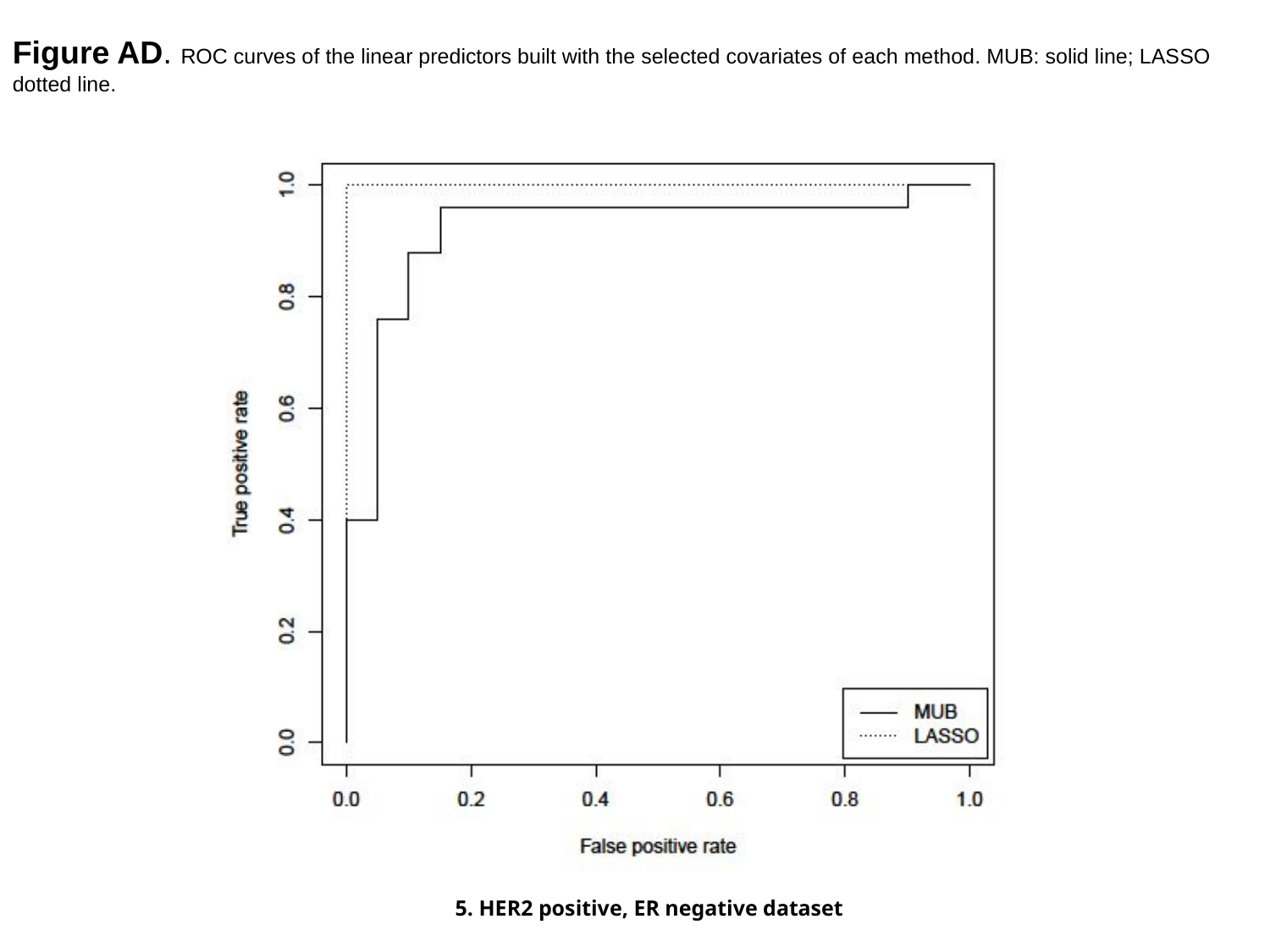

Figure AD. ROC curves of the linear predictors built with the selected covariates of each method. MUB: solid line; LASSO dotted line.
5. HER2 positive, ER negative dataset

## Slide 36
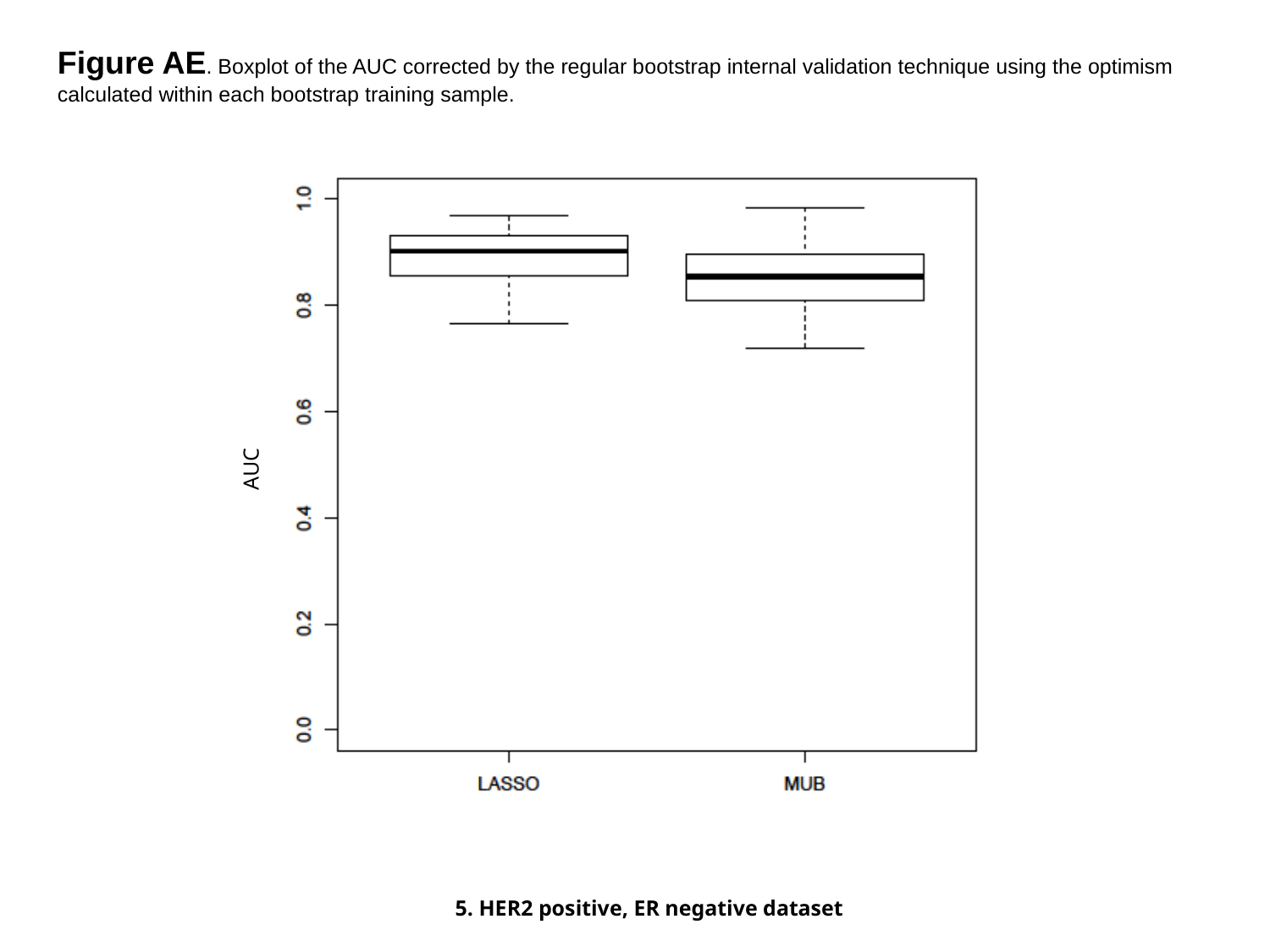

Figure AE. Boxplot of the AUC corrected by the regular bootstrap internal validation technique using the optimism calculated within each bootstrap training sample.
AUC
5. HER2 positive, ER negative dataset

## Slide 37
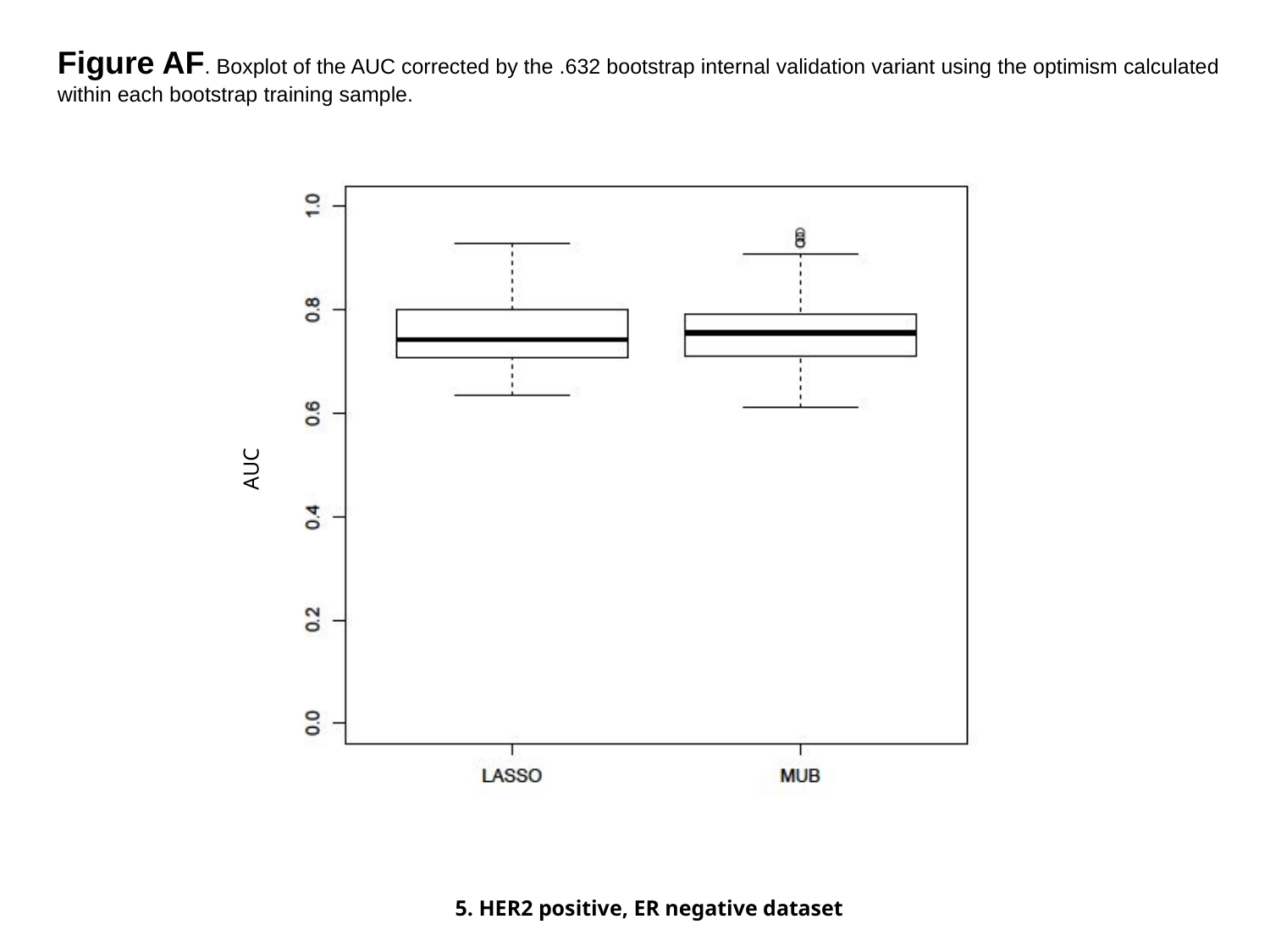

Figure AF. Boxplot of the AUC corrected by the .632 bootstrap internal validation variant using the optimism calculated within each bootstrap training sample.
AUC
5. HER2 positive, ER negative dataset

## Slide 38
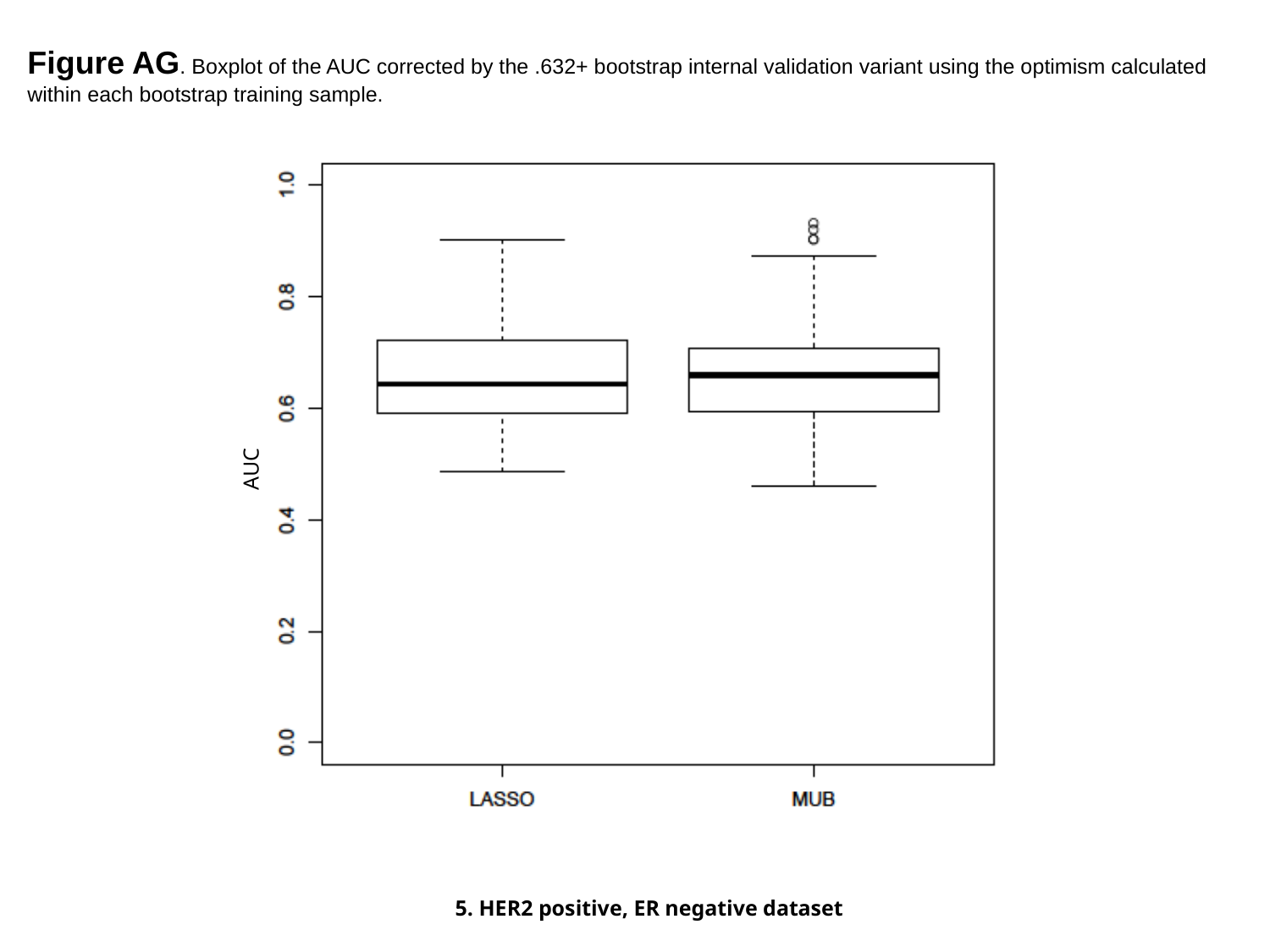

Figure AG. Boxplot of the AUC corrected by the .632+ bootstrap internal validation variant using the optimism calculated within each bootstrap training sample.
AUC
5. HER2 positive, ER negative dataset

## Slide 39
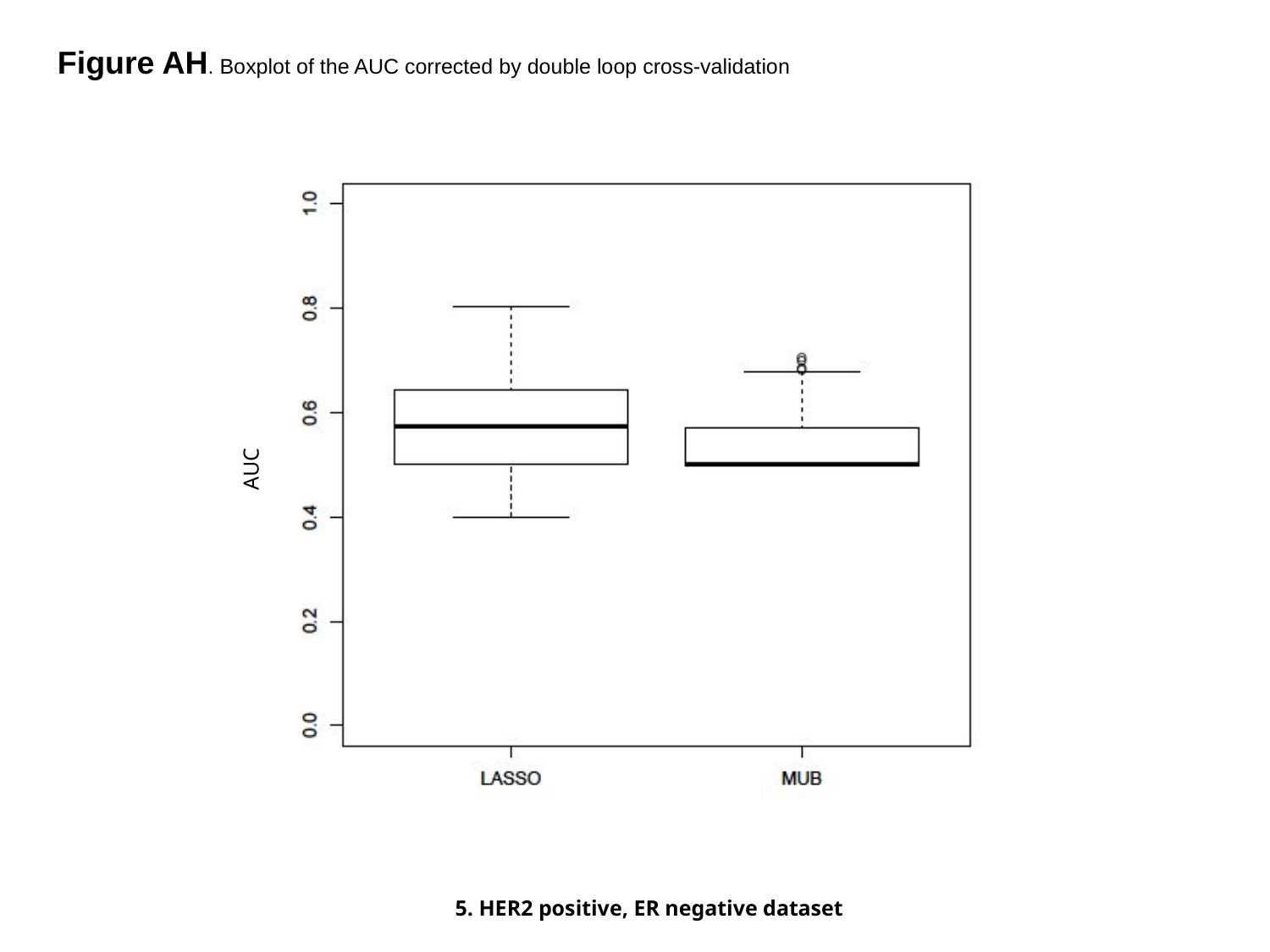

Figure AH. Boxplot of the AUC corrected by double loop cross-validation
AUC
5. HER2 positive, ER negative dataset

## Slide 40
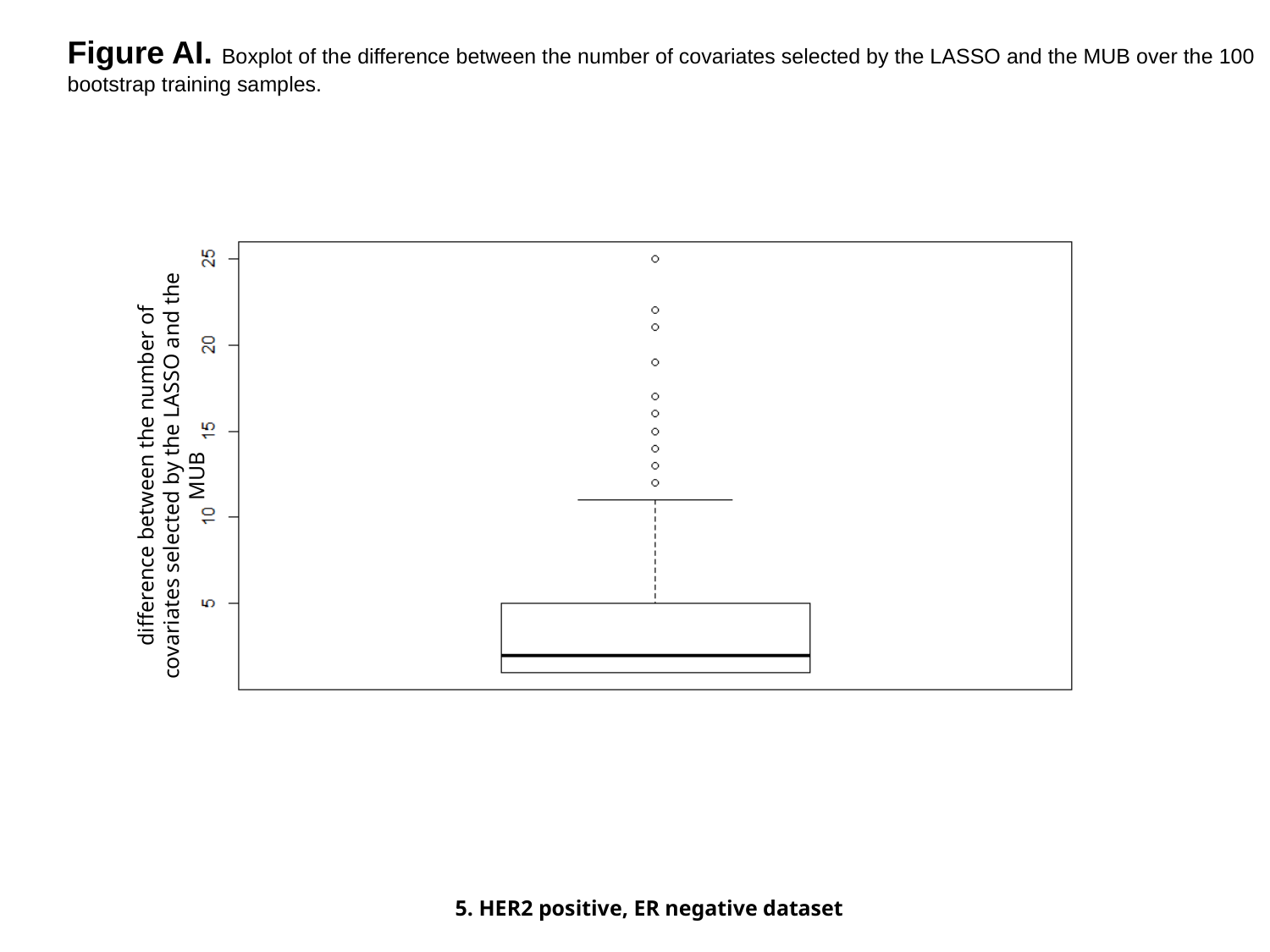

Figure AI. Boxplot of the difference between the number of covariates selected by the LASSO and the MUB over the 100 bootstrap training samples.
difference between the number of covariates selected by the LASSO and the MUB
5. HER2 positive, ER negative dataset

## Slide 41
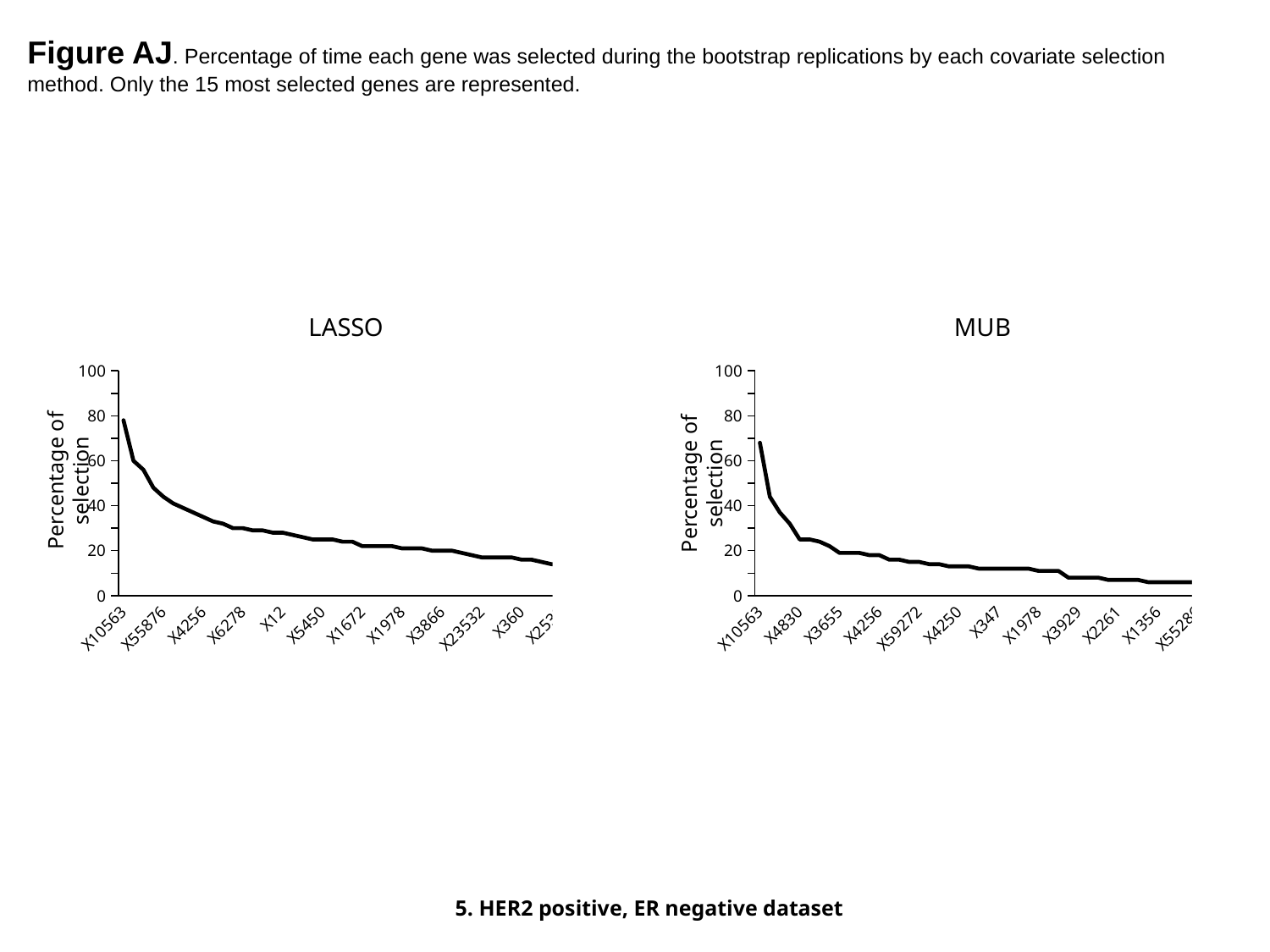

Figure AJ. Percentage of time each gene was selected during the bootstrap replications by each covariate selection method. Only the 15 most selected genes are represented.
### Chart:
| Category | LASSO |
|---|---|
| X10563 | 78.0 |
| X7031 | 60.0 |
| X6286 | 56.0 |
| X6362 | 48.0 |
| X55876 | 44.0 |
| X4830 | 41.0 |
| X2877 | 39.0 |
| X6898 | 37.0 |
| X4256 | 35.0 |
| X79919 | 33.0 |
| X85453 | 32.0 |
| X1448 | 30.0 |
| X6278 | 30.0 |
| X1301 | 29.0 |
| X3655 | 29.0 |
| X10321 | 28.0 |
| X12 | 28.0 |
| X4250 | 27.0 |
| X6279 | 26.0 |
| X23089 | 25.0 |
| X5450 | 25.0 |
| X80736 | 25.0 |
| X1475 | 24.0 |
| X9862 | 24.0 |
| X1672 | 22.0 |
| X347 | 22.0 |
| X4824 | 22.0 |
| X8842 | 22.0 |
| X1978 | 21.0 |
| X4147 | 21.0 |
| X59272 | 21.0 |
| X2261 | 20.0 |
| X3866 | 20.0 |
| X4477 | 20.0 |
| X4680 | 19.0 |
| X253190 | 18.0 |
| X23532 | 17.0 |
| X4246 | 17.0 |
| X5304 | 17.0 |
| X8714 | 17.0 |
| X360 | 16.0 |
| X55286 | 16.0 |
| X3929 | 15.0 |
| X11341 | 14.0 |
| X2539 | 14.0 |
| X3212 | 14.0 |
| X81569 | 14.0 |
| X10202 | 13.0 |
| X1848 | 13.0 |
| X2019 | 13.0 |
### Chart:
| Category | MUB |
|---|---|
| X10563 | 68.0 |
| X7031 | 44.0 |
| X6286 | 37.0 |
| X6362 | 32.0 |
| X4830 | 25.0 |
| X6898 | 25.0 |
| X2877 | 24.0 |
| X79919 | 22.0 |
| X3655 | 19.0 |
| X55876 | 19.0 |
| X6278 | 19.0 |
| X1448 | 18.0 |
| X4256 | 18.0 |
| X10321 | 16.0 |
| X6279 | 16.0 |
| X4477 | 15.0 |
| X59272 | 15.0 |
| X253190 | 14.0 |
| X5450 | 14.0 |
| X1672 | 13.0 |
| X4250 | 13.0 |
| X80736 | 13.0 |
| X12 | 12.0 |
| X23089 | 12.0 |
| X347 | 12.0 |
| X3866 | 12.0 |
| X4147 | 12.0 |
| X9862 | 12.0 |
| X1978 | 11.0 |
| X6696 | 11.0 |
| X85453 | 11.0 |
| X3212 | 8.0 |
| X3929 | 8.0 |
| X4246 | 8.0 |
| X8714 | 8.0 |
| X1301 | 7.0 |
| X2261 | 7.0 |
| X23532 | 7.0 |
| X4824 | 7.0 |
| X11341 | 6.0 |
| X1356 | 6.0 |
| X2539 | 6.0 |
| X50486 | 6.0 |
| X5304 | 6.0 |
| X55286 | 6.0 |
| X5788 | 6.0 |
| X81569 | 6.0 |
| X8842 | 6.0 |
| X10202 | 5.0 |
| X1475 | 5.0 |
Percentage of selection
Percentage of selection
5. HER2 positive, ER negative dataset
